# The impact of the flipped classroom on the motivation and academic performance of Chinese college English learners

**DOI:** 10.1371/journal.pone.0322094

**Published:** 2025-05-02

**Authors:** Zhonger Wang, Jin Lu, Shenchen Yu

**Affiliations:** School of Foreign Languages, Shanghai Lixin University of Accounting and Finance, Shanghai, China; Ahvaz Jundishapur University: Ahvaz Jondishapour University of Medical Sciences, IRAN, ISLAMIC REPUBLIC OF

## Abstract

The flipped classroom exemplifies the concept of active teaching; nevertheless, its effects on learning motivation and academic performance remain somewhat inconsistent. In this research, a sample of 70 sophomore students from China was selected and divided into an experimental group (comprising 35 students) and a control group (also 35 students). A four-month quasi-experiment was then conducted, with the experimental group implementing the flipped classroom teaching approach, while the control group adhered to the traditional teaching method. Before and after the experiment, a questionnaire covering four factors, which was determined through exploratory and confirmatory factor analyses of 611 college students, was used to measure the changes in students’ English learning motivation, and two English proficiency tests of the same difficulty level were also conducted. The research results of the independent *t* test between the two groups showed that the overall motivation level of the experimental group was significantly improved. Specifically, in terms of integrative motivation, the mean value of the post-test in the experimental class was 3.53, with a standard deviation (SD) of 0.90, a *t*-value of 2.651, a *P*-value of 0.010* and a Cohen’s *d* of 0.634; in terms of instrumental motivation, the mean value of the post-test was 3.34, with a SD of 0.91, a *t*-value of 2.881, a *P*-value of 0.005** and a Cohen’s *d* of 0.689; in terms of autonomous learning motivation, the mean value of the post-test was 3.84, with a SD of 0.78, a *t*-value of 5.569, a *P*-value of 0.000**and a Cohen’s *d* of 1.331; in terms of intrinsic motivation, the mean value of the post-test was 3.58, with a SD of 0.94, a *t*-value of2.889, a *P*-value of 0.005**and a Cohen’s *d* of 0.691; from the perspective of total motivation, the mean value of the post-test was 3.58, with a SD of 0.69, a *t*-value of 4.477, a *P*-value of 0.000**and a Cohen’s *d* of 1.070. Meanwhile, the total score of the English test in the experimental group was also significantly increased in the independent *t* test. The mean value of the post-test in the experimental class was 68.06, with a SD of 8.00, a *t*-value of 3.336, and a *P*-value of 0.001**and a Cohen’s *d* of 0.797. Through correlation analysis, it was found that the above four factors had a significant positive correlation with English scores. Further multivariate linear regression analysis was carried out, taking the four motivation factors as independent variables and English scores as dependent variables. The results showed that autonomous learning motivation (*P* = 0.022* < 0.05) and intrinsic motivation (*P* = 0.001** < 0.01) made significant contributions to predicting English scores, and the variance inflation factor (VIF) value was less than 5, indicating that there was no multicollinearity problem. In conclusion, this study confirms that the flipped classroom can effectively stimulate students’ English learning motivation and improve their scores. Accordingly, it is recommended to focus on cultivating students’ autonomous learning motivation and intrinsic learning motivation in the teaching process.

## 1. Introduction

In the current era of rapid globalization and digitalization, the continuous innovation of information technology has exerted a profound impact on the field of education [1]. Traditional teaching methods are now confronted with numerous unprecedented challenges within this wave of transformation [2]. Particularly in the realm of college English teaching in China, the long-standing issue of students’ insufficient enthusiasm and participation has emerged as a crucial factor impeding the enhancement of teaching quality [3]. Based on the findings of the investigations and research conducted in college English classrooms across multiple Chinese universities, it is revealed that approximately 75% of students remain in a passive learning mode during traditional classroom instruction, seldom taking the initiative to pose questions or engage in classroom discussions [4]. The principal reason for this lies in the fact that the traditional teaching paradigm is predominantly centered on teachers’ lecturing, thereby depriving students of ample opportunities for independent expression and practice [5].Simultaneously, certain studies have indicated that with the ever-increasing demands of society regarding the English proficiency of compound talents, students’ requirements for English learning have become increasingly diverse [6,7]. They anticipate obtaining more personalized guidance, experiencing real language contexts, and seizing opportunities for cross-cultural communication in the process of English learning. Nevertheless, the relative monotony of traditional teaching modes, both in terms of teaching content and teaching methods, makes it arduous to fulfill these diverse demands [8].

In recent years, the flipped classroom, an innovative educational model, has gradually emerged and drawn widespread attention [9]. By relocating the delivery of knowledge to the extracurricular realm and capitalizing on a wealth of online learning resources such as meticulously crafted teaching videos and highly interactive learning software, it empowers students to independently schedule their learning progress and attain personalized learning [10].Concurrently, classroom time is dedicated more to both in-depth interactions between teachers and students and collaborative learning within groups. This effectively spurs students’ active participation and facilitates the internalization of knowledge.For instance, in an empirical study on the college English flipped classroom in a particular university, it was discovered that following the implementation of the flipped classroom, the average number of times students took the initiative to speak in class increased by 50%, and the participation rate in group cooperation projects rose by 50% [11]. These findings suggest that this teaching model can significantly augment students’ learning initiative as well as their teamwork capabilities.

The present study endeavors to conduct an in-depth investigation into the influences of the flipped classroom on Chinese college students’ English learning motivation and academic achievements. By incorporating learning motivation theories, it undertakes a systematic analysis of the operational mechanism through which the flipped classroom stimulates English learning motivation and facilitates the elevation of learning outcomes.Although prior research has attained certain accomplishments concerning the application effects of the flipped classroom [12], this study will further concentrate on delving into the specific factors that impact students’ learning motivation within the flipped classroom paradigm along with their inherent relationships, and how these factors exert an influence on academic achievements.Through rigorous empirical research methodologies, this study is anticipated to offer more targeted and practicable theoretical support and practical guidance for the enhancement of college English teaching in China. Simultaneously, it also contributes to enriching the research findings of the flipped classroom in the field of language learning.

## 2. Literature review

### 2.1. The relationship between the flipped classroom and active teaching approaches

In recent years, the traditional teaching model has fallen into dispute. For example, as teachers hold the dominant position in the classroom, students passively acquire knowledge and lack the chances to actively engage in classroom interaction and independent thinking [13]. Hence, the active teaching method has emerged, which is studentcentered, emphasizing students’ active participation and proactive exploration during the learning process [14].

The active teaching approach highlights students’ initiative and focuses on the cultivation of their self-directed learning, problem-solving and innovative thinking. The implementation of the flipped classroom fully reflects the notion of the active teaching approach. When students do autonomous learning before class, they need to be active. Besides, the interactive communication and group tasks in class also require their active participation and proactive thinking [15].

It can be stated that the flipped classroom is a specific application format of the active teaching approach. Through the re-design of the teaching process, it creates favorable circumstances for the implementation of the active teaching, allowing students more profound participation in the learning process and accomplishment of the transformation from passive acceptance to active exploration of knowledge [16].

### 2.2. Types of learning motivation

Motivation is commonly defined as the internal force or driving power that propels an individual to take action to reach a specific goal [17]. Learning motivation refers to a kind of dynamic propensity that initiates and sustains students’ learning behaviors and orients them towards certain academic objectives [18]. Specifically in English learning, motivation can considerably affect students’ attitudes, the selection of learning strategies, and the ultimate result of English learning [19]. The following will explore the main categories of learning motivation and their roles in English learning.

#### 2.2.1. Instrumental motivation.

Instrumental learning motivation refers to the learning motivation that learners generate in order to attain certain practical purposes, such as passing examinations, acquiring professional qualifications, fulfilling work demandsand so on [20]. This type of motivation is typically extrinsic and short-term, focusing on achieving specific targets or obtaining practical benefits. Individuals with this kind of motivation are more concerned about the direct rewards and practical applications that the learning outcomes can yieldrather than the inherent interest and love for knowledge per se.

Instrumental learning motivation for English refers to the one for learning English that learners develop in an effort to achieve certain practical purposes related to English, acquire practical benefits, or satisfy external demands [21]. This motivation is mainly driven by the following four factors [22]. Firstly, examinations: students painstakingly memorize words and grammar rules in order to pass the English course examinations at school. Secondly, parental expectations: when parents have high expectations for their children in English learning such as expecting their children to get excellent grades in English examinations, obtaining better job opportunities or studying abroad in the future with good English proficiency, their children may internalize these expectations as their own learning motivation and form a relatively strong instrumental learning motivation for English. They will regard learning English well as an important way to fulfill their parents’ expectations, gain their parents’ appreciation, and thus study English grammar and vocabulary more diligently and actively prepare for English examinations, etc. Thirdly, teachers’ requirements: they work hard at English to hand in the assignments on time or complete other specific learning tasks. Fourthly, work tasks: for instance, students need to write reports in English or reply to emails, etc., thereby temporarily increasing their investment in English learning.

#### 2.2.2. Integrative motivation.

Integrative motivation indicates that learners possess a strong desire to acquire a target language because of their fondness of its related culture and their willingness to communicate with the target language community and integrate into its culture [23]. Such motivation typically stems from a positive attitude towards the culture of the target language instead of pragmatic purposes.

Specifically, integrative motivation is manifested in the following three aspects [24]:

1. Cultural interest: Learners display a strong interest in the culture of English-speaking countriessuch as movies, music, art, literature, history, and customs. They are eager to obtain a deep understanding and experience. These elements fuel their motivation to learn English.
2. Social needs: Learners desire to communicate fluently with native English speakers or those proficient in English, expand their social circles, and integrate into a diverse language and cultural environment.
3. Identity recognition: Learners consider themselves members of the global village and believe that proficient mastery of English is a necessary condition for shaping a multicultural identity.

The main distinctions between integrative learning motivation and instrumental learning motivation are as follows.

1. Learners driven by instrumental motivation often strive to achieve certain short-term and specific goalssuch as passing examinations, obtaining certificates, and fulfilling work requirements. Students possessing integrative motivation aspire to blend into and understand the cultural groups of the target language and have long-term communication with them.
2. Students with instrumental motivation have relatively less interest in the learning content itself and pay more attention to the practical benefits brought by the learning results. On the contrary, students with integrative motivation place greater emphasis on the in-depth experience of the culture of the target language.

#### 2.2.3. Intrinsic motivation.

Intrinsic motivation for English learning refers to the positive and proactive drive for learning English that learners generate owing to their own passion for the language itself or the sense of achievement gained from the process of learning English and other internal factors [25]. This type of motivation does not arise from external rewards, pressure or demands; instead, it is a spontaneous and inherent driving force. This urges learners to voluntarily devote time and energy to learning English and constantly enhance their English proficiency.

Intrinsic motivation for learning English can be manifested in three aspects [26]. The first is willingness to embrace challenges. Students are not intimidated by the difficulties in learning English; rather, they consider them as opportunities for self-improvement. The second is active enhancement of thinking. When it comes to communication in English, learners need to organize the language and express thoughts clearly, which can boost the ability of language organization and expression. As for reading, English literary works or academic articles enable English learners to be exposed to diverse viewpoints and ideas, stimulate critical thinking, and prompt them to evaluate, question, and think innovatively about the information. Meanwhile, understanding the cultural background and thinking patterns of English-speaking countries as well as comparing and integrating them with learners’ own culture is conducive to cultivating cross-cultural thinking and multiple perspectives and expanding the breadth and depth of their thinking. The third aspect is taking learning as enjoyment--- English learners do not view learning English as a burden.

There are four differences between integrative motivation and intrinsic motivation in terms of English learning.First of all, the driving factors are different. Integrative motivation for English is mainly driven by the interest in the culture carried by the English language and the eagerness to integrate into the English cultural group. Intrinsic motivation for English, on the other hand, places more emphasis on obtaining pleasure, satisfying curiosity, pursuing self-growth and ability improvement from the act of learning English itself.Secondly, their stabilities vary.Integrative motivation for English may fluctuate as learners’ notion about English cultural group varies. Intrinsic motivation is relatively more stable since it stems from the individual’s inherent love and need for learning.Thirdly, the choice of learning content is not the same. Integrative motivation for English may prompt learners to pay more attention to culturesuch as literature, art, customs and habits, etc. Intrinsic motivation may cause learners to show enthusiasm in all aspects of English learning, including the comprehensive improvement of language knowledge and skills.Finally, the goal orientation is distinct. Learners with integrative motivation for English usually aim to communicate freely in the English cultural environment. The goal of learners driven by intrinsic motivation may be more inclined to constantly surpass their own English proficiency.

#### 2.2.4. Autonomous learning motivation.

Autonomous learning motivation for English is the internal and proactive driving force and willingness that urges learners to engage in English learning independently [27].

The type of motivation can be manifested in the following six aspects [28]. Firstly, set goals actively. Learners should have a clear perception of the English proficiency they aspire to reach and break it down into specific and measurable targets.Secondly, plan learning actively. Students need to arrange their learning time, progress and content independently and formulate detailed learning plans.Thirdly, conduct self-monitoring and evaluation. Learners are supposed to inspect their learning progress regularly and carry out self-assessment of the learning outcome.Fourthly, participate actively in English activities. This helps to enhance their English proficiency.Fifthly, seek appropriate learning methods. Learners should actively explore various possible learning approaches to fulfill their learning goals.Finally, practice self-motivation. When learners encounter learning obstacles, they must not give up easily but sustain their enthusiasm for learning.

### 2.3. Different roles of flipped classroom in learning

Studies have indicated the positive impacts of flipped classrooms on learning motivation. This is chiefly reflected in two aspects: enhancing autonomous learning motivation and raising interest [29].Regarding autonomous learning motivation, flipped classrooms empower students to independently manage their learning time and progress outside the classroom, thereby fostering their autonomous learning ability. Students can acquire knowledge in accordance with their own pace and needs, which grants them a sense of control over learning and subsequently sparks stronger learning motivation.Concerning raising interest, thanks to the abundant and diverse online learning resourcessuch as vivid and engaging teaching videos and interactive learning software, knowledge can be presented in a more appealing manner. This makes learning more enjoyable and stimulates students’ curiosity and eagerness for exploration [30].

However, other studies have indicated that flipped classrooms might have certain negative effects on learning motivation in terms of autonomous learning and stress [31]. Regarding autonomous learning, some students might lack the ability and self-discipline for self-regulated study. In the flipped classroom model, students are required to independently watch teaching videos and complete preview tasks outside the classroom. However, if students do not possess good self-management skills, they are very likely to procrastinate and be casual in preview, resulting in an inadequate understanding of the course content and thus exerting a negative impact on learning motivation.Secondly, in terms of stress, the flipped classroom forces students to devote more time and energy to preview and preparation outside the classroom. If students’ own academic burden is already heavy, they might feel overburdened [32]. This additional learning pressure might cause students to feel fatigued, thereby weakening their learning motivation.

In terms of academic performance, some studies have demonstrated that flipped classrooms have a positive impact on learning achievements through various channels [33]. Flipped classrooms place emphasis on nurturing students’ abilities of independent exploration, cooperative learningand expression. During the discussions, group activities, and project presentations in the classroom, students’ thinking abilities, communication skills, and teamwork capabilities are exercised. The improvement of these comprehensive abilities helps students understand and apply knowledge more effectively and enhance their problem-solving abilities, which is manifested in the improvement of academic performance.On the other hand, flipped classrooms might have a negative influence on academic performance. For example, individual differences can lead to uneven learning progress. Different students have discrepancies in learning ability, learning habits, and family environment. Some students can complete pre-class independent learning efficiently, while some students may be unable to fully grasp knowledge for various reasons. When conducting discussions in the classroom, students with weak foundations may have difficulty keeping pace and be incapable of full participation, resulting in an increasing number of knowledge loopholes and ultimately having an effect on academic performance.

### 2.4. Research gap and questions

There exist extensive researches on the flipped classroom in China. Nevertheless, a considerable portion of these researches are not related to motivation. However, the research on student’motivation within the flipped classroom model can advance these studies. For example, Chang [34] investigated the characteristics of learning strategies under the flipped classroom model but did not address learning motivation. In reality, learning motivation holds a crucial role in learning strategies. A vigorous learning motivation can impel students to explore initiatively and adopt effective learning strategies. Different learning motivations will induce students to opt for distinct learning strategies. The research conducted by Zheng and Lee [35] merely encompassed teaching strategies and achievements without incorporating motivational factors. It is notable that learning motivation has a significant and diverse influence on academic performance. To begin with, a positive learning motivation can bestow students with a potent intrinsic driving force. When students possess clear learning goals and a strong aspiration, they will be more enthusiastically engaged in learning and be willing to expend more time and energy exploring knowledge, thereby enhancing academic performance. Hwang [36] merely pertained to students’ cognition, whose notion was that creative style develops their high-level cognitive abilities for the sake of achieving sustainable learning practices. It is worth understanding that motivation affects the breadth and depth of cognition. A higher level of motivation may enable individuals to collect information more extensively and delve deeper into the essence of the problem, while the absence of motivation may result in the superficiality and narrowness of cognition.

In the motivation research concerning the flipped classroom model in China, a number of studies centered on Chinese language learners in China [37,38] rather than the broader group of Chinese English learners. Some flipped classroom studies concentrating on Chinese English learners merely took primary school students rather than college students as the research subjects [39]. In the investigations of the flipped classroom with college students as the research subjects, some were solely restricted to qualitative analysis and did not conduct quantitative analysis. This imbalance in research approaches imposed certain limitations. Specifically, Suo and Hou [38] only explored the motivation strategies under the college English flipped classroom from a qualitative perspective. Some studies, although incorporating quantitative analysis, lacked the reliability test of the questionnaire. For instance, ZUO [40] adopted the questionnaire survey method, but the sample size was merely 25 students, and the study only involved frequency statistics, with neither validity analysis nor reliability analysis.

Although Justin Nicholes [41] reported the results of the reliability test of his questionnaire, the results of the validity test and exploratory factor analysis were not presented. For the assessment of motivational beliefs and strategies of self-regulated learning, this study adopted a modified Motivational Strategies for Learning Questionnaire (MSLQ) in Chinese version, comprising 44 items. The original scale was developed by Pintrich, Smith, Garcia, and McKeachie [42]. Nonetheless, this study regarding Chinese English learners was carried out in 2020, nearly 27 years after the development of Pintrich’s scale, and there were marked differences in language background and cultural background between the research subjects of his study and the group for which Pintrich’s scale was initially intended. Considering the diversity and cultural discrepancies of students, the authors appropriately adjusted the scale to better cater to the needs of specific student groups in China. However, after modifying the questionnaire items, it is typically not recommended to merely conduct reliability analysis. Furthermore, reliability analysis is mainly employed to evaluate the stability and consistency of measurement tools, but it cannot fully represent the validity and accuracy of the questionnaire. In addition to reliability analysis, validity analysis should also be implemented in that it can help determine whether the questionnaire actually measures the concept or variable it aims to measure. For example, if only reliability is focused on and validity is disregarded, the following situation may occur: the responses of the questionnaire are consistent, but in fact, the content related to the research topic is not accurately measured.

Some studies concentrating on the flipped classroom executed validity analysis, yet remained deficient in quantitative validity tests. For example, the formal questionnaire survey conducted by Li [43] was merely aimed at 69 students, featuring a relatively small sample size. Despite the invitation of two language education professors to review the content of the questionnaire to ensure content validity, this study still failed to guarantee the propriety of the validity of the questionnaire survey from the quantitative analysis perspective. Even if individual studies encompassed quantitative tests related to validity, they did not conduct research on specific motivational factors. For instance, the meta-analysis results of Zheng et al. [44] demonstrated that the flipped classroom teaching method had a moderate effect size on academic performance and learning motivation. Nevertheless, this study did not encompass the influence of the flipped classroom on specific motivational factors. Therefore, thiscurrent study intends to embrace a comprehensive quantitative analysis approach to determine the dimensions of students’ English learning motivation under the flipped classroom model and subsequently explore the following questions in order to clarify the impact of flipped classrooms on English learning motivation and English academic performance amid the controversy over the role of flipped classrooms.

1. Does the flipped classroom have a significant impact on the English learning motivation of Chinese college students?
2. Which factors in English learning motivation have predictive ability for English grades?
3. Will the flipped classroom model significantly influence English grades?

## 3. Methods

### 3.1. Research subjects

This research has successfully passed the ethical review of the Ethics Committee of the School of Foreign Languages at Shanghai Lixin University of Accounting and Finance, and was officially approved in August 2023. The research was generally divided into two distinct stages. In the first stage, efforts were dedicated to the development of the English learning motivation scale. In the second stage, the exploration was centered on the impact of the flipped classroom on English learning motivation and academic performance.

During the first stage of the research, on September 1st, 2023, the research team adopted a random sampling method to select 629 students from five universities in Shanghai China as the research objects. More precisely, the team carefully chose five representative universities from various higher education institutions with different levels and characteristics, such as comprehensive universities, science and engineering colleges, and finance and economics institutions, aiming to guarantee that the sample could adequately reflect the diversity of students across different universities.Subsequently, participants were randomly drawn from the student lists provided by each of these universities. In the sampling procedure, a computer-generated random number table was utilized by the research team to determine the specific student numbers to be sampled. This approach ensured the complete randomness of the sampling process, effectively eliminating any potential human interference.To further augment the representativeness of the sample, the research team allocated the number of samples to be drawn in proportion to the total number of students in each university. For instance, universities with a larger student population were assigned more sampling quotas, while those with a relatively smaller number of students were allocated fewer quotas accordingly. Moreover, during the random sampling process, an effort was made to cover different grades and majors as comprehensively as possible, thereby ensuring that the sample could thoroughly represent the diversity of students within each institution.Once the sample was finalized, the research team reached out to the students through offline notifications, inviting them to participate in the study. After all the participants had signed the written informed consent forms, the questionnaire survey was officially initiated. Ultimately, after eliminating 18 invalid questionnaires from the returned ones, a total of 611 valid questionnaires were obtained. Of these, 100 were utilized for reliability, validity, and exploratory factor analysis, while the remaining 511 were used for confirmatory factor analysis.

In the second stage, with a quasi-experimental design for the aim of probing into the actual efficacy of the flipped classroom, the research opted to select two parallel sophomore classes from a university in Shanghai, each consisting of 35 students. Prior to the commencement of the research, baseline evaluations were conducted on these two classes by means of the English learning motivation scale and English proficiency tests, thereby ensuring a high degree of comparability in terms of both motivation levels and English academic performance.Subsequently, the research team ascertained the teaching conditions for the experimental group and the control group through a rigorous random allocation procedure. To be more specific, the two classes were initially assigned numbers (for instance, Class 1 and Class 2). Thereafter, a computer-generated random number table was employed to allocate the experimental conditions, namely, flipped classroom teaching for the experimental group and traditional classroom teaching for the control group. This randomization process was overseen and implemented by independent researchers who were not directly engaged in the teaching activities. Such an arrangement was designed to guarantee the objectivity and randomness of the allocation process, effectively diminishing the likelihood of selection bias. Moreover, all the students shared the same disciplinary background, curriculum system, and teaching arrangements, which served to minimize the interference of non-experimental variables on the research outcomes.

To ensure the statistical validity of the research and the rationality of the sample size, a power analysis was conducted in this study. Using the G*Power 3.1 software, based on a large effect size (Cohen’s *d* = 0.5), with the significance level set at 0.05 and the power at 0.8, the required sample size was calculated to be 42 individuals (21 in the experimental group and 21 in the control group). The actual sample size in this study was 70 individuals (35 in the experimental group and 35 in the control group), which exceeded the theoretically required sample size, indicating the rationality of the sample size setting.

On October 7th, 2023, prior to the initiation of the research, the research team provided a detailed exposition to all participants regarding the objectives, methods, potential risks, and benefits associated with the experiment. Emphasis was placed on the fact that the experimental period would span from October 7th, 2023 to February 7th, 2024.Participants were obliged to read and sign the written informed consent forms, within which all the crucial details of the research were explicitly enumerated. These details encompassed aspects such as data confidentiality, privacy protection, and the entirely voluntary nature of participation.Throughout the course of the research, it was unequivocally communicated to the participants that they retained the freedom to withdraw from the experiment at any moment, without incurring any adverse consequences on their academic pursuits or other rights and interests.All the signed consent forms were meticulously preserved to ensure full compliance with the research procedures and to satisfy the requirements of ethical review.

### 3.2. Instruments

#### 3.2.1. English learning motivation scale.

Based on Gardner’s Attitude/Motivation Test Battery (AMTB) and Dörnyei’s Language Learning Motivation Questionnaire, this study developed an English learning motivation questionnaire within the framework of the flipped classroom and made adjustments in line with the characteristics of the research subjects. The questionnaire consisted of two parts. The first part related to the demographic information of the research subjects, and the second part contained 16 items in the form of a 5-point Likert scale (5 = strongly agree, 4 = agree, 3 = neutral, 2 = disagree, 1 = strongly disagree). Through the analyses of reliability, validity, exploratory factors, and confirmatory factors, the dimensions of the questionnaire and the items corresponding to each factor were eventually determined.

Reliability and validity are two crucial aspects in the quality assessment of the scale. According to Supporting Information (S1), the outcome of the reliability test in this study was that the Cronbach’s αcoefficient arrived at 0.935, suggesting that the entire scale hadgood reliability.

Besides reliability analysis, validity analysis is critical for assessing the structure of the questionnaire. Whether it is suitable for exploratory factor analysis depends on the value of the Kaiser Meyer Olkin measure of sampling adequacy (KMO) in the validity analysis. Generally, the closer the KMO coefficient is to 1, the more suitable the questionnaire is for factor analysis [45]. If the value is higher than 0.9, it suggests it is highly suitable. Regarding the questionnaire in this study, the KMO value was 0.901, and the Bartlett’s test of sphericity reached a significant level (*p* < 0.05). Based on these two results in S1, it is clear that this questionnaire was suitable for exploratory factor analysis.

In the exploratory factor analysis, principal component analysis was adopted and factors were extracted subsequent to varimax rotation. This study drew upon the comprehensive research outcomes [46–48], and the subsequent criteria for structural validity were embraced: the eigenvalue of each factor was required to exceed 1; the total variance attributed to the extracted factors was expected to surpass 60%. Additionally, it was indispensable that the factor loading of each item encompassed within the extracted factor be greater than 0.5. Otherwise, the item was to be excluded.

It can be perceived from Table 1 that four factors were extracted in this analysis, and the total variance expounded by these four factors amounted to 77.375%. On the whole, the results of this factor analysis were acceptable.

**Table 1:** Total variance explained.

Component	Initial Eigenvalues			Extraction Sums of Squared Loadings		
**Total**	**% of Variance**	**Cumulative %**	**Total**	**% of Variance**	**Cumulative %**
1	8.126	50.788	50.788	8.126	50.788	50.788
2	1.717	10.733	61.521	1.717	10.733	61.521
3	1.334	8.335	69.857	1.334	8.335	69.857
4	1.203	7.519	77.375	1.203	7.519	77.375

Note: Extraction method: principal component analysis.

According to the rotated component matrix presented in Table 2, these 16 items could be classified into four factors. The first factor was associated with autonomous learning and comprised 6 items: Q3, Q8, Q10, Q11, Q12, and Q13. The second was designated as instrumental motivation and incorporated 4 items: Q5, Q6, Q7, and Q9. The third was integrative motivation, involving 3 items: Q2, Q1, and Q4. The final one was named as intrinsic motivation, also consisting of 3 items: Q16, Q14, and Q15.

**Table 2:** Rotated component matrix.

	Component			
**1**	**2**	**3**	**4**
Q12 I seek learning methods appropriate for myself.	.794			
Q8 I set clear English learning goals.	.789			
Q11 I am activein the group discussion of the flipped class model.	.745			
Q3 Upon the completion of a learning task, I shall undertake self-evaluation.	.741			
Q10 I formulate a detailed English learning plan.	.724			
Q13 I encourage myselfto overcome the difficulties in English learning.	.699			
Q6 I learn English for the aim of fulfilling tasks at work place.		.868		
Q9 I learn English for the purpose of passing the English examinations at school.		.823		
Q5 By virtue of the requirements of the teacher, I study English		.793		
Q7 I study English by reason of my parents’ expectations.		.767		
Q1 To comprehend the culture of English-speaking countries, I am motivated to be proficient in English.			.877	
Q4I am eager to communicate with native English speakers to acquire an in-depth understanding of their daily lives.			.870	
Q2 I desire to travel or study abroad in an English-speaking country to experience the local culture in person.			.802	
Q16 Learning English can enhance my thinking.				.841
Q14 I learn English because I have an interest in it.				.818
Q15 I enjoy challenging myself and surmounting the difficulties in English learning.				.738

**Fig 1:**
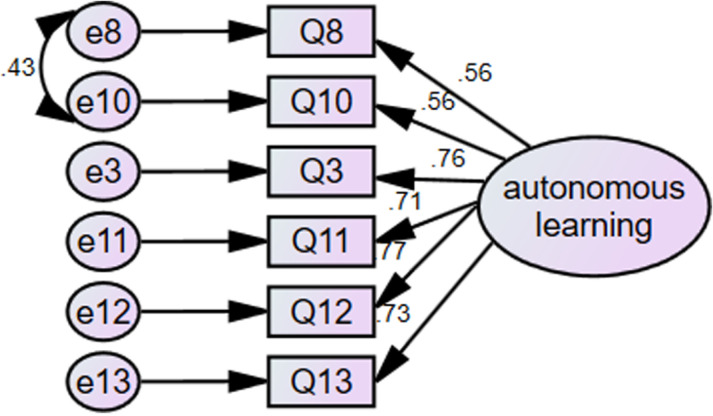
Confirmatory factor analysis for autonomous learning motivation.

**Fig 2:**
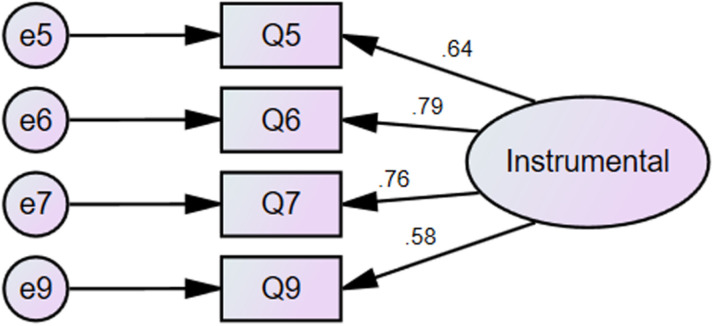
Confirmatory factor analysis for instrumental motivation in S1 Files. Additionally, all the standardized regression coefficients in these two models were higher than 0.50 and reached a significant level. This indicated that these two models were stable and reliable in terms of statistics.

After conducting reliability and validity tests and exploratory factor analysis on 100 out of the 611 questionnaires, the next step was to conduct confirmatory factor analysis on the remaining 511. The confirmatory factor analysis via AMOS was mainly split into two stages [49].In this study, the first stage was to analyze each factor to check the contribution of each observed variable to its corresponding latent variable. The second step was to test the model made up of four latent variables. That is, to see whether the model consisting of four latent variables, their respective observed variables, and error terms was supported by the data.This study employed six indicators: goodness-of-fit index, adjusted goodness-of-fit index, comparative fit index, root mean square residual, root mean square error of approximation, and CMIN/DF.Concerning the two latent variables of integrated motivation and intrinsic motivation, they were saturated in that each latent variable had only three observed variables and its goodness-of-fit index reached 1, the upper limit. Since the value of CMIN/DF in the saturated model was 0, the values of AGFI, CFI, RMSEA, and RMR couldn’t be calculated. Additionally, it was commonly held that the best fit would be achieved between the saturated model and its corresponding data [50]. Thus, there was no need to conduct a goodness-of-fit test for the saturated models of integrated motivation and intrinsic motivation (Figs 1 and 2).

On the other hand, based on the confirmatory factor analysis of the other two models--autonomous learning motivation and instrumental motivation in Table 3, CMIN/DF, GFI, AGFI, CFI, RMR and RMSEA were all within the reference range.For comprehensive insights into these two models, refer to

**Table 3:** Fit indices of autonomous learning motivation and instrumental motivation.

Index	CMIN/DF	P	GFI	AGFI	CFI	RMR	RMSEA
Reference range	≤ 5	>0.05	≥ 0.90	≥ 0.80	≥ 0.90	≤ 0.10	≤ 0.08
Autonomous learning	3.902	0.000	0.980	0.948	0.980	0.023	0.075
Instrumental	0.982	0.375	0.998	0.991	1.000	0.010	0.000

**Fig 3:**
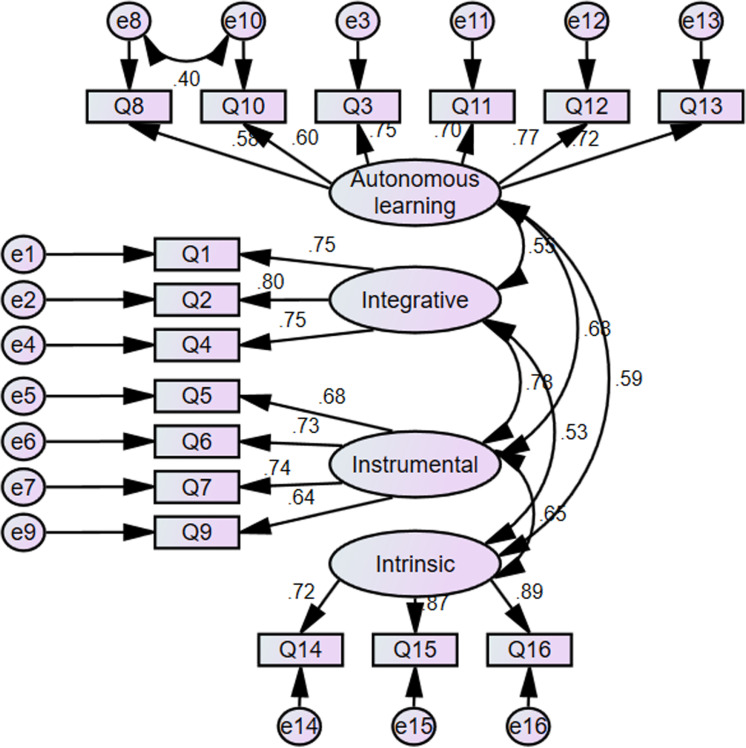
Confirmatory factor analysis of English learning motivation in the flipped class indicates that despite the relatively large sample size and a *p*-value less than 0.05, all the standardized regression coefficients presented in Table 5 were higher than 0.50. Additionally, discerned from the data in S1 the correlation coefficients among the four latent variables reached a significant level, and the regression coefficients of all observed variables to their respective latent variables also attained a significant level. Hence, the English learning motivation model in the flipped classroom composed of autonomous learning motivation, instrumental motivation, integrated motivation and intrinsic motivation was statistically stable and reliable as a whole.

The final step of confirmatory factor analysis was to examine the model consisting of all the four factors. It is apparentfrom the data in Table 4 that the fitting index of the modified model was within the reference range, and [Fig 3]. [Confirmatory factor analysis of English learning motivation in the flipped class] (detailed information can be found in S1 File)unveiled factor structures that held theoretical significance.

**Table 4:** Fit indices of English learning motivation model.

Index	CMIN/DF	P	GFI	AGFI	CFI	RMR	RMSEA
Reference Range	≤ 5	>0.05	≥ 0.90	≥ 0.80	≥ 0.90	≤ 0.10	≤ 0.08
Model Fit Summary	3.887	0.000	0.920	0.888	0.929	0.057	0.075

**Table 5:** Reliability and validity analysis.

Dimension	Measurement indicators	Item	Standardized factor loading	AVE	CR	CITC	Cronbach’sα
Autonomous learning	Self-monitoring and evaluation	Q3	0.754	0.477	0.844	0.723	0.910
	Set goals	Q8	0.583			0.796	
	Plan learning	Q10	0.596			0.803	
	Participate in English activities	Q11	0.700			0.754	
	Find methods	Q12	0.770			0.696	
	Self-encouragement	Q13	0.718			0.722	
Integrative	Interest in culture	Q1	0.754	0.589	0.811	0.817	0.909
	Sense of identity	Q2	0.802			0.806	
	Social needs	Q4	0.746			0.833	
Instrumental	Cope with exams	Q9	0.638	0.489	0.792	0.807	0.914
	Parents’ expectations	Q7	0.745			0.790	
	Teachers’requirements	Q5	0.679			0.801	
	Tasks in work place	Q6	0.731			0.816	
Intrinsic	Accept challenges	Q15	0.873	0.690	0.869	0.705	0.865
	Enhance thinking	Q16	0.888			0.749	
	Enjoy learning	Q14	0.721			0.780	

After conductingCFA, performing reliability and validity analysis is of significant theoretical and practical importance. While CFA assesses the overall model fit and provides a preliminary evaluation of the scale’s structural validity, furtherreliability and validity analysis offers a more detailed examination of the scale’s measurement quality [51]. This additional analysis strengthens the credibility of research findings and ensures the robustness of the measurement model.Reliability analysis, in particular, is essential for confirming the internal consistency of the scale. Metrics such as Composite Reliability (CR) and Average Variance Extracted (AVE) allow researchers to evaluate the consistency of latent variables and the explanatory power of their observed indicators [52]. Even when CFA fit indices indicate good model performance, inadequate reliability (e.g., CR < 0.7 or AVE < 0.5) may suggest measurement instability, warranting further investigation [53].Validity analysis, on the other hand, provides a comprehensive assessment of the scale’s effectiveness in measuring the intended constructs. Convergent validity reflects the strong correlations among the observed items of a latent variable, indicating consistency in the measurement of the construct [54]. Discriminant validity, by comparing the correlations between latent variables, ensures sufficient independence among them [55]. This step addresses the limitations of CFA, which primarily evaluates overall model fit, by offering more granular evidence of the model’s measurement quality.In summary, conducting reliability and validity analysis in AMOS after completing CFA was indispensable in the present study. This process not only reinforced the validity of the model fit but also served as a crucial step in ensuring the scale’s measurement quality, whether for academic research or practical applications.

As presented in Table 5, the AVE values for Integrative Motivation and Intrinsic Motivation exceeded 0.5, while their CR values were both above 0.7, indicating that the data demonstrated strong reliability and validity. Although the AVE values for Autonomous Learning Motivation and Instrumental Motivation were slightly below the 0.5 threshold, at 0.477 and 0.488 respectively, the results of the reliability and validity analysis on the whole still met the fundamental requirements of the measurement model. This confirmed that the questionnaire reliably and validly captured these two factors.

From an overall perspective, the CR values for Autonomous Learning Motivation and Instrumental Motivation were 0.844 and 0.792, respectively, both significantly exceeding the 0.7 benchmark. This indicated a high degree of internal consistency among the measurement items associated with these factors. Additionally, the discriminant validity analysis revealed that the square root of the AVE values for these two factors (0.691 and 0.699, respectively) were greater than their correlations with other factors in Table 6. This demonstrated that the latent variables were well-differentiated, with no significant overlap in their measurements.

**Table 6:** Discriminant validity: Pearson correlation and the square root of AVE.

.	Integrative	Instrumental	Autonomous learning	Intrinsic
Integrative	0.767			
Instrumental	0.585	0.699		
Autonomous learning	0.399	0.607	0.691	
Intrinsic	0.554	0.559	0.412	0.830

Note: The numbers on the diagonal represent the square root of the AVE.

The slightly lower AVE values may be attributable to marginally lower factor loadings for certain items or slightly higher measurement error. Nevertheless, their proximity to the 0.5 threshold, combined with the high CR values and strong discriminant validity, supported the conclusion that the overall reliability and validity of the model were satisfactory. These findings underscored the model’s robust measurement quality and its suitability for practical application.

In addition, this research conducted reliability analyses on these four factors separately anew. The results from Table 5 demonstrated that the reliability coefficients of the four dimensions, namely autonomous learning motivation, integrative motivation, instrumental motivation, and intrinsic motivation, were all above 0.7, specifically at 0.910, 0.909, 0.914, and 0.865 respectively. Furthermore, discerned from the data in Table 5, the corrected item-total correlations (CITC) for all the items incorporated within these four factors were all above 0.696. Consequently, it can be deduced that the items within each factor exhibited favorable internal consistency.

The analysis of discriminant validity presented in Table 6 indicated that the square root of the AVE for Integrative Motivation reached 0.767, which was significantly greater than the maximum absolute value of its factor correlation (0.585), demonstrating that this factor possessed strong discriminant validity. Similarly, the square root of the AVE for Instrumental Motivation was 0.699, which exceeded the maximum absolute value of its factor correlation (0.607), further confirming the adequacy of its discriminant validity. For Autonomous Learning Motivation, the square root of the AVE was 0.691, which was higher than the maximum absolute value of its factor correlation (0.607), thereby satisfying the requirements for discriminant validity. Finally, the square root of the AVE for Intrinsic Motivation was 0.830, which was considerably higher than the maximum absolute value of its factor correlation (0.559), further reinforcing the strong discriminant validity of this factor.

So far, the dimensions of the English learning motivation questionnaire and their corresponding items are presented in Table 5.

#### 3.2.2. English proficiency test.

To conduct a comprehensive assessment of students’ English proficiency and guarantee the scientificity of the testing instruments, a meticulously designed English proficiency test was utilized in both the pre-test and the post-test within this study.

To guarantee that the test was capable of yielding stable and consistent results under diverse temporal and conditional settings, this study took a series of measures to enhance the reliability of the test, which were elaborated as follows.

Firstly, the consistency of proposition was ensured. Specifically, both the pre-test and the post-test were meticulously designed by the same team of proposition experts. This was done to safeguard the uniformity of the test content with regard to knowledge coverage as well as difficulty distribution, thereby eliminating any potential disparities that might otherwise occur.Secondly, the consistency of the test form was maintained. The overall structure of the test, along with the design of various question types, remained unaltered. For illustrative purposes, in the listening section, it encompassed short conversations, long conversations, and passage comprehension. Meanwhile, in the reading section, it consisted of passage comprehension and the task of blank-filling with selected words. This consistent setup across different test administrations helped to standardize the testing experience for all participants.Finally, the standardization of scoring was implemented. The test scores were quantified as integers, which served to ensure the clarity and unwavering consistency of the scoring criteria. By doing so, it effectively mitigated the potential deviations that could potentially stem from subjective judgments during the scoring process or calculation errors, thus enhancing the reliability and objectivity of the test results.

The crux of test validity resides in ascertaining its efficacy in precisely gauging students’ English proficiency. The tests robustly ensuredtheir validity across multiple dimensions. With regard to content validity, the test content was meticulously formulated in strict adherence to the teaching syllabus and curriculum standards, comprehensively enveloping the listening, reading, vocabulary, and grammar skills that students were obligated to master. This thereby firmly secured the congruence between the test content and the learning objectives. In the realm of construct validity, the design of test task types was intricately intertwined with the actual language application scenarios. For instance, short conversations were adept at simulating daily communication circumstances, while passage reading was proficient in evaluating students’ comprehensive comprehension capabilities. This consequently ensured that the test could authentically mirror students’ English application competencies. Moreover, in terms of objective consistency, through the judicious allocation of the weights of each segment, the test results were capable of presenting a comprehensive panorama of the students’ overall English ability levels.

Based on the above considerations, two sets of test papers were ultimately generated. The full score of each test paper was 100 points, and the final score of students was rounded to an integer. The specific details of the examination are presented in Table 7 below.

**Table 7:** The makeup of the English proficiency test.

Section	Task type	Number of tasks	Number of questions	Score for each question	Question type	Total
Listening comprehension	Short Conversations	8	8	1	Multiple Choice	8
	Long Conversations	2	7	1.5	Multiple Choice	10.5
	Short Passages	3	11	1.5	Multiple Choice	16.5
Reading comprehension	Passages	3	15	2	Multiple Choice	30
	Banked Cloze	1	10	2	Blank Filling	20
Vocabulary grammar	Vocabulary	15	15	0.5	Multiple Choice	7.5
	Grammar	15	15	0.5	Multiple Choice	7.5

### 3.3. Instructional design

This research chiefly focused on examining the changes of students’ motivation levels and English proficiency in the flipped classroom model. Based on this purpose, the instructional design for the flipped classroom in this research is as follows:

In the pre-class:

- Teachers elaborately produce or select appropriate teaching videos, encompassing contents like text explanations, grammar key points, vocabulary expansion, etc., and uploaded them onto the online learning platform.
- Students independently watch the videos before class and undertake preview and initial learning.
- Teachers assign relevant preview tasks or exercises to examine students’ grasp of basic knowledge.

During the Class:

- Organize group discussions to delve into the issues from the preview or specific themes such as the analysis of a certain English cultural phenomenon.
- Conduct case analyses or situation simulations, for example, simulate business negotiation scenarios to cultivate students’practical skills in English.
- Arrange achievement display activities to enable students to share their learning experiences and viewpoints after preview.
- Teachers centrally address and explain students’ questions.
- Arrange classroom exercises and tests to consolidate the learned knowledge in a timely manner.

In the Post-class:

- Teachers offer extended resourcessuch as related English reading materials, English movies, etc., to motivate students to continue in-depth learning.
- Students communicate with teachers and classmates on the learning platform about the problems encountered during the learning process.

For instance, while teachers and students undertake the teaching tasks of one unit, students watch videos about the background knowledge and key vocabulary of the unit before class and complete simple vocabulary tests; in class, they have group discussions on the theme and writing skills of the unit, and then conduct role-play to showcase the plot in the article; after class, students watch the English speech video of the same theme recommended by the teacher.

### 3.4. Research process

This research conducted a four-month teaching experiment from the end of February to the end of June 2024. During this period, the experimental class adopted the flipped classroom teaching approach, while the control class still employed the traditional grammar translation teaching method. The two classes used the same English instruction materials, and both the teaching duration and arrangement remained consistent.

Before the teaching experimenttwo pre-tests were carried out with the assistance of the English learning motivation questionnaire and the pre-test of English proficiency for three reasons. The first was to understand whether there were significant differences in learning motivation between the two classes. The second was to verify whether there were significant disparities in English grades between the two classes. The last was to offer a reference for the comparison of the data obtained after the subsequent teaching experiment. After the teaching experiment concluded, a post-test was conducted through the same motivation questionnaire and the post-test of English proficiency, and these results were compared with the pre-test results.

### 3.5. Data collection and analysis

Data were collected by the English learning motivation questionnaire and two English proficiency tests. These data were analyzed as follows with the aid of SPSS 27.0.

Regarding the exploration of whether the flipped classroom teaching could significantly enhance students’ English learning motivation, two independent sample *t*-tests were conducted to compare the data obtained from the same motivation scale of the two classes before and after the teaching experiment. Meanwhile, paired sample *t*-tests were employed to respectively investigate whether there were significant changes in each class before and after the experiment.

To explore the impact of the flipped classroom teaching on English grades, two independent sample *t*-tests wereconducted again by using SPSS 27.0 to compare the English grades before and after the experiment. In addition, paired sample *t*-tests wereimplemented to explore whether there were significant changes in each class before and after the experiment respectively.

As for the investigation of the influence of motivation on English grades, the data related to motivation and English grades of both classes in the post-test were input into SPSS 27.0 for correlation and multiple regression analysis.

## 4. Results

### 4.1. The impact of flipped classroom teaching on English learning motivation

In order to explore the levels of English learning motivation of students in the experimental class and the control class before the experiment, this study performed an independent sample *t*-test on the data of the two classes from the first survey related to motivation. The results are presented in Table 8.

**Table 8:** Differences in the pretest scores of English learning motivation between the experimental class and the control class.

Variable	Class	Sample size	Mean	SD	*t*	*P*	Cohen’s *d*
Integrative	Experimental	35	2.86	0.71	-0.386	0.700	-0.092
	Control	35	2.92	0.73			
Instrumental	Experimental	35	2.63	0.99	0.000	1.000	0.000
	Control	35	2.63	0.70			
Autonomous learning	Experimental	35	2.83	1.12	0.462	0.646	0.110
	Control	35	2.72	0.84			
Intrinsic	Experimental	35	2.75	0.88	-0.809	0.422	-0.193
	Control	35	2.93	0.99			
Total	Experimental	35	2.77	0.68	-0.229	0.819	-0.055
	Control	35	2.80	0.58			

As presented in Table 8, the means and SD of the experimental class and the control class in the overall score and four dimensions of English learning motivation were relatively proximate (the mean of the overall motivation: 2.77 with a SD of 0.68 in the experimental class; 2.80 with a SD of 0.58 in the control class). The *t*-test result indicated that the difference was not statistically significant (*P* > 0.05). Notably, the Cohen’s *d* values were all in a relatively low range (all less than 0.2), which implied that the practical effect of the difference between the groups was minimal, thereby suggesting that the motivation levels of the two classes were essentially equivalent at the outset of the experiment, validating the fundamental conditions of the experimental design.Nevertheless, subsequent to the teaching experiment, this study once again employedan independent sample t-*t*est to investigate the influence of flipped classroom-based English teaching on students’ English learning motivation. As illustrated in Table 9, flipped classroom teaching markedly enhanced the performance of students in the experimental class across all dimensions and the total score of English learning motivation. For instance, with regard to the overall motivation, the mean of the experimental class surged from 2.77 to 3.58 while the SD marginally increased from 0.68 to 0.69, in contrast to the relatively minor fluctuation in the control class ranging from 2.80 to 2.86, with the SD varying from 0.58 to 0.64. The t-*t*est result revealed a highly significant difference in the overall motivation level between the experimental class and the control class (t = 4.477, *P* < 0.001), and *t*he effect size (Cohen’s *d* = 1.070) was relatively large, indicating a substantial effect. Specifically, at the dimension levelsuch as autonomous learning motivation, the mean of the experimental class experienced a significant elevation from 2.83 in the pre-test to 3.84 in the post-test, and the effect size (*d* = 1.331) was notably large, vividly reflecting the pronounced impact of the teaching method in this particular dimension.

**Table 9:** Differences in the post-test scores of English learning motivation between the experimental class and the control class.

Variable	Class	Sample size	Mean	SD	*t*	*P*	Cohen’s *d*
Integrative	Experimental	35	3.53	0.90	2.651	0.010^*^	0.634
	Control	35	2.97	0.87			
Instrumental	Experimental	35	3.34	0.91	2.881	0.005^**^	0.689
	Control	35	2.72	0.89			
Autonomous learning	Experimental	35	3.84	0.78	5.569	0.000^**^	1.331
	Control	35	2.80	0.79			
Intrinsic	Experimental	35	3.58	0.94	2.889	0.005^**^	0.691
	Control	35	2.95	0.88			
Total	Experimental	35	3.58	0.69	4.477	0.000^**^	1.070
	Control	35	2.86	0.64			

Note:

* *P* < 0.05

** *P* < 0.01.

In addition, the findings of the paired-samples *t*-test further substantiated the efficacy of the flipped classroom teaching approach. As presented in Table 10, the discrepancies between the pre-test and post-test of the English learning motivation within the control groupin the overall and all dimensions failed to reach statistical significance (*P* > 0.05). In contrast, the experimental group, as depicted in Table 11, exhibited a pronounced and statistically significant enhancement in all dimensions and the overall score (*P* < 0.001). Notably, with respect to the integrative motivation, the mean value in the experimental class ascended from 2.86 to 3.53, accompanied by a relatively large effect size of *d* = 0.759. This evidently demonstrated that the flipped classroom teaching exerted a substantial and positive impact on students’ intrinsic and extrinsic motivations, with the effect size serving as a clear indicator of the magnitude of this influence.

**Table 10:** Differences in English learning motivation between the pre- and the post-test scores for the control class.

Variable	Test Type	Sample size	Mean	SD	*t*	*P*	Cohen’s *d*
Integrative	Pre-test	35	2.92	0.73	-0.290	0.773	-0.049
	Post-test	35	2.97	0.87			
Instrumental	Pre-test	35	2.63	0.70	-0.639	0.527	-0.108
	Post-test	35	2.72	0.89			
Autonomous learning	Pre-test	35	2.72	0.84	-0.651	0.519	-0.110
	Post-test	35	2.80	0.79			
Intrinsic	Pre-test	35	2.93	0.99	-0.100	0.921	-0.017
	Post-test	35	2.95	0.88			
Total	Pre-test	35	2.80	0.58	-0.938	0.355	-0.159
	Post-test	35	2.86	0.64			

**Table 11:** Differences in the scores of English learning motivation between the pre-test and the post-test for the experimental class.

Variable	Test Type	Sample size	Mean	SD	*t*	*P*	Cohen’s *d*
Integrative	Pre-test	35	2.86	0.71	-4.489	0.000^**^	-0.759
	Post-test	35	3.53	0.90			
Instrumental	Pre-test	35	2.63	0.99	-4.264	0.000^**^	-0.721
	Post-test	35	3.34	0.91			
Autonomous learning	Pre-test	35	2.83	1.12	-5.550	0.000^**^	-0.938
	Post-test	35	3.84	0.78			
Intrinsic	Pre-test	35	2.75	0.88	-4.605	0.000^**^	-0.778
	Post-test	35	3.58	0.94			
Total	Pre-test	35	2.77	0.68	-7.408	0.000^**^	-1.252
	Post-test	35	3.58	0.69			

Note:

** *P* < 0.01.

### 4.2. The impact of flipped classroom instruction on English learning performance

This study performed an independent sample *t*-test on English proficiency scores of the experimental class and the control class in the pretest to investigate whether significant differences existed before the experiment. The results are presented in Table 12.

Table 12 exhibits that the means of the English proficiency levels of the experimental class and the control class prior to the experiment were proximate with the experimental class registering at 61.20 and the control class at 62.23. TheirSD were relatively elevated (7.84 for the experimental class and 9.85 for the control class). The *t*-test result failed to reveal a significant difference (*P* > 0.05), and the effect size *d*-value was -0.116. This implied that the starting levels of the two classes were comparable, thereby validating the fairness and rationality of the experimental design.Upon the completion of the experiment, the independent sample *t*-*t*est was once again conducted on the post-experiment test scores of both classes to investigate whether a significant disparity existed in their English proficiency levels. As discernible from Table 13, the mean score of the students in the experimental class in the post-experiment test was clearly higher than that of the control class (68.06 for the experimental class and 61.71 for the control class), and the difference was statistically significant (*t* = 3.336, *P* < 0.01). No*t*ably, the effect size *d*-value attained 0.797, which indicated that the flipped classroom teaching had a substantial and meaningful practical impact on the enhancement of English scores with the magnitude of the effect size highlighting the significant degree of improvement.

**Table 12:** Differences in pre-test scores of English between the experimental class and the control class.

	Class	Sample size	Mean	SD	*t*	*P*	Cohen’s *d*
Pre-test	Experimental	35	61.20	7.84	-0.483	0.630	-0.116
	Control	35	62.23	9.85			

**Table 13:** Differences in post-test scores of English between the experimental class and the control class.

	Class	Sample size	Mean	SD	*t*	*P*	Cohen’s *d*
Post-test	Experimental	35	68.06	8.00	3.336	0.001^**^	0.797
	Control	35	61.71	7.91			

Note:

** *P* < 0.01

In addition to the independent-samples *t*-test, this study further carried out paired-samples *t*-tests on the English test scores of both the control group and the experimental group respectively. Tables 14 and 15 illustrate the fluctuations in the English scores of the two groups of students before and after the experiment. The scores of the control group did not exhibit a significant difference between the pre-test and the post-test (*P* > 0.05), with a relatively small effect size of *d* = 0.056, indicating a negligible change. In contrast, the scores of the experimental group showed a significant increase (*P* < 0.001), and the effect size *d* reached a relatively large value of 0.707. This more evidently validated the substantial and notable effectiveness of the flipped classroom teaching approach in enhancing English scores, with the magnitude of the effect size highlighting the degree of improvement and emphasizing the practical significance of this teaching method in promoting academic performance.

**Table 14:** Differences in the scores of English proficiency between the pre-test and the post-test for the control class.

Test	Sample size	Mean	SD	*t*	*P*	Cohen’s *d*
Pre-test	35	62.23	9.85	0.331	0.743	0.056
Post-test	35	61.71	7.91			

**Table 15:** Differences in the scores of English proficiency between the pre-test and the post-test for the experimental class.

Test	Sample size	Mean	SD	*t*	*P*	Cohen’s *d*
Pre-test	35	61.20	7.84	-4.184	0.000^**^	-0.707
Post-test	35	68.06	8.00			

Note:

** *P < *0.01

### 4.3. The predictive power of motivational factors on English proficiency

To investigate whether English proficiency could be predicted by integrative motivation, instrumental motivation, autonomous learning motivation, and intrinsic motivation, this study first conducted a correlation analysis between these four factors and English grades in the post-test, and then carried out a multiple linear regression analysis.

The Pearson coefficient was employed to indicate the strength of the correlation. The data in Table 16 reveals that English scores were significantly positively correlated with integrative motivation, instrumental motivation, autonomous learning motivation, and intrinsic motivation, with the correlation coefficients at 0.349, 0.394, 0.457, and 0.547 respectively. Furthermore, there existed a significant positive correlation among these four factors of English learning motivation.

**Table 16:** Correlation analyses between the variables in English learning motivation and posttest scores of two classes.

.	Test score	Integrative	Instrumental	Autonomous learning	Intrinsic
Test score	1				
Integrative	0.349^**^	1			
Instrumental	0.394^**^	0.585^**^	1		
Autonomous learning	0.457^**^	0.399^**^	0.607^**^	1	
Intrinsic	0.547^**^	0.554^**^	0.559^**^	0.412^**^	1

Note:

** *P* < 0.01

When conducting the linear regression analysis, the figures in the post-test for integrative motivation, instrumental motivation, autonomous learning motivation, and intrinsic motivation were regarded as independent variables, while the post-test English scores of the experimental class and the control class were taken as dependent variables. Based on the data in Table 17, the “entry” regression method indicates that the combination of the four independent variables exerted a significant predictive effect on students’ English proficiency, with F (4, 65) = 9.323, *P* = 0.000 < 0.05. Among them, autonomous learning motivation (*P* = 0.022 < 0.05) and intrinsic motivation (*P* = 0.001 < 0.01) made significant contributions to the prediction. However, the regression coefficients of integrative motivation and instrumental motivation did not reach the statistically significant level. Regarding collinearity, the values in terms of the variance inflation factor (VIF) for all four independent variables were all less than 5, signifying the absence of multicollinearity.

**Table 17:** Summary of multiple linear regressionsof motivational factors on English test score.

	Unstandardized Coefficients		Standardized Coefficients	*t*	*P*	Collinearity Statistics	
*B*	Std. Error	*Beta*		VIF	Tolerance	
Constant	43.970	3.784	–	11.621	0.000**	–	–
Integrative	0.075	1.194	0.008	0.063	0.950	1.718	0.582
Instrumental	-0.317	1.313	-0.035	-0.242	0.810	2.189	0.457
Autonomous learning	2.654	1.132	0.294	2.346	0.022*	1.604	0.623
Intrinsic	3.926	1.132	0.441	3.467	0.001**	1.657	0.604
*R* ^2^	0.365						
Adjusted *R* ^2^	0.325						
*F*	*F* (4,65)=9.323, *P* = 0.000						
Dependent Variable: English test score							

## 5. Discussion

### 5.1. The positive impact of the flipped classroom on English learning motivation (RQ1)

College English teaching based on the flipped classroom can effectively heighten students’motivation level. Here are the reasons from the following four perspectives.

Firstly, the flipped classroom can enhance students’ integrative motivation. Before class, teachers upload elaborately designed learning materials to the online learning platform. In the teaching videos, documents and other materials, rich English cultural elementssuch as interesting customs and cultural stories of English-speaking countriesare incorporated, thereby arousing students’ curiosity. In class, through timely feedback, teachers offer encouragement for students’efforts and achievements in English culture learning, boosting their self-confidence and sense of achievement [56]. After class, teachers expand students’ learning resources. For instance, on the online teaching platform, high-quality resources related to English culture, like e-books, movies and websitesare recommended, facilitating students’ further exploration.

In terms of instrumental motivation, the flipped classroom model accords more flexible arrangements of learning time and space. Students are empowered to undertake independent preview beyond the classroom in accordance with their own demands. Consequently, they can plan the learning progress more optimally. Alsothey can formulate more individualized learning plans for instrumental goals such as passing examinations, obtaining certificates, and enhancing career competitiveness [57]. Secondly, the augmented interaction within the classroom is conducive to the direct application of the acquired knowledge by students. For instance, by simulating scenarios such as business negotiations, study abroad interviewsand academic exchanges, students can truly perceive the role of English in practical instrumental circumstances, and subsequently clarify the significance of learning English for achieving their own instrumental goals.

Besides, the flipped classroom fosters an environment that is highly conducive to stimulating students’ autonomous learning motivation through multiple means. It urges students to pose questions in class and tackle them by means of cooperative learning, inquiry-based learning, and other approaches. This process of resolving practical matters enables students to comprehend their own learning value and thereby reinforces their willingness to actively explore and learn. When students successfully acquire knowledge through autonomous learning and can proficiently apply and demonstrate it in class, their sense of self-efficacy will be markedly enhanced [58]. This will impel them to engage more actively in subsequent learning.

The flipped classroom can also exert a positive influence on the intrinsic motivation of English learning in the following aspects [59]. Firstly, it stimulates autonomous exploration: Within the flipped classroom, students are required to independently study the course content before the class. This process of autonomous exploration empowers students to deeply investigate English in accordance with their own pace and interests. When students can independently determine the depth and direction of learning, it is more likely for them to develop interest and enthusiasm for learning. Secondly, it cultivates the ability to solve problems. Students encounter problems during self-study before class and subsequently solve them through interaction with teachers and classmates in class. This process of overcoming difficulties through their own efforts enables students to experience a sense of achievement and stimulates their desire for further exploration and learning.

The research finding indicating that college English teaching based on the flipped classroom can effectively boost students’ motivation levels holds profound significance for lifelong learning.On the one hand, it serves to foster the development of students’ active learning attitudes. Once students’ motivation levels are elevated, they will transition from passively receiving knowledge to actively seeking it [60]. In traditional classrooms, students tend to rely heavily on teachers’ lectures. By contrast, within the flipped classroom environment, the enhanced motivation impels them to actively explore pre-class learning resources and engage in independent thinking regarding various problems. They will perceive learning as a process of self-improvement and interest fulfillment, which exerts a positive influence on the evolution of their lifelong learning capabilities, thereby enabling them to maintain an active posture when confronted with diverse challenges in the future [61].On the other hand, this research outcome can augment the continuity and perseverance of students’ learning. During the long-term process of English learning, numerous difficulties are bound to be encountered, such as complex grammar, arduous vocabulary memorization, and unfluent oral expression. The elevation of motivation endows students with greater determination and tenacity to tackle these challenges. They will not readily abandon their learning efforts merely due to the absence of immediate progress. Instead, they will consistently invest time and energy to maintain the coherence of their learning process, thereby gradually enhancing their comprehensive abilities.

### 5.2. The positive influence of motivational factors on English scores (RQ2)

Through conducting exploratory factor analysis and confirmatory factor analysis, this study extracted four motivational factors: integrative motivation, instrumental motivation, autonomous learning motivation, and intrinsic motivation. All of these four motivational factors bore a significant positive correlation with English scores. In the multiple regression analysis, autonomous learning motivation and intrinsic motivation could significantly predict English proficiency.

The positive impact of autonomous learning motivation on English scores is mainly demonstrated in the following aspects. Firstly, students with a strong autonomous learning motivation in English tend to actively set definite goals, which can provide clear guidance for their learning, make the learning more targeted, and thereby contribute to enhancing the learning efficiency and scores. Secondly, such students usually tend to actively search for appropriate learning strategies. They might try various learning approachessuch as reading English materials and watching English moviesto enhance their abilities of listening, speaking, reading and writing. Furthermore, students with a strong autonomous learning motivation have better self-supervision and self-management abilities [62]. They can consciously arrange the learning time, overcome procrastination and other undesirable habits, and maintain the consistency of learning. This self-discipline can ensure that they fully utilize learning resources, continuously accumulate knowledge and skills, and thereby obtain better scores in the examinations.

The intrinsic motivation in English usually has a positive and significant impact on English scores, which is mainly manifested in the following aspects. Increased learning investment: Students with a strong intrinsic motivation tend to dedicate considerable time and energy to the acquisition of English. They not only are satisfied with fulfilling the tasks assigned by teachers, but also independently seek more learning opportunitiessuch as reading extra-curricular English books and watching English movies, thereby accumulating more language knowledge and enhancing skills [63]. Cultivate perseverance: When facing difficulties and setbacks in learning, intrinsic motivation can give students the strength to persist. They will not easily give up just because they do not see progress in the short term. Instead, they will continue to work hard and constantly adjust their learning methods until they achieve satisfactory results.

This finding has yieldedsignificant implications for English teaching practice.Firstly, from the perspective of teaching practice, the recognition of the remarkable correlations between these four motivational factors and English academic achievements indicates that the sources of students’ motivation in English learning are multidimensional. This serves as a reminder to educators that they should not adopt a one-sided view when considering students’ learning motivation; rather, they ought to be aware that each student is typically influenced by a confluence of multiple factors. For instance, some students might develop an integrative motivation on account of their passion for English culture, thereby sparking an interest in English learning. Simultaneously, they may also aspire to attain good grades in English examinations, which exemplifies the existence of an instrumental motivation [64]. During the learning process, they are capable of relishing the joy of independently exploring knowledge, which epitomizes the autonomous learning motivation. Moreover, they can derive a sense of accomplishment from the English learning experience itself, which pertains to the intrinsic motivation [65]. Consequently, educators are required to attend to the complexity of students’ learning motivation to better customize their instructional approaches.Furthermore, the significant predictive capabilities of autonomous learning motivation and intrinsic motivation with respect to English proficiency, as disclosed by the multiple regression analysis, suggest that these two forms of motivation play a more pivotal role in students’ English learning outcomes. The implication for teaching practice is that cultivating students’ autonomous learning capabilities and stimulating their intrinsic learning interests should be regarded as crucial objectives of teaching. When students are equipped with the motivation for autonomous learning and intrinsic learning, they are more likely to achieve outstanding grades in English learning, and such capabilities and motivation will exert a positive influence on their lifelong learning [66].

In light of the aforementioned circumstances, the following present several targeted teaching strategy recommendations for educators.Concerning the cultivation of autonomous learning motivation, emphasis should be placed on developing students’ capacity to formulate learning plans. Educators are expected to guide students in devising rational learning plans by taking into account their respective English proficiency levels, learning objectives, and time schedules [67]. Moreover, students ought to be directed to dissect long-term goals into short-term, executable sub-goals, with specific learning tasks and time milestones clearly defined for each. For instance, for those students aiming to enhance their English vocabulary, educators could assist them in formulating a plan to memorize a set number of words on a daily basis, while also offering guidance on effective word-memorization methods and techniques. Simultaneously, regular inspections of the execution status of students’ learning plans should be conducted, and students should be encouraged tomake adjustments and optimizations in accordance with the actual situation [68]. Through such means, students’ self-management and planning capabilities can be nurtured, thereby stimulating their autonomous learning motivation.

With regard to targeting intrinsic motivation, initially, the design of engaging teaching activities is of paramount importance [69]. Educators should integrate interesting elements into the teaching process and contrive creative and challenging teaching activities to arouse students’ intrinsic learning interests [70]. For example, English story creation competitions could be organized, enabling students to unleash their imagination and compose stories in English. Additionally, activities like English word chain games and English riddle guessing could also be arranged to inject an element of fun into the learning experience. In the context of English reading instruction, interesting novels, comics, and the like could be selected as reading materials to entice students to engage in active reading. By means of these engaging teaching activities, students can immerse themselves in English learning within a relaxed and pleasant atmosphere, experience the joy of learning, and consequently, stimulate their intrinsic motivation.Subsequently, students should be encouraged to undertake independent exploration and innovation. Educators are required to provide open-ended learning tasks, prompting students to independently explore problems, seek solutions, and develop innovative approaches [71]. For example, in English grammar teaching, a number of error cases occurring in actual language usage could be presented for students to discuss in groups, analyze the causes of errors, and propose corrective measures. In English writing teaching, topic essays without fixed answers could be assigned to encourage students to think and express their views from diverse perspectives, thereby cultivating their innovative thinking. During this process, educators should offer ample support and guidance to students, affirm and encourage their innovative ideas and efforts, enabling students to attain a sense of achievement in the course of independent learning and innovation, and thereby strengthening their intrinsic motivation.

In summary, by implementing the above-targeted teaching strategies for different motivational factors, educators can more effectively meet the diverse learning needs of students, kindle their learning motivation, enhance the quality of English teaching, foster students’ achievement of better results in English learning, and facilitate their comprehensive development.

### 5.3. The positive impact of flipped classroom on English scores (RQ3)

Based on the above discussion, the flipped classroom is capable of enhancing the levels of autonomous learning motivation and intrinsic motivation. Moreover, autonomous learning motivation and intrinsic motivation can significantly predict English scores. Hence, the flipped classroom can elevate students’ English proficiency.

Firstly, the flipped classroom exerts a positive influence on English proficiency through autonomous learning motivation. Since the flipped classroom can stimulate autonomous learning motivation, it thereby enhances students’ capacity for independently solving problems [72]. If students confront problems during the process of autonomous learning, they will pursue answers and solve problems through their own endeavors. This not only elevates their ability of independent thinking but also deepens their comprehension of English knowledge. For example, when encountering unfamiliar words, students can understand their meanings by consulting dictionaries or guessing in the light of the context, and the memory will be more consolidated. Additionally, the flipped classroom also provides an opportunity for individualized learning. Given that each student’s English foundation and learning pace differ, the flipped classroom allows them to regulate the learning progress in accordance with their own conditions. So, students with a solid English foundation can swiftly browse the basic knowledge and focus more on the expanded content while students with a relatively weak foundation can slow down the pace and repeatedly study the key parts.

This personalized learning has a close and positive connection with the improvement of English grades. Firstly, it can fulfill the unique needs and learning styles of students, thereby enhancing grades more effectively. Students have diverse advantages and disadvantages in English learning. Some have outstanding listening skills but poor writing skills, while others excel in grammar but are deficient in vocabulary. Personalized learning addresses these differences and enables students with autonomous learning motivation to formulate plans and select strategies on their own [73]. Secondly, it can fully stimulate students’ learning interest and enthusiasm. When the learning content and methods are in line with students’ personalities, they will be more actively engaged and thereby improve their English proficiency. For instance, students who like music can learn vocabulary and grammar through English songs, and students who love movies can enhance their listening and comprehension skills by watching original soundtrack films. Additionally, during the course of planning the learning process and choosing learning resources based on their own circumstances, students gradually acquire the skills of self-management and supervision, which is of crucial importance for long-term English learning. In conclusion, personalized learning provides robust support for the improvement of English grades from the aspects of stimulating interest and strengthening self-management.

Likewise, intrinsic motivation exerts a distinctive and significant role in elevating English academic performance within the framework of the flipped classroom model. Firstly, it facilitates in-depth classroom participation: flipped classroom model cherishes interactions and students endowed with intrinsic motivation for English learning are more disposed to actively involve themselves in group discussions, express their individual viewpoints, and conduct brainstorming with teachers and classmates [74]. Such profound participation assists them in grasping complex knowledge points more proficiently, expanding their thinking and enhancing their language skills.

Given that the flipped classroom has been demonstrated to enhance students’ English proficiency, it follows that educators are obliged to reevaluate the deficiencies of the traditional teaching model and actively incorporate the flipped classroom model into their daily instructional practices. This compels schools and teachers to allocate teaching resources in a more rational manner. Specifically, more efforts should be dedicated to the creation of high-quality pre-class learning resources, including teaching videos, online tests, and supplementary materials, so as to ensure that classroom time is utilized for more efficient interactions, such as answering students’ questions and deepening their knowledge [75].For example, teachers can allocate a portion of the time that was previously used for imparting basic knowledge in the classroom to students’ pre-class autonomous learning. Subsequently, during the in-class sessions, they can concentrate on resolving the issues that students encounter during their self-study process and organize activities like group discussions and role-playing, thereby augmenting the efficiency of teaching resource utilization.

The effective implementation of the flipped classroom necessitates that teachers transform from being traditional knowledge disseminators to learning guides, organizers, and facilitators [76]. Teachers are required to possess enhanced instructional design capabilities, classroom management capabilities, and information technology application capabilities. For instance, they should master the art of designing appealing pre-class learning tasks to prompt students to actively engage in classroom interactions and utilize online teaching platforms for teaching management and evaluation.This, in turn, spurs teachers to continuously upgrade their professional competencies by participating in relevant trainings and studies, communicating with their peers, and exploring innovative teaching methods and strategies to meet the demands of flipped classroom teaching and achieve their own professional development.

## 6. Conclusions and implications

Following a four-month college English teaching experiment based on the flipped classroom and through the analysis and comparison of the data of the experimental class and the control class before and after the experiment, the following conclusions were made.

Firstly, the flipped classroom model could effectively stimulate students’ English learning motivation. At the end of the experiment, significant differences were detected between the experimental class and the control class. Compared with the control class, the motivation level of students in the experimental class was significantly enhanced. After conducting further statistical analysis of the experimental class, it was found that the overall level of motivation and the scores in the four aspects of integrative motivation, instrumental motivation, autonomous learning motivation, and intrinsic motivation had all increased markedly.Secondly, English teaching based on the flipped classroom could significantly improve students’ English proficiency. After the conclusion of the experiment, the post-test scores of the experimental class increased significantly. This indicates that the flipped classroom teaching model played a genuinely effective role in enhancing the English proficiency of college students.Thirdly, there was a significant positive correlation between English test scores and English learning motivation factors, among which autonomous learning motivation and intrinsic motivation exerted significant predictive influences on the scores.

In the research on the flipped classroom related to college students’ English learning motivation, this study comes up with a series of suggestions, aiming to furnish references for teachers, administrators and policy makers. Firstly, in the flipped classroom model, emphasis might be placed on the cultivation of autonomous learning motivation by teachers. In terms of providing personalized learning, tailored learning plans and task lists can be formulated for students by teachers in accordance with their English proficiency and learning characteristics. Meanwhile, appropriate English learning resourcessuch as specific learning websites, apps or English reading materials, can be recommended for various kinds of students. Besides the autonomous learning motivation of English, another aspect that merits priority attention is the intrinsic learning motivation. With respect to stimulating curiosity, interesting English learning tasks closely related to students’ lives like making family travel plans in English can be devised by teachers. In terms of establishing goals and a sense of achievement, clear, feasible and challenging personal English learning goals can be set for students through assistance. A case in point is memorizing a certain number of words or proficiently mastering a grammar structure within a month. In the classroom, students’learning achievements can also be presented on a regular basis via the means like English speech contests, enabling them to experience the joy of success.

This study’s broader relevance to education can be observed in several areas. First, the study enriches the practical application of motivation theory through an in-depth exploration of integrative motivation, instrumental motivation, autonomous learning motivation, and intrinsic motivation. Future research could build on these findings to further refine the ways in which different types of motivation manifest within various teaching models, explore the interactions between motivational factors, and examine their combined effects on learning outcomes, thereby advancing motivation theory. Furthermore, the study underscores the potential of the flipped classroom in supporting personalized learning, offering valuable directions for research and development in educational technology. Future studies might focus on applying artificial intelligence, learning analytics, and adaptive learning technologies to support students’ self-directed learning and personalized development more effectively, promoting deeper integration of technology in education.

In conclusion, the theoretical contributions and practical implications of this study extend beyond the domain of college English teaching. They provide important insights for advancing motivation theory and integrating technology in education, contributing to innovation and progress in the broader educational landscape.

## 7. Limitations and suggestions for future research

In this study, despite exploring the influence of the flipped classroom on college students’ English learning motivation through systematic research design and rigorous data analysis methods, certain limitations remain.

Firstly, the sample size is relatively small, possibly affecting the universality of the research results. The samples were mainly from universities in Shanghai, China, being of a small scale and lacking cross-regional and diversified sources. Hence, to enhance the universality of the conclusions, future studies might expand the sample range and incorporate more universities and students from different regions.

Secondly, in the course of this research, potential confounding variables such as students’ prior English proficiency and socioeconomic background were not comprehensively incorporated into the scope of consideration. Indeed, these factors are highly likely to exert a relatively significant influence on students’ performance within the flipped classroom model [77]. Specifically, with regard to the factor of prior English proficiency, students with a relatively higher level of English tend to possess a greater capacity to adapt to the self-directed learning requirements of the flipped classroom, thereby potentially demonstrating a more prominent enhancement in learning motivation [78].When it comes to the aspect of socioeconomic background, the family’s socioeconomic circumstances may impact students’ ability to access additional learning resources. It should be noted that the effective implementation of the flipped classroom hinges to a large extent on online learning platforms. However, for those students from families with less favorable economic conditions, they may fail to obtain high-quality electronic devices like computers and tablets, nor can they enjoy stable Internet access. These situations will inevitably impose limitations on the effectiveness of their pre-class self-directed learning.Based on this, future related research could collect students’ language learning experiences (encompassing training experiences, international exchange experiences, etc.) and family background information (including parents’ education levels, family incomes, etc.), and introduce relevant control variables in the data analysis phase, so as to dissect the actual application effects of the flipped classroom more clearly and accurately.

Thirdly, this study relied mainly on the questionnaire survey for data collection. Future studies can consider employing other methods like interviews and classroom observations to obtain more comprehensive data [79]. Additionally, long-term longitudinal studies can provide deeper insights into the long-term impact of flipped class method. Moreover, this study mainly adopted quantitative analysis methods, which, though presenting some data trends, pay less attention to the complexity of individual differences and specific contexts. Future studies can combine qualitative analysis methods like case studies to deeply explore students’ specific learning experiences and motivation variation mechanisms, thereby providing richer and more detailed explanations [80]. Finally, this study focused mainly on the impact of the flipped classroom on college students’ English learning motivation rather than comprehensively explored its influence on other learning outcomes likelearning strategies, and critical thinking ability. Future studies can broaden the perspective and comprehensively examine the overall impact on students’quality, providing more comprehensive theoretical support and empirical basis for educational practice.

In conclusion, even though this study has acquired some valuable discoveries in exploring the influence of the flipped classroom on college students’ English learning motivation and English proficiencies, there remain limitations in aspects such as sample selection, inadequacy in considering confounding variables, data collection methods, data analysis, and research perspectives. Future studies need to make improvements and expansions in these areas, hoping to obtain more comprehensive and reliable research conclusions and provide a firmer theoretical foundation and practical guidance for the application of the flipped classroom in English teaching.

## Supporting information

S1 File Supporting information for all tables and figures. (ZIP)
