# Supplementary material for: The impact of the flipped classroom on the motivation and academic performance of Chinese college English learners
Source: PLoS One. 2025 May 2;20(5):e0322094. doi: 10.1371/journal.pone.0322094 (PMC12047774; doi:10.1371/journal.pone.0322094)
Supplement: S1 File — (ZIP) [file pone.0322094.s001.zip › S1/Confirmatory Factor Analysis of English Learning Motivation Model.docx]

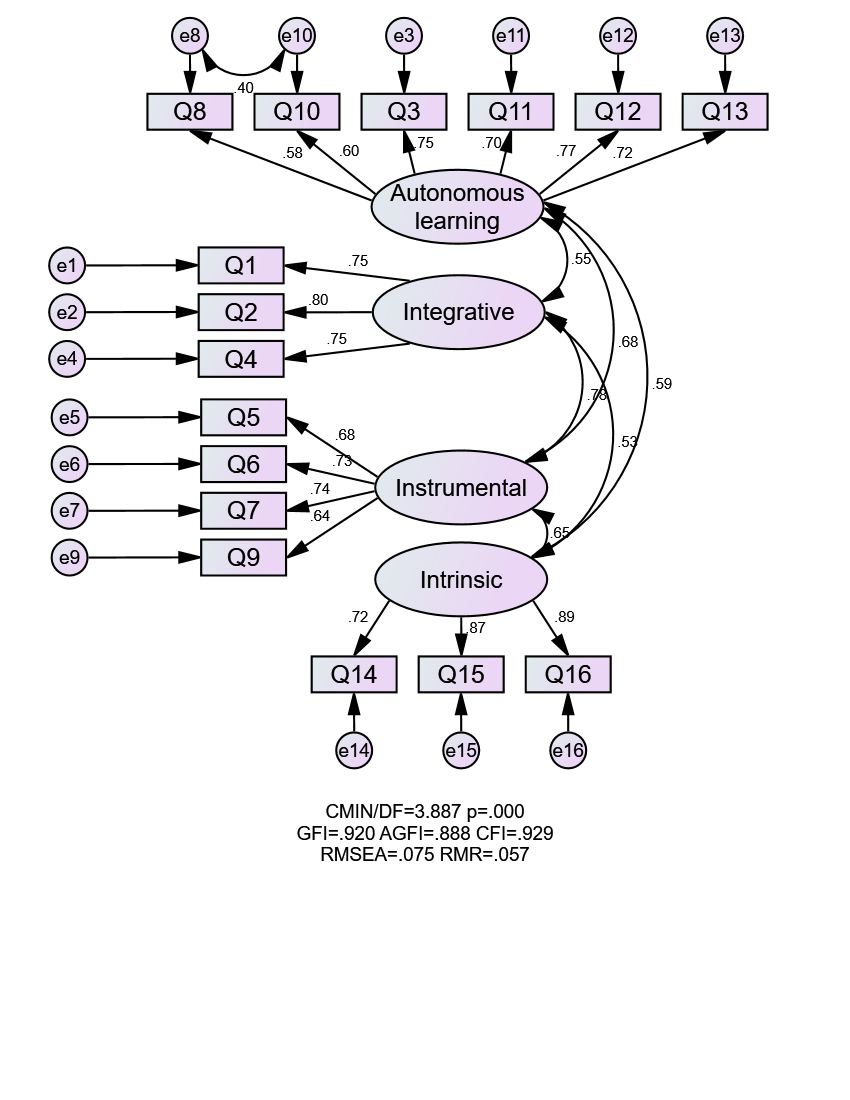


**Estimates (Group number 1 - Default model)**

**Scalar Estimates (Group number 1 - Default model)**

**Maximum Likelihood Estimates**

**Regression Weights: (Group number 1 - Default model)**

|  |  |  | Estimate | S.E. | C.R. | P | Label |
| --- | --- | --- | --- | --- | --- | --- | --- |
| Q8 | <--- | Autonomous_learning | 1.000 |  |  |  |  |
| Q10 | <--- | Autonomous_learning | .952 | .069 | 13.792 | *** | par_1 |
| Q3 | <--- | Autonomous_learning | 1.147 | .092 | 12.456 | *** | par_2 |
| Q11 | <--- | Autonomous_learning | 1.063 | .089 | 11.909 | *** | par_3 |
| Q12 | <--- | Autonomous_learning | 1.266 | .100 | 12.609 | *** | par_4 |
| Q13 | <--- | Autonomous_learning | 1.043 | .086 | 12.099 | *** | par_5 |
| Q4 | <--- | Integrative | 1.000 |  |  |  |  |
| Q2 | <--- | Integrative | 1.103 | .068 | 16.334 | *** | par_6 |
| Q1 | <--- | Integrative | 1.030 | .066 | 15.599 | *** | par_7 |
| Q7 | <--- | Instrumental | 1.000 |  |  |  |  |
| Q6 | <--- | Instrumental | 1.020 | .066 | 15.351 | *** | par_8 |
| Q5 | <--- | Instrumental | .901 | .063 | 14.281 | *** | par_9 |
| Q9 | <--- | Instrumental | .840 | .063 | 13.426 | *** | par_10 |
| Q16 | <--- | Intrinsic | 1.000 |  |  |  |  |
| Q15 | <--- | Intrinsic | .995 | .042 | 23.755 | *** | par_12 |
| Q14 | <--- | Intrinsic | .874 | .047 | 18.602 | *** | par_13 |

**Standardized Regression Weights: (Group number 1 - Default model)**

|  |  |  | Estimate |
| --- | --- | --- | --- |
| Q8 | <--- | Autonomous_learning | .583 |
| Q10 | <--- | Autonomous_learning | .596 |
| Q3 | <--- | Autonomous_learning | .754 |
| Q11 | <--- | Autonomous_learning | .700 |
| Q12 | <--- | Autonomous_learning | .770 |
| Q13 | <--- | Autonomous_learning | .718 |
| Q4 | <--- | Integrative | .746 |
| Q2 | <--- | Integrative | .802 |
| Q1 | <--- | Integrative | .754 |
| Q7 | <--- | Instrumental | .745 |
| Q6 | <--- | Instrumental | .731 |
| Q5 | <--- | Instrumental | .679 |
| Q9 | <--- | Instrumental | .638 |
| Q16 | <--- | Intrinsic | .888 |
| Q15 | <--- | Intrinsic | .873 |
| Q14 | <--- | Intrinsic | .721 |

**Covariances: (Group number 1 - Default model)**

|  |  |  | Estimate | S.E. | C.R. | P | Label |
| --- | --- | --- | --- | --- | --- | --- | --- |
| Autonomous_learning | <--> | Integrative | .226 | .029 | 7.693 | *** | par_14 |
| Autonomous_learning | <--> | Instrumental | .292 | .034 | 8.494 | *** | par_15 |
| Autonomous_learning | <--> | Intrinsic | .286 | .034 | 8.463 | *** | par_16 |
| Integrative | <--> | Instrumental | .388 | .038 | 10.079 | *** | par_17 |
| Integrative | <--> | Intrinsic | .293 | .034 | 8.542 | *** | par_18 |
| Instrumental | <--> | Intrinsic | .381 | .039 | 9.797 | *** | par_19 |
| e8 | <--> | e10 | .251 | .034 | 7.429 | *** | par_11 |

**Correlations: (Group number 1 - Default model)**

|  |  |  | Estimate |
| --- | --- | --- | --- |
| Autonomous_learning | <--> | Integrative | .554 |
| Autonomous_learning | <--> | Instrumental | .675 |
| Autonomous_learning | <--> | Intrinsic | .592 |
| Integrative | <--> | Instrumental | .780 |
| Integrative | <--> | Intrinsic | .526 |
| Instrumental | <--> | Intrinsic | .645 |
| e8 | <--> | e10 | .395 |

**Variances: (Group number 1 - Default model)**

|  |  |  | Estimate | S.E. | C.R. | P | Label |
| --- | --- | --- | --- | --- | --- | --- | --- |
| Autonomous_learning |  |  | .354 | .053 | 6.738 | *** | par_20 |
| Integrative |  |  | .469 | .051 | 9.122 | *** | par_21 |
| Instrumental |  |  | .528 | .058 | 9.174 | *** | par_22 |
| Intrinsic |  |  | .660 | .054 | 12.116 | *** | par_23 |
| e8 |  |  | .688 | .047 | 14.615 | *** | par_24 |
| e10 |  |  | .584 | .040 | 14.531 | *** | par_25 |
| e3 |  |  | .355 | .028 | 12.607 | *** | par_26 |
| e11 |  |  | .416 | .031 | 13.516 | *** | par_27 |
| e12 |  |  | .390 | .032 | 12.245 | *** | par_28 |
| e13 |  |  | .362 | .027 | 13.251 | *** | par_29 |
| e4 |  |  | .373 | .031 | 12.106 | *** | par_30 |
| e2 |  |  | .316 | .030 | 10.398 | *** | par_31 |
| e1 |  |  | .377 | .032 | 11.895 | *** | par_32 |
| e7 |  |  | .425 | .034 | 12.418 | *** | par_33 |
| e6 |  |  | .479 | .038 | 12.704 | *** | par_34 |
| e5 |  |  | .503 | .037 | 13.562 | *** | par_35 |
| e9 |  |  | .544 | .039 | 14.046 | *** | par_36 |
| e16 |  |  | .177 | .022 | 8.208 | *** | par_37 |
| e15 |  |  | .204 | .022 | 9.091 | *** | par_38 |
| e14 |  |  | .466 | .034 | 13.902 | *** | par_39 |

**Modification Indices (Group number 1 - Default model)**

**Covariances: (Group number 1 - Default model)**

|  |  |  | M.I. | Par Change |
| --- | --- | --- | --- | --- |
| e14 | <--> | Intrinsic | 10.785 | -.071 |
| e14 | <--> | Integrative | 8.579 | .054 |
| e14 | <--> | Autonomous_learning | 4.617 | .035 |
| e15 | <--> | Autonomous_learning | 9.020 | -.037 |
| e9 | <--> | Instrumental | 8.476 | -.054 |
| e9 | <--> | Autonomous_learning | 39.825 | .110 |
| e9 | <--> | e16 | 5.003 | .041 |
| e5 | <--> | Integrative | 9.556 | .059 |
| e6 | <--> | Instrumental | 6.666 | .046 |
| e6 | <--> | Autonomous_learning | 5.961 | -.041 |
| e7 | <--> | e6 | 14.310 | .090 |
| e1 | <--> | Intrinsic | 4.983 | -.046 |
| e1 | <--> | Instrumental | 4.076 | .034 |
| e1 | <--> | e5 | 4.331 | .048 |
| e2 | <--> | e14 | 4.672 | .046 |
| e4 | <--> | Autonomous_learning | 4.397 | .032 |
| e4 | <--> | e1 | 4.217 | -.041 |
| e12 | <--> | Instrumental | 4.866 | -.038 |
| e12 | <--> | Integrative | 9.009 | .053 |
| e12 | <--> | e15 | 5.377 | -.039 |
| e12 | <--> | e16 | 5.260 | .038 |
| e12 | <--> | e5 | 4.327 | .049 |
| e12 | <--> | e6 | 10.239 | -.075 |
| e12 | <--> | e7 | 5.972 | -.054 |
| e12 | <--> | e4 | 8.965 | .063 |
| e12 | <--> | e13 | 4.404 | .041 |
| e3 | <--> | Integrative | 4.054 | -.034 |
| e3 | <--> | e5 | 13.340 | -.081 |
| e3 | <--> | e6 | 6.674 | .057 |
| e3 | <--> | e1 | 5.217 | -.046 |
| e10 | <--> | Intrinsic | 13.881 | .081 |
| e10 | <--> | Autonomous_learning | 9.714 | -.050 |
| e10 | <--> | e15 | 6.839 | .046 |
| e10 | <--> | e9 | 7.083 | .065 |
| e10 | <--> | e12 | 9.075 | -.066 |
| e10 | <--> | e11 | 7.413 | -.059 |
| e10 | <--> | e3 | 9.905 | .065 |
| e8 | <--> | Intrinsic | 7.248 | -.063 |
| e8 | <--> | Instrumental | 20.357 | .087 |
| e8 | <--> | e9 | 38.526 | .165 |

**Variances: (Group number 1 - Default model)**

|  |  |  | M.I. | Par Change |
| --- | --- | --- | --- | --- |

**Regression Weights: (Group number 1 - Default model)**

|  |  |  | M.I. | Par Change |
| --- | --- | --- | --- | --- |
| Q14 | <--- | Instrumental | 9.440 | .147 |
| Q14 | <--- | Integrative | 14.942 | .197 |
| Q14 | <--- | Autonomous_learning | 8.848 | .172 |
| Q14 | <--- | Q5 | 7.294 | .090 |
| Q14 | <--- | Q6 | 7.466 | .086 |
| Q14 | <--- | Q7 | 4.466 | .069 |
| Q14 | <--- | Q1 | 10.847 | .113 |
| Q14 | <--- | Q2 | 15.567 | .134 |
| Q14 | <--- | Q4 | 6.826 | .091 |
| Q14 | <--- | Q13 | 4.959 | .083 |
| Q14 | <--- | Q12 | 4.021 | .066 |
| Q14 | <--- | Q11 | 9.431 | .109 |
| Q14 | <--- | Q3 | 6.138 | .088 |
| Q14 | <--- | Q8 | 5.545 | .074 |
| Q15 | <--- | Autonomous_learning | 5.676 | -.105 |
| Q15 | <--- | Q12 | 9.196 | -.076 |
| Q15 | <--- | Q11 | 7.561 | -.074 |
| Q15 | <--- | Q3 | 4.816 | -.059 |
| Q9 | <--- | Autonomous_learning | 19.567 | .275 |
| Q9 | <--- | Q16 | 5.026 | .084 |
| Q9 | <--- | Q13 | 4.773 | .087 |
| Q9 | <--- | Q12 | 14.831 | .135 |
| Q9 | <--- | Q11 | 4.762 | .083 |
| Q9 | <--- | Q3 | 14.964 | .147 |
| Q9 | <--- | Q10 | 46.948 | .248 |
| Q9 | <--- | Q8 | 76.089 | .294 |
| Q5 | <--- | Q1 | 5.238 | .082 |
| Q5 | <--- | Q3 | 7.069 | -.098 |
| Q6 | <--- | Q7 | 5.449 | .080 |
| Q6 | <--- | Q12 | 9.277 | -.105 |
| Q7 | <--- | Q6 | 5.794 | .076 |
| Q7 | <--- | Q12 | 5.138 | -.074 |
| Q2 | <--- | Q8 | 5.142 | -.065 |
| Q4 | <--- | Intrinsic | 4.711 | .085 |
| Q4 | <--- | Autonomous_learning | 5.204 | .124 |
| Q4 | <--- | Q15 | 5.928 | .079 |
| Q4 | <--- | Q12 | 11.194 | .103 |
| Q4 | <--- | Q10 | 6.282 | .079 |
| Q12 | <--- | Q6 | 4.394 | -.064 |
| Q12 | <--- | Q4 | 7.543 | .092 |
| Q12 | <--- | Q10 | 5.990 | -.079 |
| Q11 | <--- | Q10 | 5.930 | -.078 |
| Q3 | <--- | Q5 | 10.116 | -.096 |
| Q3 | <--- | Q1 | 6.755 | -.081 |
| Q3 | <--- | Q10 | 4.325 | .064 |
| Q10 | <--- | Intrinsic | 11.827 | .141 |
| Q10 | <--- | Instrumental | 4.383 | .099 |
| Q10 | <--- | Q15 | 15.116 | .133 |
| Q10 | <--- | Q16 | 10.707 | .113 |
| Q10 | <--- | Q9 | 10.098 | .105 |
| Q10 | <--- | Q7 | 5.241 | .074 |
| Q10 | <--- | Q4 | 4.871 | .076 |
| Q8 | <--- | Q9 | 30.657 | .198 |

**Model Fit Summary**

**CMIN**

| Model | NPAR | CMIN | DF | P | CMIN/DF |
| --- | --- | --- | --- | --- | --- |
| Default model | 39 | 377.042 | 97 | .000 | 3.887 |
| Saturated model | 136 | .000 | 0 |  |  |
| Independence model | 16 | 4091.288 | 120 | .000 | 34.094 |

**RMR, GFI**

| Model | RMR | GFI | AGFI | PGFI |
| --- | --- | --- | --- | --- |
| Default model | .057 | .920 | .888 | .656 |
| Saturated model | .000 | 1.000 |  |  |
| Independence model | .342 | .289 | .194 | .255 |

**Baseline Comparisons**

| Model | NFI Delta1 | RFI rho1 | IFI Delta2 | TLI rho2 | CFI |
| --- | --- | --- | --- | --- | --- |
| Default model | .908 | .886 | .930 | .913 | .929 |
| Saturated model | 1.000 |  | 1.000 |  | 1.000 |
| Independence model | .000 | .000 | .000 | .000 | .000 |

**Parsimony-Adjusted Measures**

| Model | PRATIO | PNFI | PCFI |
| --- | --- | --- | --- |
| Default model | .808 | .734 | .751 |
| Saturated model | .000 | .000 | .000 |
| Independence model | 1.000 | .000 | .000 |

**NCP**

| Model | NCP | LO 90 | HI 90 |
| --- | --- | --- | --- |
| Default model | 280.042 | 224.155 | 343.500 |
| Saturated model | .000 | .000 | .000 |
| Independence model | 3971.288 | 3765.968 | 4183.876 |

**FMIN**

| Model | FMIN | F0 | LO 90 | HI 90 |
| --- | --- | --- | --- | --- |
| Default model | .739 | .549 | .440 | .674 |
| Saturated model | .000 | .000 | .000 | .000 |
| Independence model | 8.022 | 7.787 | 7.384 | 8.204 |

**RMSEA**

| Model | RMSEA | LO 90 | HI 90 | PCLOSE |
| --- | --- | --- | --- | --- |
| Default model | .075 | .067 | .083 | .000 |
| Independence model | .255 | .248 | .261 | .000 |

**AIC**

| Model | AIC | BCC | BIC | CAIC |
| --- | --- | --- | --- | --- |
| Default model | 455.042 | 457.732 | 620.261 | 659.261 |
| Saturated model | 272.000 | 281.379 | 848.146 | 984.146 |
| Independence model | 4123.288 | 4124.391 | 4191.069 | 4207.069 |

**ECVI**

| Model | ECVI | LO 90 | HI 90 | MECVI |
| --- | --- | --- | --- | --- |
| Default model | .892 | .783 | 1.017 | .898 |
| Saturated model | .533 | .533 | .533 | .552 |
| Independence model | 8.085 | 7.682 | 8.502 | 8.087 |

**HOELTER**

| Model | HOELTER .05 | HOELTER .01 |
| --- | --- | --- |
| Default model | 164 | 179 |
| Independence model | 19 | 20 |
