# Supplementary material for: The impact of the flipped classroom on the motivation and academic performance of Chinese college English learners
Source: PLoS One. 2025 May 2;20(5):e0322094. doi: 10.1371/journal.pone.0322094 (PMC12047774; doi:10.1371/journal.pone.0322094)
Supplement: S1 File — (ZIP) [file pone.0322094.s001.zip › S1/Confirmatory Factor Analysis of the Model—Autonomous Learning Motivation.docx]

**Confirmatory Factor Analysis of the Model—Autonomous Learning Motivation**

**
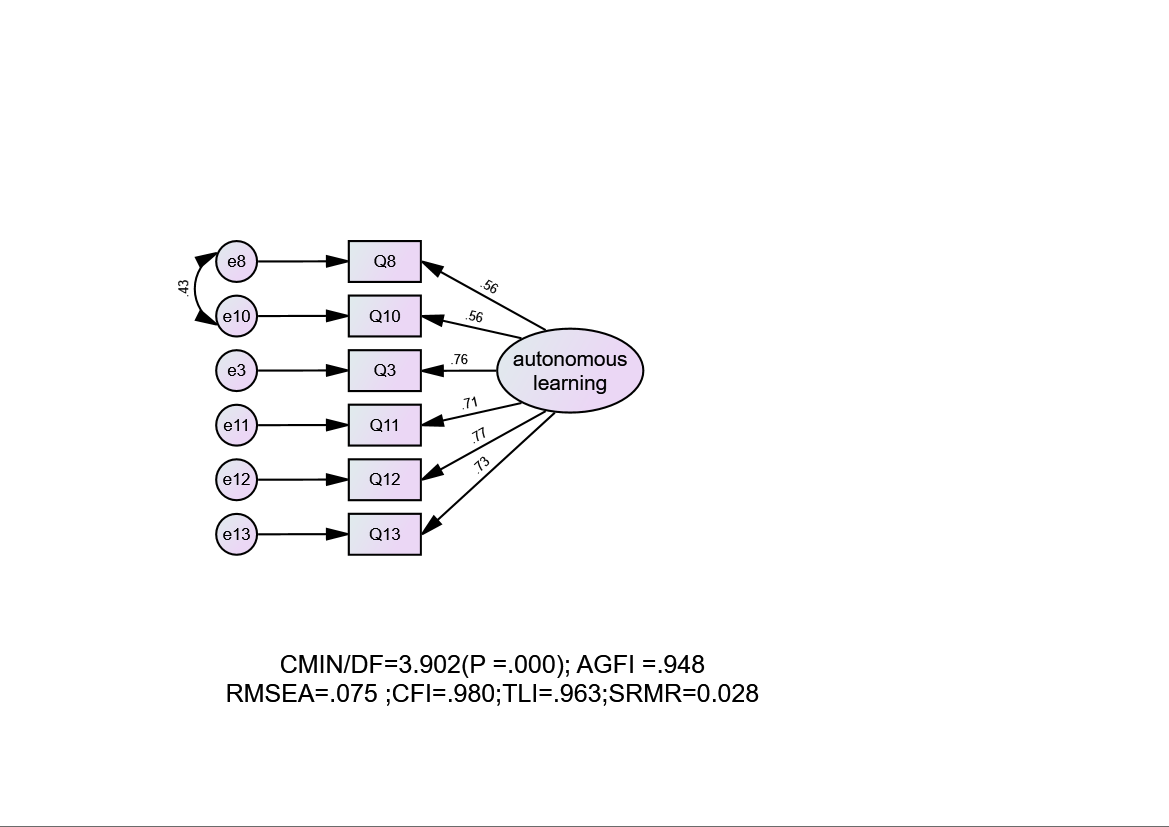
**

| **Estimates (Group number 1 - Default model)** | | | | | | | | | |  | | |  | | |  | | |  | |  | |  |  |
| --- | --- | --- | --- | --- | --- | --- | --- | --- | --- | --- | --- | --- | --- | --- | --- | --- | --- | --- | --- | --- | --- | --- | --- | --- |
|  | |  | | |  | |  | | |  | | |  | | |  | | |  | |  | |  |  |
| **Scalar Estimates (Group number 1 - Default model)** | | | | | | | | | | | | |  | | |  | | |  | |  | |  |  |
|  | |  | | |  | |  | | |  | | |  | | |  | | |  | |  | |  |  |
| **Maximum Likelihood Estimates** | | | | | | |  | | |  | | |  | | |  | | |  | |  | |  |  |
|  | |  | | |  | |  | | |  | | |  | | |  | | |  | |  | |  |  |
|  | | |  | | |  | |  | | |  | | |  | | |  |  |  |  |  |  |  |  |
| **Regression Weights: (Group number 1 - Default model)** | | | | | | | | | | | | | | |  | | |  | |  | |  |  |  |
|  |  | | |  | | | | |  | | |  | | |  | | |  | |  | |  |  |  |
|  |  | | |  | | | | | **Estimate** | | | **S.E.** | | | **C.R.** | | | **P** | | **Label** | |  |  |  |
| Q13 | <--- | | | autonomous_learning | | | | | 1 | | |  | | |  | | |  | |  | |  |  |  |
| Q12 | <--- | | | autonomous_learning | | | | | 1.196 | | | 0.076 | | | 15.668 | | | *** | |  | |  |  |  |
| Q11 | <--- | | | autonomous_learning | | | | | 1.013 | | | 0.07 | | | 14.53 | | | *** | |  | |  |  |  |
| Q3 | <--- | | | autonomous_learning | | | | | 1.096 | | | 0.071 | | | 15.536 | | | *** | |  | |  |  |  |
| Q10 | <--- | | | autonomous_learning | | | | | 0.844 | | | 0.073 | | | 11.547 | | | *** | |  | |  |  |  |
| Q8 | <--- | | | autonomous_learning | | | | | 0.905 | | | 0.078 | | | 11.54 | | | *** | |  | |  |  |  |

**Standardized Regression Weights: (Group number 1 - Default model)**

|  |  |  | **Estimate** |
| --- | --- | --- | --- |
| Q13 | <--- | autonomous_learning | .729 |
| Q12 | <--- | autonomous_learning | .771 |
| Q11 | <--- | autonomous_learning | .707 |
| Q3 | <--- | autonomous_learning | .763 |
| Q10 | <--- | autonomous_learning | .559 |
| Q8 | <--- | autonomous_learning | .559 |

**Covariances: (Group number 1 - Default model)**

|  | | |  |  | **Estimate** | | **S.E.** | | | **C.R.** | | **P** | | | **Label** |  |  |  |
| --- | --- | --- | --- | --- | --- | --- | --- | --- | --- | --- | --- | --- | --- | --- | --- | --- | --- | --- |
| e10 | | | <--> | e8 | .284 | | .036 | | | 7.946 | | *** | | |  |  |  |  |
| **Correlations: (Group number 1 - Default model)** | | | | | | | | | | | | | | |  |  |  |  |
|  | | | |  | |  | | | | | |  | | |  |  |  |  |
|  | | | |  | |  | | | | | | **Estimate** | | |  |  |  |  |
| e10 | | | | <--> | | e8 | | | | | | 0.426 | | |  |  |  |  |
| **Variances: (Group number 1 - Default model)** | | | | | | | | | | | |  | | |  |  |  |  |
|  | | | | | | | |  | |  | |  | | |  |  |  |  |
|  | | | | | | | |  | |  | | **Estimate** | | | **S.E.** | **C.R.** | **P** | **Label** |
| **autonomous_learning** | | | | | | | |  | |  | | 0.398 | | | 0.045 | 8.899 | *** |  |
| **e13** | | | | | | | |  | |  | | 0.35 | | | 0.028 | 12.64 | *** |  |
| **e12** | | | | | | | |  | |  | | 0.389 | | | 0.033 | 11.67 | *** |  |
| **e11** | | | | | | | |  | |  | | 0.408 | | | 0.031 | 13.05 | *** |  |
| **e3** | | | | | | | |  | |  | | 0.344 | | | 0.029 | 11.88 | *** |  |
| **e10** | | | | | | | |  | |  | | 0.622 | | | 0.043 | 14.62 | *** |  |
| **e8** | | | | | | | |  | |  | | 0.717 | | | 0.049 | 14.62 | *** |  |

**Modification Indices (Group number 1 - Default model)**

**Covariances: (Group number 1 - Default model)**

|  |  |  | **M.I.** | **Par Change** |  |
| --- | --- | --- | --- | --- | --- |
| e3 | <--> | e10 | 16.911 | 0.087 |  |
| e12 | <--> | e3 | 5.093 | -0.045 |  |
|  |  |  |  |  |  |
| **Variances: (Group number 1 - Default model)** | | | | |  |
|  |  |  |  |  |  |
|  |  |  | **M.I.** | **Par Change** |  |
|  |  |  |  |  |  |
| **Regression Weights: (Group number 1 - Default model)** | | | | | |
|  |  |  |  |  |  |
|  |  |  | **M.I.** | **Par Change** |  |
| Q10 | <--- | Q3 | 5.634 | 0.084 |  |
| Q3 | <--- | Q10 | 9.2 | 0.093 |  |

**Model Fit Summary**

**CMIN**

| **Model** | **NPAR** | **CMIN** | **DF** | **P** | **CMIN/DF** |
| --- | --- | --- | --- | --- | --- |
| **Default model** | 13 | 31.214 | 8 | .000 | 3.902 |
| **Saturated model** | 21 | .000 | 0 |  |  |
| **Independence model** | 6 | 1195.677 | 15 | .000 | 79.712 |

**RMR, GFI**

| **Model** | **RMR** | **GFI** | **AGFI** | **PGFI** |
| --- | --- | --- | --- | --- |
| **Default model** | .023 | .980 | .948 | .373 |
| **Saturated model** | .000 | 1.000 |  |  |
| **Independence model** | .362 | .456 | .238 | .326 |

**Baseline Comparisons**

| **Model** | **NFI Delta1** | **RFI rho1** | **IFI Delta2** | **TLI rho2** | **CFI** |
| --- | --- | --- | --- | --- | --- |
| **Default model** | .974 | .951 | .980 | .963 | .980 |
| **Saturated model** | 1.000 |  | 1.000 |  | 1.000 |
| **Independence model** | .000 | .000 | .000 | .000 | .000 |

**Parsimony-Adjusted Measures**

| **Model** | **PRATIO** | **PNFI** | **PCFI** |
| --- | --- | --- | --- |
| **Default model** | .533 | .519 | .523 |
| **Saturated model** | .000 | .000 | .000 |
| **Independence model** | 1.000 | .000 | .000 |

**NCP**

| **Model** | **NCP** | **LO 90** | **HI 90** |
| --- | --- | --- | --- |
| **Default model** | 23.214 | 9.685 | 44.299 |
| **Saturated model** | .000 | .000 | .000 |
| **Independence model** | 1180.677 | 1070.928 | 1297.803 |

**FMIN**

| **Model** | **FMIN** | **F0** | **LO 90** | **HI 90** |
| --- | --- | --- | --- | --- |
| **Default model** | .061 | .046 | .019 | .087 |
| **Saturated model** | .000 | .000 | .000 | .000 |
| **Independence model** | 2.344 | 2.315 | 2.100 | 2.545 |

**RMSEA**

| **Model** | **RMSEA** | **LO 90** | **HI 90** | **PCLOSE** |
| --- | --- | --- | --- | --- |
| **Default model** | .075 | .049 | .104 | .058 |
| **Independence model** | .393 | .374 | .412 | .000 |

**AIC**

| **Model** | **AIC** | **BCC** | **BIC** | **CAIC** |
| --- | --- | --- | --- | --- |
| **Default model** | 57.214 | 57.576 | 112.287 | 125.287 |
| **Saturated model** | 42.000 | 42.584 | 130.964 | 151.964 |
| **Independence model** | 1207.677 | 1207.844 | 1233.096 | 1239.096 |

**ECVI**

| **Model** | **ECVI** | **LO 90** | **HI 90** | **MECVI** |
| --- | --- | --- | --- | --- |
| **Default model** | .112 | .086 | .154 | .113 |
| **Saturated model** | .082 | .082 | .082 | .083 |
| **Independence model** | 2.368 | 2.153 | 2.598 | 2.368 |

**HOELTER**

| **Model** | **HOELTER .05** | **HOELTER .01** |
| --- | --- | --- |
| **Default model** | 254 | 329 |
| **Independence model** | 11 | 14 |
