# Supplementary material for: The impact of the flipped classroom on the motivation and academic performance of Chinese college English learners
Source: PLoS One. 2025 May 2;20(5):e0322094. doi: 10.1371/journal.pone.0322094 (PMC12047774; doi:10.1371/journal.pone.0322094)
Supplement: S1 File — (ZIP) [file pone.0322094.s001.zip › S1/Confirmatory Factor Analysis of the Model—Instrumental Motivation.docx]

**Confirmatory Factor Analysis of the Model—Instrumental Motivation**


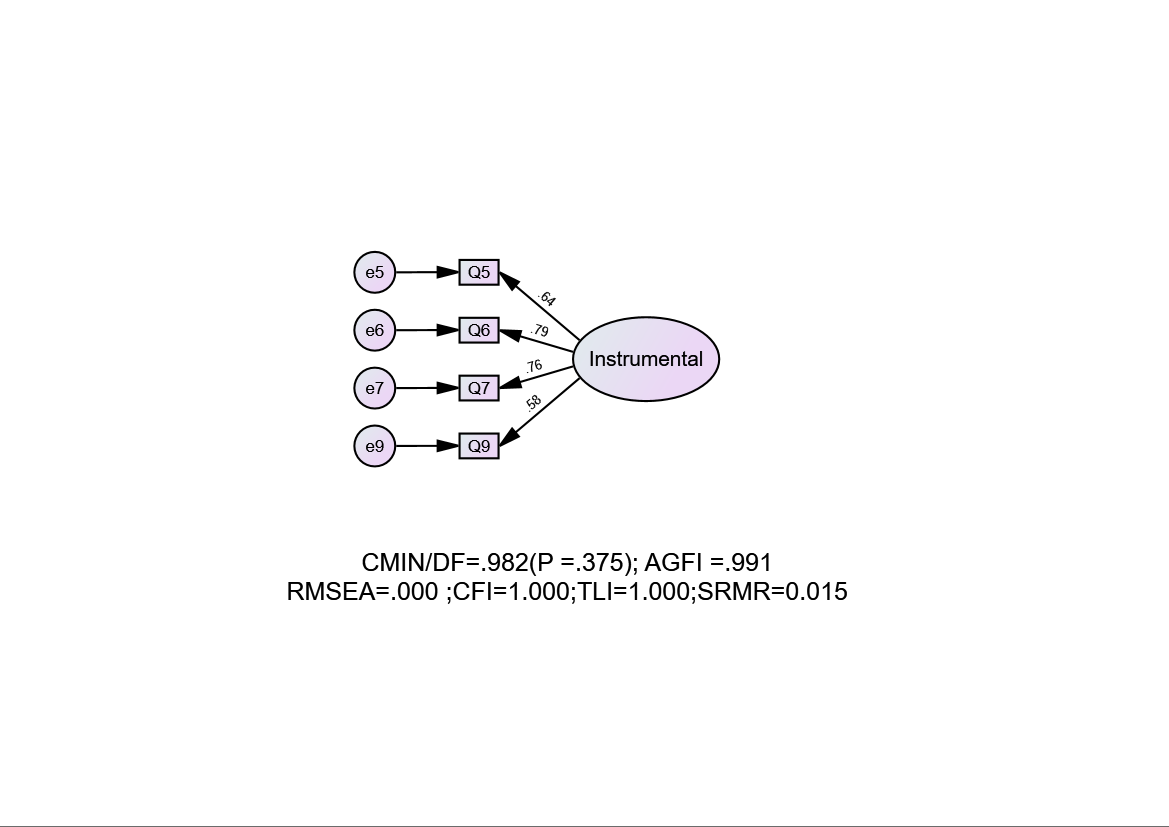


**Estimates (Group number 1 - Default model)**

**Scalar Estimates (Group number 1 - Default model)**

**Maximum Likelihood Estimates**

**Regression Weights: (Group number 1 - Default model)**

|  |  |  | **Estimate** | **S.E.** | **C.R.** | **P** | **Label** |
| --- | --- | --- | --- | --- | --- | --- | --- |
| Q9 | <--- | Instrumental | .896 | .084 | 10.672 | *** |  |
| Q7 | <--- | Instrumental | 1.201 | .093 | 12.851 | *** |  |
| Q6 | <--- | Instrumental | 1.287 | .099 | 12.963 | *** |  |
| Q5 | <--- | Instrumental | 1.000 |  |  |  |  |

**Standardized Regression Weights: (Group number 1 - Default model)**

|  |  |  | **Estimate** |
| --- | --- | --- | --- |
| Q9 | <--- | Instrumental | .581 |
| Q7 | <--- | Instrumental | .764 |
| Q6 | <--- | Instrumental | .788 |
| Q5 | <--- | Instrumental | .643 |

**Variances: (Group number 1 - Default model)**

|  |  |  | **Estimate** | **S.E.** | **C.R.** | **P** | **Label** |
| --- | --- | --- | --- | --- | --- | --- | --- |
| **Instrumental** |  |  | .386 | .053 | 7.314 | *** |  |
| **e9** |  |  | .607 | .043 | 13.991 | *** |  |
| **e7** |  |  | .396 | .039 | 10.157 | *** |  |
| **e6** |  |  | .390 | .042 | 9.279 | *** |  |
| **e5** |  |  | .546 | .041 | 13.191 | *** |  |

**Model Fit Summary**

**CMIN**

| **Model** | **NPAR** | **CMIN** | **DF** | **P** | **CMIN/DF** |
| --- | --- | --- | --- | --- | --- |
| **Default model** | 8 | 1.964 | 2 | .375 | .982 |
| **Saturated model** | 10 | .000 | 0 |  |  |
| **Independence model** | 4 | 580.368 | 6 | .000 | 96.728 |

**RMR, GFI**

| **Model** | **RMR** | **GFI** | **AGFI** | **PGFI** |
| --- | --- | --- | --- | --- |
| **Default model** | .010 | .998 | .991 | .200 |
| **Saturated model** | .000 | 1.000 |  |  |
| **Independence model** | .362 | .586 | .310 | .352 |

**Baseline Comparisons**

| **Model** | **NFI Delta1** | **RFI rho1** | **IFI Delta2** | **TLI rho2** | **CFI** |
| --- | --- | --- | --- | --- | --- |
| **Default model** | .997 | .990 | 1.000 | 1.000 | 1.000 |
| **Saturated model** | 1.000 |  | 1.000 |  | 1.000 |
| **Independence model** | .000 | .000 | .000 | .000 | .000 |

**Parsimony-Adjusted Measures**

| **Model** | **PRATIO** | **PNFI** | **PCFI** |
| --- | --- | --- | --- |
| **Default model** | .333 | .332 | .333 |
| **Saturated model** | .000 | .000 | .000 |
| **Independence model** | 1.000 | .000 | .000 |

**NCP**

| **Model** | **NCP** | **LO 90** | **HI 90** |
| --- | --- | --- | --- |
| **Default model** | .000 | .000 | 7.752 |
| **Saturated model** | .000 | .000 | .000 |
| **Independence model** | 574.368 | 498.980 | 657.156 |

**FMIN**

| **Model** | **FMIN** | **F0** | **LO 90** | **HI 90** |
| --- | --- | --- | --- | --- |
| **Default model** | .004 | .000 | .000 | .015 |
| **Saturated model** | .000 | .000 | .000 | .000 |
| **Independence model** | 1.138 | 1.126 | .978 | 1.289 |

**RMSEA**

| **Model** | **RMSEA** | **LO 90** | **HI 90** | **PCLOSE** |
| --- | --- | --- | --- | --- |
| **Default model** | .000 | .000 | .087 | .714 |
| **Independence model** | .433 | .404 | .463 | .000 |

**AIC**

| **Model** | **AIC** | **BCC** | **BIC** | **CAIC** |
| --- | --- | --- | --- | --- |
| **Default model** | 17.964 | 18.122 | 51.855 | 59.855 |
| **Saturated model** | 20.000 | 20.198 | 62.364 | 72.364 |
| **Independence model** | 588.368 | 588.447 | 605.313 | 609.313 |

**ECVI**

| **Model** | **ECVI** | **LO 90** | **HI 90** | **MECVI** |
| --- | --- | --- | --- | --- |
| **Default model** | .035 | .035 | .050 | .036 |
| **Saturated model** | .039 | .039 | .039 | .040 |
| **Independence model** | 1.154 | 1.006 | 1.316 | 1.154 |

**HOELTER**

| **Model** | **HOELTER .05** | **HOELTER .01** |
| --- | --- | --- |
| **Default model** | 1557 | 2393 |
| **Independence model** | 12 | 15 |
