# Supplementary material for: The impact of the flipped classroom on the motivation and academic performance of Chinese college English learners
Source: PLoS One. 2025 May 2;20(5):e0322094. doi: 10.1371/journal.pone.0322094 (PMC12047774; doi:10.1371/journal.pone.0322094)
Supplement: S1 File — (ZIP) [file pone.0322094.s001.zip › S1/Exploratory Factor Analysis of English Learning Motivation Scale.docx]

**Exploratory Factor Analysis of English Learning Motivation Scale**

| **KMO and Bartlett's Test** | | | | | |
| --- | --- | --- | --- | --- | --- |
| Kaiser-Meyer-Olkin Measure of Sampling Adequacy. | | | | | .901 |
| Bartlett's Test of Sphericity | | | Approx. Chi-Square | | 1158.560 |
|  |  |  | df | | 120 |
|  |  |  | Sig. | | .000 |
| **Communalities** | | | |  |  |
|  | Initial | Extraction | |  |  |
| Q3 | 1.000 | .654 | |  |  |
| Q8 | 1.000 | .786 | |  |  |
| Q10 | 1.000 | .761 | |  |  |
| Q11 | 1.000 | .688 | |  |  |
| Q12 | 1.000 | .718 | |  |  |
| Q13 | 1.000 | .668 | |  |  |
| Q1 | 1.000 | .865 | |  |  |
| Q2 | 1.000 | .823 | |  |  |
| Q4 | 1.000 | .864 | |  |  |
| Q5 | 1.000 | .793 | |  |  |
| Q6 | 1.000 | .833 | |  |  |
| Q7 | 1.000 | .775 | |  |  |
| Q9 | 1.000 | .810 | |  |  |
| Q14 | 1.000 | .817 | |  |  |
| Q15 | 1.000 | .709 | |  |  |
| Q16 | 1.000 | .816 | |  |  |
| Extraction Method: Principal Component Analysis. | | | |  |  |

**
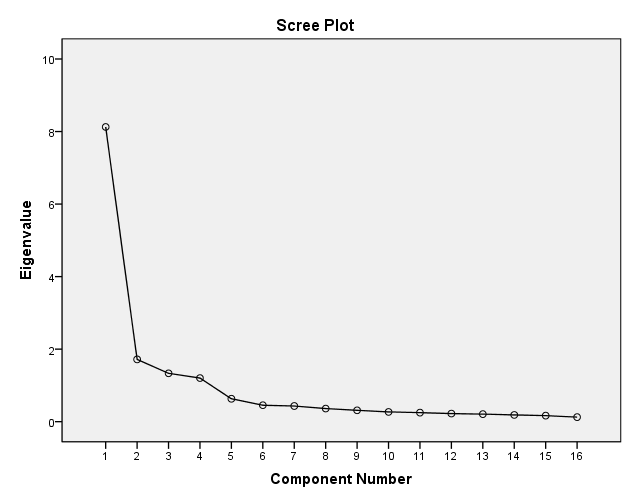
**

| **Total Variance Explained** | | | | | | | | | |
| --- | --- | --- | --- | --- | --- | --- | --- | --- | --- |
| Component | Initial Eigenvalues | | | Extraction Sums of Squared Loadings | | | Rotation Sums of Squared Loadings | | |
|  | Total | % of Variance | Cumulative % | Total | % of Variance | Cumulative % | Total | % of Variance | Cumulative % |
| 1 | 8.126 | 50.788 | 50.788 | 8.126 | 50.788 | 50.788 | 3.975 | 24.847 | 24.847 |
| 2 | 1.717 | 10.733 | 61.521 | 1.717 | 10.733 | 61.521 | 3.194 | 19.963 | 44.810 |
| 3 | 1.334 | 8.335 | 69.857 | 1.334 | 8.335 | 69.857 | 2.641 | 16.505 | 61.315 |
| 4 | 1.203 | 7.519 | 77.375 | 1.203 | 7.519 | 77.375 | 2.570 | 16.060 | 77.375 |
| 5 | .632 | 3.949 | 81.324 |  |  |  |  |  |  |
| 6 | .454 | 2.836 | 84.160 |  |  |  |  |  |  |
| 7 | .432 | 2.699 | 86.859 |  |  |  |  |  |  |
| 8 | .361 | 2.259 | 89.117 |  |  |  |  |  |  |
| 9 | .314 | 1.960 | 91.077 |  |  |  |  |  |  |
| 10 | .269 | 1.682 | 92.759 |  |  |  |  |  |  |
| 11 | .249 | 1.556 | 94.315 |  |  |  |  |  |  |
| 12 | .223 | 1.393 | 95.708 |  |  |  |  |  |  |
| 13 | .209 | 1.304 | 97.012 |  |  |  |  |  |  |
| 14 | .186 | 1.165 | 98.177 |  |  |  |  |  |  |
| 15 | .166 | 1.040 | 99.217 |  |  |  |  |  |  |
| 16 | .125 | .783 | 100.000 |  |  |  |  |  |  |
| Extraction Method: Principal Component Analysis. | | | | | | | | | |

| **Rotated Component Matrix^a^** | | | | |
| --- | --- | --- | --- | --- |
|  | Component | | | |
|  | 1 | 2 | 3 | 4 |
| Q12 | .794 |  |  |  |
| Q8 | .789 |  |  |  |
| Q11 | .745 |  |  |  |
| Q3 | .741 |  |  |  |
| Q10 | .724 |  |  |  |
| Q13 | .699 |  |  |  |
| Q6 |  | .868 |  |  |
| Q9 |  | .823 |  |  |
| Q5 |  | .793 |  |  |
| Q7 |  | .767 |  |  |
| Q1 |  |  | .877 |  |
| Q4 |  |  | .870 |  |
| Q2 |  |  | .802 |  |
| Q16 |  |  |  | .841 |
| Q14 |  |  |  | .818 |
| Q15 |  |  |  | .738 |
| Extraction Method: Principal Component Analysis.   Rotation Method: Varimax with Kaiser Normalization. | | | | |
| a. Rotation converged in 5 iterations. | | | | |

| **Component Transformation Matrix** | | | | |
| --- | --- | --- | --- | --- |
| Component | 1 | 2 | 3 | 4 |
| 1 | .615 | .505 | .422 | .434 |
| 2 | -.434 | .663 | -.508 | .338 |
| 3 | -.657 | .073 | .740 | .126 |
| 4 | -.046 | -.548 | -.127 | .826 |
| Extraction Method: Principal Component Analysis.   Rotation Method: Varimax with Kaiser Normalization. | | | | |
