# Supplementary material for: The impact of the flipped classroom on the motivation and academic performance of Chinese college English learners
Source: PLoS One. 2025 May 2;20(5):e0322094. doi: 10.1371/journal.pone.0322094 (PMC12047774; doi:10.1371/journal.pone.0322094)
Supplement: S1 File — (ZIP) [file pone.0322094.s001.zip › S1/post-test for English proficiency.rtf]

	

Part ¢ñ   Listening Comprehension 

Section A
Directions: In this section, you will hear several conversations. At the end of each conversation, one or more questions will be asked about what was said. Both the conversations and the questions will be spoken only once. After each question there will be a pause. During the pause, you must read the four choices marked A), B), C) and D), and decide which is the best answer.

1.

	A) She has to post a letter instead.

B) She has to turn down the man's request.

C) She's not sure if the computer is fixed.

D) She can't send the message right now.

	
2.

	A) The entertaining stories.

B) The good atmosphere the cinema offers.

C) The reasons for going to the cinema.

D) The feedback to different movies.

	
3.

	A) Martha will have class at 2:00.

B) Martha will go to the library at 3:00.

C) Martha will go home immediately after class.

D) Martha will go home after 4:00.

	
4.

	A) It will not be easy.

B) It will be less difficult.

C) It has been boring.

D) It has been enjoyable.

	
5.

	A) He dressed too formally.

B) He always buys very expensive suits and ties.

C) The guy is dressed to attract lot of attention and admiration.

D) She doesn't like the style the man gets dressed.

	
6.

	A) Decide whether they should go to the movies.

B) Decide which food to choose.

C) Go to a movie a little later.

D) Choose a restaurant as soon as possible.

	
7.

	A) Who made the phone call.

B) What the call was about.

C) Who came earlier than he.

D) When John called him.

	
8.

	A) A menu.

B) A shopping list.

C) A refrigerator.

D) A cooking book.

	

Questions 9 to 12 are based on the conversation you have just heard.

9.

	A) At a newspaper office.

B) At an advertising agency.

C) At a furniture store.

D) At a real estate agency.

	
10.

	A) A twoª²bedroom apartment.

B) A sofa.

C) A chair.

D) A roommate.

	
11.

	A) Her phone number.

B) The location of the apartment.

C) The best time to call her.

D) Her first name.

	
12.

	A) $5.

B) $15.

C) $30.

D) $250.

	

Questions 13 to 15 are based on the conversation you have just heard.

13.

	A) Close friends.

B) Classmates.

C) Husband and wife.

D) Employer and employee.

	
14.

	A) Typing too many mistakes in letters.

B) Smoking in the office.

C) Throwing litters on the floor.

D) Being so forgetful.

	
15.

	A) The woman get it lost.

B) It is still in their office.

C) Borrowed by other colleagues.

D) In the next door company's office.

	

Section B
Directions: In this section, you will hear several short passages. At the end of each passage, you will hear some questions. Both the passage and the questions will be spoken only once. After you hear a question, you must choose the best answer from the four choices marked A), B), C) and D).

Passage One
Questions 16 to 18 are based on the passage you have just heard.

16.

	A) Children attend school for more years.

B) Children are better dressed.

C) Children spend more money on entertainment.

D) All of the above.

	
17.

	A) Children grow up feeling unsettled and parents didn't pay much attention to children.

B) Children grow up feeling free and one parent is responsible for raising the children.

C) Children are moved back and forth between parents and the single parent is busy working to make money to support himself/herself.

D) Children grow up feeling unsettled, and the parents have little time for his/her own interests because one parent is too busy taking care of children.

	
18.

	A) Become worse.

B) Remain the same.

C) Get better.

D) Keep unchanged.

	

Passage Two
Questions 19 to 22 are based on the passage you have just heard.

19.

	A) In 1896.

B) In 1900.

C) In 1904.

D) In 1915.

	
20.

	A) Pottery-design.

B) Sculpture.

C) Fashion show.

D) Book illustration.

	
21.

	A) The Royal Academy of Art.

B) European and American galleries.

C) The School of Paris.

D) The South of France.

	
22.

	A) All over the world.

B) Every European and American gallery.

C) Most French galleries.

D) Most important European and American galleries.

	

Passage Three
Questions 23 to 26 are based on the passage you have just heard.

23.

	A) For some poor persons who are hungry.

B) For the poor who can't stand poverty.

C) For some rich people who have known poverty.

D) For a rich person who knows food is important to health.

	
24.

	A) He looks at his face.

B) He looks straight at their feet.

C) He looks down upon them.

D) He looks up and down.

	
25.

	A) Ian Wright has not spent most of his life controlled and protected from the outside world.

B) Ian Wright has been a host of a chat-show for several years.

C) Ian Wright is promoted under the slogan.

D) Ian Wright was born in 1965.

	
26.

	A) His parents.

B) His classmates.

C) His girlfriend.

D) His brothers.

	


Part ¢ò   Reading Comprehension

Section A
Directions: There are several passages in this section. Each passage is followed by some questions or unfinished statements. For each of them there are four choices marked A), B), C) and D). You should decide on the best choice.

Passage One
Questions 27 to 31 are based on the following passage.

       In America, there are more households with pets than those with children. At least 43 percent of U.S. homes have pets of some sort. Common pets include tropical fish, mice and birds. But the all-time favorites are cats and dogs, even at the White House. Americans sometimes have strong feelings about whether dogs or cats make better pets. "Dog people" and "cat people" often enjoy friendly rivalries.
        Leading a dog's life in America isn't such a bad thing. Many grocery stores sell gourmet pet foods to owners eager to please their pets. In Houston, Texas, dogs can have their dinner delivered to their homes, just like pizza. Pets can even accompany their owners on vacation. Fancy hotels are beginning to accommodate both man and beast. Furry guests at Four Seasons Hotels can enjoy gourmet meals served on fine china and sleep in soft beds.
        The average American enjoys having pets around, and for good reason. Researchers have discovered that interacting with animals lowers a person's blood pressure. Dogs can offer protection from burglars and unwelcome visitors. Cats can help rid the home of unwanted pests. Little creatures of all shapes and sizes can provide companionship and love. In many cases, having a pet prepares a young couple for the responsibilities of parenthood. Pets even encourage social relationships: They give their owners an appearance of friendliness, and they provide a good topic of conversation.


27.   	The "make" in the first paragraph means the same with that in ______	
      	A) "You will make a good engineer."

B) "I just can't make it."

C) "Jenny made a promise to him."

D) "My father knows how to make a stool."

	
28.   	"Dog people" and "cat people" ______.	
      	A) hate each other

B) are not friendly

C) like different pets

D) lead different lives

	
29.   	Dogs ______.	
      	A) are liked by more people

B) can sleep in beds in some hotels

C) are more useful than cats

D) have to stay at home when their owners are on vacation

	
30.   	In terms of health, ______.	
      	A) leading a dog's life is beneficial

B) raising pets can do good to us

C) leading a cat's life is beneficial

D) cats are more helpful

	
31.   	Having a pet ______.	
      	A) will occupy a lot of your leisure time

B) can help you kill time

C) can help you get the sense of being a parent

D) brings you a lot of trouble

	

Passage Two
Questions 32 to 36 are based on the following passage.

       Every thought and emotion you have constantly contributes to the state of your overall health. Scientists have now proven that being kind boosts your immune system. Can you believe it? Isn't that amazing? But of course we already knew that, didn't we? The body mirrors the mind perfectly; so what takes place in the mind, takes place in the body, thus the mind-body connection. 
        When we experience an emotion, a chemical reaction takes place within our body. An emotion is felt and a corresponding hormone is released. For example when we feel stressed our body releases adrenaline into our system to help cope with it. Alternatively when we feel joyous our body releases endorphins, which provide a sense of satisfaction and well being. It is this chemical release that connects the mind to the body and impacts our health. 
        The chemicals released into our system attach to our cells by way of receptors. All cells have receptors and are therefore capable of connecting with the hormones from every emotion we feel. When the chemical attaches to the cell, it sends a signal into the cell that in turn creates a biochemical event inside the cell. Enough of the heavy stuff though, basically what that means is it changes the state of the cell and therefore changes you internally. 
        We as human beings have an enormous array of emotions we feel continuously, whether we are aware of it on a conscious level or not. All emotions have a charge that we feel as positive or negative. Moment by moment we are releasing emotional charges into our bodies and most often unaware that we are feeling anything at all. In society as a whole, we have become so totally disconnected from our emotions. 
        When we experience any of the positive emotions such as happiness, joy, passion or love, our bodies are in a state of homeostasis which is physiological equilibrium within our bodies involving a balance of functions and chemical composition. Basically our whole body is in balance and we feel amazing. 
        Positive emotions allow us to feel good physically, mentally and emotionally. The more we feel good, the more we feel good. So here is a perfect reason why we would endeavor to live our life from a place of happiness.


32.   	Which of the following cannot exert influences on human beings' physical health?	
      	A)  Individual's various emotions.

B)  Individual's different thoughts.

C)  Individual's mental health.

D)  Individual's chemical reactions.

	
33.   	Which of the following statements is correct according to the passage?	
      	A)  Only positive emotions can lead to the release of a corresponding hormone.

B)  Endorphins can be released into our system if we face tight working schedule.

C)  Adrenaline can be released into our system if we successfully finished our tasks.

D)  The chemicals released may create a biochemical event in the cell which results in internal changes.

	
34.   	When we experience some positive emotions, we are _______________.	
      	A)  involved in a balance of functions and chemical reactions

B)  experiencing a state of balance and amazement

C)  experiencing a state of physical equilibrium

D)  establishing a new state of balance

	
35.   	Why do people become disconnected from their emotions?	
      	A)  Because they can't feel emotions continuously.

B)  Because they only have negative emotions.

C)  Because the emotions they feel don't release charges in their bodies.

D)  Because they are not conscious of the emotions they are feeling.

	
36.   	Which of the following is the best title of this passage?	
      	A)  Chemical Reactions Happening in Our Bodies

B)  Positive Emotions and Negative Emotions

C)  Emotions and Health

D)  How to Live a Happy Life

	

Passage Three
Questions 37 to 41 are based on the following passage.

       To live through a really big hurricane is to experience terror that leaves you speechless and shaken. It is an unreal nightmare of violence and fear. Hurricane Camille, which visited our southern coast on August 17, 1969, was just such a hurricane.
        As a result of the relentless pounding and fury of the winds, 19,467 homes and 700 businesses were destroyed and 241 people were killed. Hurricane warnings had been broadcast, and 150,000 people heeded them. But others thought they could ride out the storm. They didn't know what they were in for. In the darkness, rain and terrifying winds pounded homes and battered down walls. The electricity went off, and houses tumbled from their foundations and were smashed to pieces. Cargo ships snapped from their moorings.
        Seawater 25 to 30 feet deep poured in upon the unfortunate residents. In Gulfport, Mississippi, a 900,000 gallon oil tank was hurled 3.5 miles from its original site. Telephone poles snapped like toothpicks. The roar was deafening as winds quickly gusted to 200 miles per hour. Everything in a 70-mile-wide path was devastated.
        On August 18, residents returned to an unbelievable pile of wreckage dotted with human and animal bodies. The federal government sent in over 200 tons of food and hundreds of mobile homes and classrooms. The cleanup took many months and a prodigious amount of hard work. Even though the storm was over, no one who lived through it would ever forget the force of Hurricane Camille.


37.   	Which of the following evidence was used to support the statement "A hurricane is an unreal nightmare of violence and fear"?	
      	A) During the storm, houses were smashed and a lot of people died.

B) Oil tankers loaded and unloaded at Gulfport, Mississippi.

C) Telephone poles broke in two because of the strong winds.

D) Electrical power went off for a short time during a storm.

	
38.   	It is likely that the 150,000 people who heeded the warnings about the hurricane __________.	
      	A) stayed in their homes

B) left their homes for safer locations

C) waited until the National Guard came to help them

D) rode out the storm

	
39.   	Which of the following information is NOT included in the story?	
      	A) During the storm, cargo ships were forced away from their moorings.

B) Television news programs spent several days covering the aftermath of Hurricane Camille.

C) The storm winds gusted up to 200 miles per hour.

D) The federal government extended its hand to help.

	
40.   	What does the word "prodigious" in paragraph 4 mean?	
      	A) Huge.

B) Short.

C) Modest.

D) Slight.

	
41.   	The author wants us to think that __________.	
      	A) people are foolish to be frightened of hurricanes

B) a hurricane is one of the most violent storms in nature

C) people who are fortunate enough to experience a hurricane are lucky

D) a hurricane can smash houses to pieces

	

Section B
Directions: In this section, there is a passage with several blanks. You are required to select one word for each blank from a list of choices given in a word bank following the passage. Read the passage through carefully before making your choices. Each choice in the bank is identified by a letter. You may not use any of the words in the bank more than once.

Questions 42-51 are based on the following passage.

       There are two things I have always wanted to do — write and live on a farm. Today I'm doing both. I am not in E. B. White's class as a writer or in my neighbors' league as a farmer, but I'm getting by. And after years of   42   with city and suburban living, my wife Sandy and I have finally found   43   here in the country.
        It's a   44   sort of life. We grow nearly all of our fruits and vegetables. Our hens keep us in eggs, with several dozen left over to sell each week. Our bees provide us with honey, and we cut enough wood to just about   45   it _____ the heating season.
        It's a   46   life too. In the summer we canoe on the river, go picnicking in the woods and take long bicycle rides. In the winter we ski and   47   . We get excited about sunsets. We love the   48   of the earth warming and the sound of cattle lowing. We watch for hawks in the sky and deer in the cornfields.
        But the good life can get pretty tough. Three months ago when it was 30 below, we spent two   49   days hauling firewood up the river on a sled. Three months from now, it will be 95 above and we will be   50   corn, weeding strawberries and killing chickens. Recently, Sandy and I had to retile the back roof. Soon Jim, 16 and Emily, 13, the youngest of our four children, will help me make some long-overdue improvements on the outdoor toilet that   51   our indoor plumbing when we are working outside. Later this month, we'll spray the orchard, paint the barn, plant the garden and clean the hen house before the new chicks arrive.

A) self-reliant
B) frustration
C) contentment
D) satisfying

E) supplements
F) skate
G) smell
H) miserable

I) cultivating
J) satisfactory
K) make … through
L) sense

M) mysterious
N) make … up
O) self-respect


	


Part ¢ó   Vocabulary and Grammar 

Directions: There are a number of incomplete sentences in this part. For each sentence there are four choices marked A), B), C) and D). Choose the ONE that best completes the sentence.

52.   	On the morning of Jan. 15, 1976, millions of people, foreign or Chinese, in Beijing took part in the funeral _______ of Premier Zhou Enlai.	
      	A) processions

B) parades

C) lines

D) ranks

	
53.   	"GMT" ______ Greenwich Mean Time.	
      	A) goes for

B) passes for

C) stands for

D) heads for

	
54.   	He commented the amazing way in which computers have recently ________ in size.	
      	A) reduced

B) condensed

C) compressed

D) shrunk

	
55.   	It was required that the manufacturer ________ out the design immediately.	
      	A) worked

B) would work

C) work

D) works

	
56.   	_______ he had said it he knew what a mistake he had made.	
      	A) In the minute

B) To the minute

C) A minute

D) The minute

	
57.   	He is _____ getting promotion, and no one is stopping him!	
      	A) adequate to

B) intent on

C) short of

D) difficult in

	
58.   	All the lights suddenly went off and the house was ______ darkness.	
      	A) plunged into

B) enveloped

C) attacked by

D) merged into

	
59.   	When the first settlers came to America, their ______ utensils consisted of a few pots, pans, and some bowls ______ in one corner of the house.	
      	A) house ... stacked

B) household ... stacked

C) kitchen ... stacking

D) home ... piled

	
60.   	NASA's Mars program was set back when two spacecrafts failed in reaching Mars, one ______ up in the planet's atmosphere and the other ______ after a software failure.	
      	A) burned ... disappeared

B) burned ... disappearing

C) burning ... disappearing

D) burning ... disappeared

	
61.   	The policeman looked at me ______ several times and obviously disliked what he saw.	
      	A) over and over again

B) up and down

C) from side to side

D) in and out

	
62.   	The climb was difficult, but he got to the top of the mountain ________.	
      	A) at length

B) in length

C) in last

D) at the last

	
63.   	 The map was drawn to the standard _________ of 1 to a million, so there was not much detail.	
      	A) ratio

B) scale

C) percentage

D) rate

	
64.   	She cut her hair short and tried to ________ herself as a man.	
      	A) decorate

B) disguise

C) fabricate

D) fake

	
65.   	It is entirely ________ for us to fulfill the task ahead of schedule because of our application of the most advanced equipment all over the world.	
      	A) capable

B) able

C) possible

D) probable

	
66.   	The spy will die ________ he tells the enemies what they want to know.	
      	A) before

B) because

C) so

D) but

	
67.   	_________ is predicted by some newspapers, there will be substantial tax-cut on imported cars in the coming years.	
      	A) Which

B) That

C) It

D) As

	
68.   	The arrival of five unexpected guests threw everything into ________.	
      	A) confusion

B) conflict

C) concrete

D) contrast

	
69.   	Many ______ searchers have concentrated their efforts on detecting radio signals of alien civilizations.	
      	A) Venusian

B) extraterrestrial

C) cosmic

D) earthen

	
70.   	________ we'll go depends on the weather.	
      	A) Whether

B) If

C) That

D) What

	
71.   	His friend suggested that he should ________ advice from the legal department.	
      	A) seek for

B) seek after

C) seek

D) seek out

	
72.   	______ he works hard, I don't mind when he finishes the experiment.	
      	A) As soon as

B) As well as

C) So far as

D) So long as

	
73.   	The thief tried to open the locked door but _______.	
      	A) in no way

B) in vain

C) without effect

D) at a loss

	
74.   	One of the many ______ of foreign travel is learning how to cope with the unexpected.	
      	A) services

B) offers

C) interests

D) benefits

	
75.   	In no way ______ described as a liberal.	
      	A) President Bush could have been

B) could President Bush have been

C) could have President Bush been

D) could have been President Bush

	
76.   	This kind of analysis ______ the problem to its simplest form.	
      	A) breaks

B) displaces

C) decreases

D) reduces

	
77.   	You see the lightening ______ it happens, but you hear the thunder later.	
      	A) at an instant

B) for the instant

C) the instant

D) an instant

	
78.   	The music aroused an ________ feeling of homesickness in him.	
      	A) intrinsic

B) intentional

C) intermittent

D) intense

	
79.   	They're going to build a big office block on that ________ piece of land.	
      	A) void

B) vacant

C) blank

D) shallow

	
80.   	We picked up the story again at the point ________ John lost his job at the newspaper agency.	
      	A) where

B) when

C) that

D) which

	
81.   	His appearance has changed a lot, and you ________ recognize him.	
      	A) may well

B) may well not

C) may not well

D) may as well

	
