# Supplementary material for: The impact of the flipped classroom on the motivation and academic performance of Chinese college English learners
Source: PLoS One. 2025 May 2;20(5):e0322094. doi: 10.1371/journal.pone.0322094 (PMC12047774; doi:10.1371/journal.pone.0322094)
Supplement: S1 File — (ZIP) [file pone.0322094.s001.zip › S1/pretest for English proficiency.rtf]

	

Part ¢ñ   Listening Comprehension 

Section A
Directions: In this section, you will hear several conversations. At the end of each conversation, one or more questions will be asked about what was said. Both the conversations and the questions will be spoken only once. After each question there will be a pause. During the pause, you must read the four choices marked A), B), C) and D), and decide which is the best answer.

1.

	A) 35 cents.

B) 3 cents.

C) 3 dollars.

D) 1 dollars.

	
2.

	A) She doesn't need a new raincoat or umbrella.

B) It will probably not rain tomorrow.

C) She doesn't know what the weather will be like tomorrow.

D) She doesn't know where the man put his raincoat or umbrella.

	
3.

	A) Alan won't come.

B) Alan will come at 9.

C) Sally is often late.

D) Sally will come at 8.

	
4.

	A) Go to the movies with the man.

B) Cook dinner with Jerry.

C) Eat at her uncle's.

D) Take her uncle to the movies.

	
5.

	A) In a supermarket.

B) In a department store.

C) In a drugstore.

D) In a car repair shop.

	
6.

	A) If the man loves his job in the company.

B) How the man feels about the news.

C) If the man is going to lose his job.

D) Whether the man believes the news or not.

	
7.

	A) It's made from an old recipe.

B) It's in the refrigerator.

C) It has a variety of ingredients.

D) It needs to be heated up.

	
8.

	A) In a hotel.

B) In a restaurant.

C) In a cafeteria.

D) In a pizza hut.

	

Questions 9 to 12 are based on the conversation you have just heard.

9.

	A) About the film they saw last night.

B) About the advantages and disadvantages of science inventions.

C) About flying cars.

D) About computers.

	
10.

	A) The computers go crazy.

B) Some mad scientists want to profit from their inventions.

C) Nuclear weapons and cars cause pollution and medicine has side effects.

D) Some unpredictable disasters.

	
11.

	A) Scientists and technologists invent to change people¬ðs life.

B) Scientists and technologists are better at solving problems.

C) Scientists and technologists have even better or wilder imagination.

D) Scientists and technologists create more terrible side effects.

	
12.

	A) A more convenient, more comfortable and longer life.

B) Computer and other electronic devices.

C) Internet as a useful tool for people far away to communicate.

D) Amazing flying cars and other transportation means.

	

Questions 13 to 15 are based on the conversation you have just heard.

13.   	Why did the man call the City University?	
      	A) He wanted to interview a famous professor there.

B) He wanted to consult somebody about hydrogen engine.

C) He wanted to write a story about a professor.

D) He wanted to write a story about the university.

	
14.   	What does HEF refer to?	
      	A) Hydrogen Engine Facility.

B) Hydrogen Engine Faculty.

C) Hydrogen Engineering Faculty.

D) Hydrogen Engineering Facility.

	
15.   	Why did the woman suggest the man to come earlier than 5 p.m.?	
      	A) Because most people would make an appointment before 5 p.m.

B) Because they have a date then.

C) Because they would be off duty then.

D) Because they have a meeting at 5 p.m.

	

Section B
Directions: In this section, you will hear several short passages. At the end of each passage, you will hear some questions. Both the passage and the questions will be spoken only once. After you hear a question, you must choose the best answer from the four choices marked A), B), C) and D).

Passage One
Questions 16 to 19 are based on the passage you have just heard.

16.

	A) Germany.

B) Japan.

C) The US.

D) The UK.

	
17.

	A) By doing odd jobs at weekends.

B) By working long hours every day.

C) By putting in more hours each week.

D) By taking shorter vacations each year.

	
18.

	A) To combat competition and raise productivity.

B) To provide them with more job opportunities.

C) To help them maintain their living standard.

D) To prevent them from holding a second job.

	
19.

	A) Change their jobs.

B) Earn more money.

C) Reduce their working hours.

D) Strengthen the government's role.

	

Passage Two
Questions 20 to 22 are based on the passage you have just heard.

20.

	A) Football games announcers talk too much.

B) Make sure you hear yourself talk.

C) Words are sometimes offensive.

D) Effective communication is brief.

	
21.

	A) They lack confidence.

B) They tend to be sociable.

C) They are effective in establishing relations.

D) They are not careful.

	
22.

	A) Some of them are useless.

B) Some of them are definitely offensive.

C) You waste some time.

D) You leave an impression that you are wordy.

	

Passage Three
Questions 23 to 26 are based on the passage you have just heard.

23.

	A) On Tuesday.

B) On Wednesday.

C) On Thursday.

D) On Friday.

	
24.

	A) 20%.

B) 30%.

C) 50%.

D) 100%.

	
25.

	A) Thursday this week.

B) Thursday next week.

C) Tuesday this week.

D) Tuesday next week.

	
26.

	A) During the first week of class.

B) During the mid-term week.

C) On the last day of class.

D) On the last day of exam week.

	


Part ¢ò   Reading Comprehension 

Section A
Directions: There are several passages in this section. Each passage is followed by some questions or unfinished statements. For each of them there are four choices marked A), B), C) and D). You should decide on the best choice.

Passage One
Questions 27 to 31 are based on the following passage.

       It is, everyone agrees, a huge task that the child performs when he learns to speak, and the fact that he does so in so short a period of time challenges explanation.
        Language learning begins with listening. Individual children vary greatly in the amount of listening they do before they start speaking, and late starters are often long listeners. Most children will "obey" spoken instructions some time before they can speak, though the word obey is hardly accurate as a description of the eager and delighted cooperation usually shown by children. Before they can speak, many children will also ask questions by gesture and by making questioning noises.
        Any attempt to trace the development from the noises babies make to their first spoken words leads to considerable difficulties. It is agreed that they enjoy making noises, and that during the first few months one or two noises sort themselves out as particularly indicative of delight, distress, sociability, and so on. But since these cannot be said to show the baby's intention to communicate, they can hardly be regarded as early forms of language. It is agreed, too, that from about three months they play with sounds for enjoyment, and that by six months they are able to add new sounds to their repertoire (ÄÜ·¢³öµÄÈ«²¿ÉùÒô). This self-imitation leads on to deliberate (ÓÐÒâÊ¶µÄ) imitation of sounds made or words spoken to them by other people. The problem then arises as to the point at which one can say that these imitations can be considered as speech.


27.   	By " ... challenges explanation" (Paragraph 1) the author means that ______.	
      	A) no explanation is necessary for such an obvious phenomenon ª¤

B) no explanation has been made up to now

C) it's no easy job to provide an adequate explanation

D) it's high time that an explanation was provided

	
28.   	The third paragraph is mainly about _____.	
      	A) the development of babies' early forms of language

B) the difficulties of babies in learning to speak

C) babies' strong desire to communicate

D) babies' intention to communicate

	
29.   	The author's purpose in writing the second paragraph is to show that children _______.	
      	A) usually obey without asking questions

B) are passive in the process of learning to speak

C) are born cooperative

D) learn to speak by listening

	
30.   	From the passage we learn that ______.	
      	A) early starters can learn to speak within only six monthsª¤

B) children show a strong desire to communicate by making noises

C) imitation plays an important role in learning to speak

D) children have various difficulties in learning to speakª¤

	
31.   	The best title for this passage would be ______.	
      	A) How Babies Learn to Speak

B) Early Forms of Language

C) A Huge Task for Children

D) Noise Making and Language Learning

	

Passage Two
Questions 32 to 36 are based on the following passage.

       A material which usually keeps animals and people warm is providing an oil clean-up service for the environment. Wool from sheep proved to be an effective "sponge" to soak up oil from the waters of the Persian Gulf after the Gulf War in 1991. The absorbent qualities of wool were used to good effect in wool poles which swept across the surface of the water.
        Now the same absorbent qualities are being used by railways in the Netherlands. Wool mats produced by Rhanex Wools of New Zealand have been laid between the rails at eight major Dutch railway stations to soak up diesel oil dropping from locomotives. Dutch authorities were worried that diesel oil was seeping (ÉøÈë) through the ground into the watertable, which is used for farm irrigation and to supply drinking water. The mats can be regularly squeezed to extract the diesel oil from them, and then reused.
        Similar wool mats are being used in inner city car parking lots in Sydney, Australia, to soak up oil dropping from parked cars. One parking lot owner believed that the value of his land could be reduced by oil pollution.
        The environmental friendly qualities of wool are also being put to good use to protect wool itself. High value Merino sheep in Australia and Canada are wearing wool covers to protect their fleece (ÑòÃ«).


32.   	Which of the following is NOT one of wool's advantages?	
      	A) It has absorbent quality.

B) It could be squeezed and reused.

C) It can be used to avoid pollution.

D) It could increase the price of land.

	
33.   	It can be inferred that wool first proved very useful in absorbing oil ___.	
      	A) in ancient time

B) in the Gulf War

C) after the Gulf War

D) before the Gulf War

	
34.   	Which country used wool to prevent oil seeping into earth?	
      	A) America.

B) Iraq.

C) New Zealand.

D) The Netherlands.

	
35.   	Why are wool mats being used in parking lots in Sydney?	
      	A) To prevent the cars from turning over.

B) To absorb oil leaking from the parked cars.

C) To attract more drivers to park their cars.

D) To ask for a higher price when the lands are sold.

	
36.   	Which function of wool is NOT mentioned in the passage?	
      	A) Storing up oil.

B) Making clothes.

C) Absorbing oil.

D) Protecting sheep.

	

Passage Three
Questions 37 to 41 are based on the following passage.

       At one point along an open highway, I came to a crossroads with a traffic light. I was alone on the road by now, but as I approached the light, it turned red, and I braked to a halt. I looked left, right, and behind me. Nothing. Not a car, no suggestion of headlights, but there I sat, waiting for the light to change, the only human being for at least a mile in any direction.
        I started wondering why I refused to run the light. I was not afraid of being arrested, because there was obviously no cop anywhere around and there certainly would have been no danger in going through it.
        Much later that night, after I'd met with a group in Lewisburg and had climbed into bed near midnight, the question of why I'd stopped for that light came back to me. I think I stopped because it's part of a contract we all have with each other. It's not only the law, but it's an agreement we have, and we trust each other to honor it: We don't go through red lights. Like most of us, I'm more apt to be restrained from doing something bad by the social convention that disapproves of it than by any law against it.
        It's amazing that we ever trust each other to do the right thing, isn't it? And we do, too. Trust is our first inclination. We have to make a deliberate decision to mistrust someone or to be suspicious or skeptical.


37.   	Why didn't the author run the light?	
      	A) Because he was afraid of being caught by the police.

B) Because he was in a hurry.

C) Because he knew it was wrong.

D) Because it was dangerous to do so.

	
38.   	When the author stopped there was ____.	
      	A) another car

B) only his car

C) a police car

D) many cars

	
39.   	The author thought about his stopping for that light ____.	
      	A) on his way to Lewisburg

B) upon arrival at Lewisburg

C) at the meeting

D) in the bed late that day

	
40.   	In the author's opinion, people don't go through red lights ____.	
      	A) for it is against the law

B) for fear of being knocked down

C) in presence of policemen

D) because of social conventions

	
41.   	"Skeptical" in the last paragraph means ____.	
      	A) cautious

B) alert

C) doubtful

D) unfriendly

	

Section B
Directions: In this section, there is a passage with several blanks. You are required to select one word for each blank from a list of choices given in a word bank following the passage. Read the passage through carefully before making your choices. Each choice in the bank is identified by a letter. You may not use any of the words in the bank more than once.

Questions 42-51 are based on the following passage.

       In the 1820s Coffin moved west to Newport (now Fountain City), Indiana, where he opened a store. Word spread that fleeing slaves could always find   42   at the Coffin home. At times he   43   as many as 17 fugitives at once, and he kept a team and wagon ready to convey them on the next   44   of their journey. Eventually three principal routes   45   at the Coffin house, which came to be the Grand Central Terminal of the Underground Railroad. 
        For his efforts, Coffin received frequent   46   and warnings that his store and home would be burned. Nearly every conductor faced similar risks —   47   . In the North, a magistrate might have imposed a   48   or a brief jail sentence for aiding those escaping. In the Southern states, whites were   49   to months or even years in jail. One   50   Methodist minister, Calvin Fairbank, was imprisoned for more than 17 years in Kentucky, where he   51   his beatings: 35,105 stripes with the whip.

A) conformed
B) converged
C) fine
D) sharply

E) death threats
F) prejudiced
G) courageous
H) kept a log of

I) crippled
J) leg
K) or worse
L) refuge

M) identity
N) sheltered
O) sentenced


	


Part ¢ó   Vocabulary and Grammar 

Directions: There are a number of incomplete sentences in this part. For each sentence there are four choices marked A), B), C) and D). Choose the ONE that best completes the sentence.

52.   	He  went to great lengths  to keep Dick from getting a date with Mary.	
      	A) went so far as

B) went a long way

C) tried every possible means

D) took measures

	
53.   	Her chief attraction  rests in  her character, not her looks.	
      	A) comes from

B) aims at

C) reaches for

D) results in

	
54.   	Sand had ______ at the mouth of the river and formed a bank which boats could not pass.	
      	A) collected

B) accumulated

C) assembled

D) added

	
55.   	The old man prefers the ______ life to the life in the city as he enjoys little blossoms and little birds when the spring comes.	
      	A) diligent

B) fugitive

C) rural

D) civilized

	
56.   	A chemical has been discovered that may be the key to ______ the mysteries of Parkinson's disease.	
      	A) overseeing

B) oversee

C) unlocking

D) unlock

	
57.   	He usually feels ill at ease ________.	
      	A) in memory of her

B) in her way

C) in her company

D) in favor of her

	
58.   	She worked hard at her task before she felt sure that the results would ________ her long effort.	
      	A) justify

B) testify

C) rectify

D) verify

	
59.   	________ of the buses will stop at the Peace Hotel, and you can take either of them to get there.	
      	A) Each

B) Any

C) Either

D) Both

	
60.   	Large windows can give an ________ of spaciousness.	
      	A) impression

B) effect

C) reaction

D) observation

	
61.   	They rose one after ________ and walked out.	
      	A) the other

B) each other

C) others

D) another

	
62.   	An author must not be too ________ to criticism.	
      	A) sensitive

B) sensible

C) senseless

D) insensible

	
63.   	"Bob certainly has a low opinion of Sue." — -"It can't be any worse than ________ of him."	
      	A) her

B) hers

C) she

D) she does

	
64.   	I intended ____________ the matter with you, but I had some guests then.	
      	A) discuss

B) having discussed

C) to have discussed

D) discussing

	
65.   	The president promised to keep all the board members _____ of how the negotiations were going on.	
      	A) illustrated

B) proved

C) improved

D) informed

	
66.   	On hearing the good news, we had been ________ with excitement till he came off the platform with a red flower on his chest.	
      	A) shivering

B) trembling

C) shaking

D) quaking

	
67.   	There is still ________ hope of our getting there in time. Let's hurry.	
      	A) little

B) few

C) a little

D) a few

	
68.   	The millions of calculations involved, had they been done by hand, all practical value _______ by the time they were finished.	
      	A) had lost

B) would have lost

C) would lose

D) should have lost

	
69.   	The Roman _____ is computed from the date when Roman was supposedly founded.	
      	A) period

B) epoch

C) era

D) age

	
70.   	Lafayette Hotel ______ by international celebrities drawn to its French food and service.	
      	A) is haunted

B) visited

C) to be frequented

D) is patronized

	
71.   	Mary is being ________.	
      	A) unhappy

B) helpful

C) delighted

D) beautiful

	
72.   	________ the dog was the first animal to be domesticated is generally agreed upon by authorities in the field.	
      	A) Until

B) It was

C) What

D) That

	
73.   	Only when capitalism has been ________ will it be possible to _________ poverty, unemployment and war.	
      	A) abolished ... abolish

B) abandoned ... abate

C) annihilated ... ban

D) extinguished ... extinguish

	
74.   	When I caught him ______ me, I stopped buying things there and started dealing with another shop.	
      	A) cheating

B) cheat

C) to cheat

D) to be cheating

	
75.   	I had been sitting in my seat for at least two hours, waiting ________.	
      	A) the train to start

B) for the train starting

C) for the train to start

D) for the train start

	
76.   	The librarian put me in the way of a lot of new material __________ the subject of my report.	
      	A) in

B) with

C) on

D) at

	
77.   	In recent years much more emphasis has been put __________ developing the sutdents' productive skills.	
      	A) over

B) onto

C) in

D) on

	
78.   	He is ______ of the slightest noise, and can not allow himself to be disturbed by even most necessary questionings.	
      	A) guilty

B) desirable

C) frightful

D) impatient

	
79.   	The coach was ________ the point of giving up the game when our team scored two points.	
      	A) on

B) by

C) in

D) to

	
80.   	Jack's parents are still wondering who suggested ________ in the car race.	
      	A) his taking part

B) his take part

C) for his taking part

D) him to take part

	
81.   	Do you like any of the music _________ you've listened?	
      	A) for which

B) that

C) to which

D) which

	
