# Supplementary material for: The impact of the flipped classroom on the motivation and academic performance of Chinese college English learners
Source: PLoS One. 2025 May 2;20(5):e0322094. doi: 10.1371/journal.pone.0322094 (PMC12047774; doi:10.1371/journal.pone.0322094)
Supplement: S1 File — (ZIP) [file pone.0322094.s001.zip › S1/Reliability test of English learning motivation scale before exploratory factor analysis.docx]

**Reliability test of English learning motivation scale before exploratory factor analysis**

**Scale: ALL VARIABLES**

| **Case Processing Summary** | | | |
| --- | --- | --- | --- |
|  | | N | % |
| Cases | Valid | 100 | 100.0 |
|  | Excluded^a^ | 0 | 0.0 |
|  | Total | 100 | 100.0 |
| a. Listwise deletion based on all variables in the procedure. | | | |

| **Reliability Statistics** | |
| --- | --- |
| Cronbach's Alpha | N of Items |
| .935 | 16 |

| **Item-Total Statistics** | | | | |
| --- | --- | --- | --- | --- |
|  | Scale Mean if Item Deleted | Scale Variance if Item Deleted | Corrected Item-Total Correlation | Cronbach's Alpha if Item Deleted |
| Q3 | 52.33 | 116.526 | .661 | .931 |
| Q8 | 52.21 | 113.440 | .717 | .929 |
| Q10 | 52.03 | 112.837 | .782 | .928 |
| Q11 | 52.28 | 115.456 | .693 | .930 |
| Q12 | 52.31 | 117.630 | .595 | .932 |
| Q13 | 52.41 | 116.164 | .703 | .930 |
| Q1 | 52.52 | 116.091 | .604 | .932 |
| Q2 | 52.31 | 115.166 | .679 | .930 |
| Q4 | 52.27 | 115.936 | .612 | .932 |
| Q5 | 52.30 | 115.121 | .703 | .930 |
| Q6 | 52.04 | 115.796 | .626 | .932 |
| Q7 | 52.10 | 114.697 | .687 | .930 |
| Q9 | 52.16 | 115.247 | .682 | .930 |
| Q14 | 51.96 | 116.443 | .646 | .931 |
| Q15 | 51.83 | 118.223 | .632 | .931 |
| Q16 | 51.84 | 118.499 | .597 | .932 |
|  |  |  |  |  |

| **Scale Statistics** | | | |
| --- | --- | --- | --- |
| Mean | Variance | Std. Deviation | N of Items |
| 55.66 | 131.217 | 11.455 | 16 |
