# Supplementary material for: The impact of the flipped classroom on the motivation and academic performance of Chinese college English learners
Source: PLoS One. 2025 May 2;20(5):e0322094. doi: 10.1371/journal.pone.0322094 (PMC12047774; doi:10.1371/journal.pone.0322094)
Supplement: S1 File — (ZIP) [file pone.0322094.s001.zip › S1/Respective reliability tests of all four factors after exploratory factor analysis.docx]

**Reliability test of factor 1—Autonomous Learning Motivation**

| **Case Processing Summary** | | | | | |  |
| --- | --- | --- | --- | --- | --- | --- |
|  | | | N | | % |  |
| Cases | Valid | | 100 | | 100.0 |  |
|  | Excluded^a^ | | 0 | | 0.0 |  |
|  | Total | | 100 | | 100.0 |  |
| a. Listwise deletion based on all variables in the procedure. | | | | | |  |
|  |  | | |  |  |  |
|  |  | | |  |  |  |
| **Reliability Statistics** | | | |  |  |  |
| Cronbach's Alpha | | N of Items | |  |  |  |
| .910 | | 6 | |  |  |  |
|  | |  | |  |  |  |
|  | |  | |  |  |  |
| **Item-Total Statistics** | | | | | | |
|  | Scale Mean if Item Deleted | | Scale Variance if Item Deleted | | Corrected Item-Total Correlation | Cronbach's Alpha if Item Deleted |
| Q3 | 17.06 | | 17.996 | | .723 | .897 |
| Q8 | 16.94 | | 16.623 | | .796 | .887 |
| Q10 | 16.76 | | 16.891 | | .803 | .885 |
| Q11 | 17.01 | | 17.586 | | .754 | .893 |
| Q12 | 17.04 | | 18.099 | | .696 | .901 |
| Q13 | 17.14 | | 18.202 | | .722 | .897 |

**Reliability test of factor 2—Integrative Motivation**

| **Case Processing Summary** | | | |  |
| --- | --- | --- | --- | --- |
|  | | N | % |  |
| Cases | Valid | 100 | 100.0 |  |
|  | Excluded^a^ | 0 | 0.0 |  |
|  | Total | 100 | 100.0 |  |
| a. Listwise deletion based on all variables in the procedure. | | | |  |
|  |  |  |  |  |
| **Reliability Statistics** | |  |  |  |
| Cronbach's Alpha | N of Items |  |  |  |
| .909 | 3 |  |  |  |
|  |  |  |  |  |
| **Item-Total Statistics** | | | | |
|  | Scale Mean if Item Deleted | Scale Variance if Item Deleted | Corrected Item-Total Correlation | Cronbach's Alpha if Item Deleted |
| Q1 | 6.74 | 3.912 | .817 | .871 |
| Q2 | 6.53 | 4.110 | .806 | .880 |
| Q4 | 6.49 | 3.869 | .833 | .857 |

**Reliability test of factor 3—Instrumental Motivation**

| **Case Processing Summary** | | | |  |
| --- | --- | --- | --- | --- |
|  | | N | % |  |
| Cases | Valid | 100 | 100.0 |  |
|  | Excluded^a^ | 0 | 0.0 |  |
|  | Total | 100 | 100.0 |  |
| a. Listwise deletion based on all variables in the procedure. | | | |  |
|  |  |  |  |  |
| **Reliability Statistics** | |  |  |  |
| Cronbach's Alpha | N of Items |  |  |  |
| .914 | 4 |  |  |  |
|  |  |  |  |  |
| **Item-Total Statistics** | | | | |
|  | Scale Mean if Item Deleted | Scale Variance if Item Deleted | Corrected Item-Total Correlation | Cronbach's Alpha if Item Deleted |
| Q5 | 10.68 | 8.018 | .801 | .889 |
| Q6 | 10.42 | 7.640 | .816 | .884 |
| Q7 | 10.48 | 7.828 | .790 | .893 |
| Q9 | 10.54 | 7.887 | .807 | .887 |

**Reliability test of factor 4—Instrinsic Motivation**

| **Case Processing Summary** | | | |  |
| --- | --- | --- | --- | --- |
|  | | N | % |  |
| Cases | Valid | 100 | 100.0 |  |
|  | Excluded^a^ | 0 | 0.0 |  |
|  | Total | 100 | 100.0 |  |
| a. Listwise deletion based on all variables in the procedure. | | | |  |
|  |  |  |  |  |
| **Reliability Statistics** | |  |  |  |
| Cronbach's Alpha | N of Items |  |  |  |
| .865 | 3 |  |  |  |
|  |  |  |  |  |
| **Item-Total Statistics** | | | | |
|  | Scale Mean if Item Deleted | Scale Variance if Item Deleted | Corrected Item-Total Correlation | Cronbach's Alpha if Item Deleted |
| Q14 | 7.65 | 2.654 | .780 | .775 |
| Q15 | 7.52 | 3.141 | .705 | .844 |
| Q16 | 7.53 | 2.959 | .749 | .804 |
