# Supplementary material for: The impact of the flipped classroom on the motivation and academic performance of Chinese college English learners
Source: PLoS One. 2025 May 2;20(5):e0322094. doi: 10.1371/journal.pone.0322094 (PMC12047774; doi:10.1371/journal.pone.0322094)

**Supplementary Information**

**Part 1. Reliability test of English learning motivation scale before exploratory factor analysis (page: 2)**

**Part 2. Exploratory Factor Analysis of English Learning Motivation Scale (page: 3-5)**

**Part 3. Respective reliability tests of all four factors after exploratory factor analysis (page: 6-8)**

**Part 4. Confirmatory Factor Analysis of the Model—Autonomous Learning Motivation (page: 9-13)**

**Part 5. Confirmatory Factor Analysis of the Model—Instrumental Motivation (page:14-17)**

**Part 6. Confirmatory Factor Analysis of English Learning Motivation Model (page:18-25)**

**Part 7. data sets for research question1 (page:26-37)**

**Part 8. data sets for research question2 (page:38-41)**

**Part 9. data sets for research question3 (page:42-46)**

**Part 1. Reliability test of English learning motivation scale before exploratory factor analysis**

**Scale: ALL VARIABLES**

| **Case Processing Summary** | | | |
| --- | --- | --- | --- |
|  | | N | % |
| Cases | Valid | 100 | 100.0 |
|  | Excluded^a^ | 0 | 0.0 |
|  | Total | 100 | 100.0 |
| a. Listwise deletion based on all variables in the procedure. | | | |

| **Reliability Statistics** | |
| --- | --- |
| Cronbach's Alpha | N of Items |
| .935 | 16 |

| **Item-Total Statistics** | | | | |
| --- | --- | --- | --- | --- |
|  | Scale Mean if Item Deleted | Scale Variance if Item Deleted | Corrected Item-Total Correlation | Cronbach's Alpha if Item Deleted |
| Q3 | 52.33 | 116.526 | .661 | .931 |
| Q8 | 52.21 | 113.440 | .717 | .929 |
| Q10 | 52.03 | 112.837 | .782 | .928 |
| Q11 | 52.28 | 115.456 | .693 | .930 |
| Q12 | 52.31 | 117.630 | .595 | .932 |
| Q13 | 52.41 | 116.164 | .703 | .930 |
| Q1 | 52.52 | 116.091 | .604 | .932 |
| Q2 | 52.31 | 115.166 | .679 | .930 |
| Q4 | 52.27 | 115.936 | .612 | .932 |
| Q5 | 52.30 | 115.121 | .703 | .930 |
| Q6 | 52.04 | 115.796 | .626 | .932 |
| Q7 | 52.10 | 114.697 | .687 | .930 |
| Q9 | 52.16 | 115.247 | .682 | .930 |
| Q14 | 51.96 | 116.443 | .646 | .931 |
| Q15 | 51.83 | 118.223 | .632 | .931 |
| Q16 | 51.84 | 118.499 | .597 | .932 |
|  |  |  |  |  |

| **Scale Statistics** | | | |
| --- | --- | --- | --- |
| Mean | Variance | Std. Deviation | N of Items |
| 55.66 | 131.217 | 11.455 | 16 |

**Part 2. Exploratory Factor Analysis of English Learning Motivation Scale**

| **KMO and Bartlett's Test** | | | | | |
| --- | --- | --- | --- | --- | --- |
| Kaiser-Meyer-Olkin Measure of Sampling Adequacy. | | | | | .901 |
| Bartlett's Test of Sphericity | | | Approx. Chi-Square | | 1158.560 |
|  |  |  | df | | 120 |
|  |  |  | Sig. | | .000 |
| **Communalities** | | | |  |  |
|  | Initial | Extraction | |  |  |
| Q3 | 1.000 | .654 | |  |  |
| Q8 | 1.000 | .786 | |  |  |
| Q10 | 1.000 | .761 | |  |  |
| Q11 | 1.000 | .688 | |  |  |
| Q12 | 1.000 | .718 | |  |  |
| Q13 | 1.000 | .668 | |  |  |
| Q1 | 1.000 | .865 | |  |  |
| Q2 | 1.000 | .823 | |  |  |
| Q4 | 1.000 | .864 | |  |  |
| Q5 | 1.000 | .793 | |  |  |
| Q6 | 1.000 | .833 | |  |  |
| Q7 | 1.000 | .775 | |  |  |
| Q9 | 1.000 | .810 | |  |  |
| Q14 | 1.000 | .817 | |  |  |
| Q15 | 1.000 | .709 | |  |  |
| Q16 | 1.000 | .816 | |  |  |
| Extraction Method: Principal Component Analysis. | | | |  |  |

**
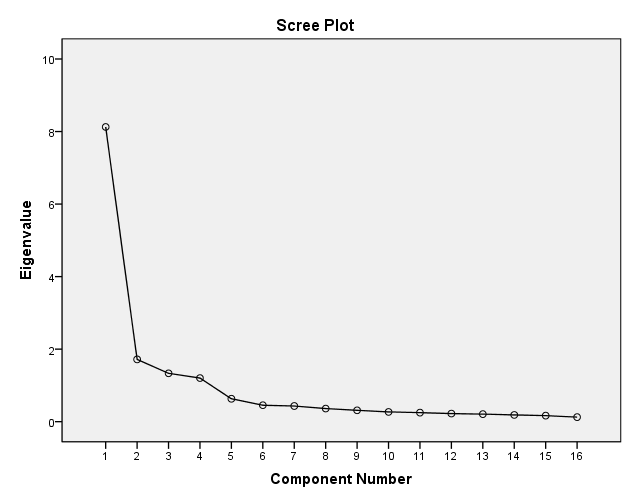
**

| **Total Variance Explained** | | | | | | | | | |
| --- | --- | --- | --- | --- | --- | --- | --- | --- | --- |
| Component | Initial Eigenvalues | | | Extraction Sums of Squared Loadings | | | Rotation Sums of Squared Loadings | | |
|  | Total | % of Variance | Cumulative % | Total | % of Variance | Cumulative % | Total | % of Variance | Cumulative % |
| 1 | 8.126 | 50.788 | 50.788 | 8.126 | 50.788 | 50.788 | 3.975 | 24.847 | 24.847 |
| 2 | 1.717 | 10.733 | 61.521 | 1.717 | 10.733 | 61.521 | 3.194 | 19.963 | 44.810 |
| 3 | 1.334 | 8.335 | 69.857 | 1.334 | 8.335 | 69.857 | 2.641 | 16.505 | 61.315 |
| 4 | 1.203 | 7.519 | 77.375 | 1.203 | 7.519 | 77.375 | 2.570 | 16.060 | 77.375 |
| 5 | .632 | 3.949 | 81.324 |  |  |  |  |  |  |
| 6 | .454 | 2.836 | 84.160 |  |  |  |  |  |  |
| 7 | .432 | 2.699 | 86.859 |  |  |  |  |  |  |
| 8 | .361 | 2.259 | 89.117 |  |  |  |  |  |  |
| 9 | .314 | 1.960 | 91.077 |  |  |  |  |  |  |
| 10 | .269 | 1.682 | 92.759 |  |  |  |  |  |  |
| 11 | .249 | 1.556 | 94.315 |  |  |  |  |  |  |
| 12 | .223 | 1.393 | 95.708 |  |  |  |  |  |  |
| 13 | .209 | 1.304 | 97.012 |  |  |  |  |  |  |
| 14 | .186 | 1.165 | 98.177 |  |  |  |  |  |  |
| 15 | .166 | 1.040 | 99.217 |  |  |  |  |  |  |
| 16 | .125 | .783 | 100.000 |  |  |  |  |  |  |
| Extraction Method: Principal Component Analysis. | | | | | | | | | |

| **Rotated Component Matrix^a^** | | | | |
| --- | --- | --- | --- | --- |
|  | Component | | | |
|  | 1 | 2 | 3 | 4 |
| Q12 | .794 |  |  |  |
| Q8 | .789 |  |  |  |
| Q11 | .745 |  |  |  |
| Q3 | .741 |  |  |  |
| Q10 | .724 |  |  |  |
| Q13 | .699 |  |  |  |
| Q6 |  | .868 |  |  |
| Q9 |  | .823 |  |  |
| Q5 |  | .793 |  |  |
| Q7 |  | .767 |  |  |
| Q1 |  |  | .877 |  |
| Q4 |  |  | .870 |  |
| Q2 |  |  | .802 |  |
| Q16 |  |  |  | .841 |
| Q14 |  |  |  | .818 |
| Q15 |  |  |  | .738 |
| Extraction Method: Principal Component Analysis.   Rotation Method: Varimax with Kaiser Normalization. | | | | |
| a. Rotation converged in 5 iterations. | | | | |

| **Component Transformation Matrix** | | | | |
| --- | --- | --- | --- | --- |
| Component | 1 | 2 | 3 | 4 |
| 1 | .615 | .505 | .422 | .434 |
| 2 | -.434 | .663 | -.508 | .338 |
| 3 | -.657 | .073 | .740 | .126 |
| 4 | -.046 | -.548 | -.127 | .826 |
| Extraction Method: Principal Component Analysis.   Rotation Method: Varimax with Kaiser Normalization. | | | | |

**Part 3. Respective reliability tests of all four factors after exploratory factor analysis**

**Reliability test of factor 1—Autonomous Learning Motivation**

| **Case Processing Summary** | | | | | |  |
| --- | --- | --- | --- | --- | --- | --- |
|  | | | N | | % |  |
| Cases | Valid | | 100 | | 100.0 |  |
|  | Excluded^a^ | | 0 | | 0.0 |  |
|  | Total | | 100 | | 100.0 |  |
| a. Listwise deletion based on all variables in the procedure. | | | | | |  |
|  |  | | |  |  |  |
|  |  | | |  |  |  |
| **Reliability Statistics** | | | |  |  |  |
| Cronbach's Alpha | | N of Items | |  |  |  |
| .910 | | 6 | |  |  |  |
|  | |  | |  |  |  |
|  | |  | |  |  |  |
| **Item-Total Statistics** | | | | | | |
|  | Scale Mean if Item Deleted | | Scale Variance if Item Deleted | | Corrected Item-Total Correlation | Cronbach's Alpha if Item Deleted |
| Q3 | 17.06 | | 17.996 | | .723 | .897 |
| Q8 | 16.94 | | 16.623 | | .796 | .887 |
| Q10 | 16.76 | | 16.891 | | .803 | .885 |
| Q11 | 17.01 | | 17.586 | | .754 | .893 |
| Q12 | 17.04 | | 18.099 | | .696 | .901 |
| Q13 | 17.14 | | 18.202 | | .722 | .897 |

**Reliability test of factor 2—Integrative Motivation**

| **Case Processing Summary** | | | |  |
| --- | --- | --- | --- | --- |
|  | | N | % |  |
| Cases | Valid | 100 | 100.0 |  |
|  | Excluded^a^ | 0 | 0.0 |  |
|  | Total | 100 | 100.0 |  |
| a. Listwise deletion based on all variables in the procedure. | | | |  |
|  |  |  |  |  |
| **Reliability Statistics** | |  |  |  |
| Cronbach's Alpha | N of Items |  |  |  |
| .909 | 3 |  |  |  |
|  |  |  |  |  |
| **Item-Total Statistics** | | | | |
|  | Scale Mean if Item Deleted | Scale Variance if Item Deleted | Corrected Item-Total Correlation | Cronbach's Alpha if Item Deleted |
| Q1 | 6.74 | 3.912 | .817 | .871 |
| Q2 | 6.53 | 4.110 | .806 | .880 |
| Q4 | 6.49 | 3.869 | .833 | .857 |

**Reliability test of factor 3—Instrumental Motivation**

| **Case Processing Summary** | | | |  |
| --- | --- | --- | --- | --- |
|  | | N | % |  |
| Cases | Valid | 100 | 100.0 |  |
|  | Excluded^a^ | 0 | 0.0 |  |
|  | Total | 100 | 100.0 |  |
| a. Listwise deletion based on all variables in the procedure. | | | |  |
|  |  |  |  |  |
| **Reliability Statistics** | |  |  |  |
| Cronbach's Alpha | N of Items |  |  |  |
| .914 | 4 |  |  |  |
|  |  |  |  |  |
|  |  |  |  |  |
| **Item-Total Statistics** | | | | |
|  | Scale Mean if Item Deleted | Scale Variance if Item Deleted | Corrected Item-Total Correlation | Cronbach's Alpha if Item Deleted |
| Q5 | 10.68 | 8.018 | .801 | .889 |
| Q6 | 10.42 | 7.640 | .816 | .884 |
| Q7 | 10.48 | 7.828 | .790 | .893 |
| Q9 | 10.54 | 7.887 | .807 | .887 |

**Reliability test of factor 4—Instrinsic Motivation**

| **Case Processing Summary** | | | |  |
| --- | --- | --- | --- | --- |
|  | | N | % |  |
| Cases | Valid | 100 | 100.0 |  |
|  | Excluded^a^ | 0 | 0.0 |  |
|  | Total | 100 | 100.0 |  |
| a. Listwise deletion based on all variables in the procedure. | | | |  |
|  |  |  |  |  |
| **Reliability Statistics** | |  |  |  |
| Cronbach's Alpha | N of Items |  |  |  |
| .865 | 3 |  |  |  |
|  |  |  |  |  |
| **Item-Total Statistics** | | | | |
|  | Scale Mean if Item Deleted | Scale Variance if Item Deleted | Corrected Item-Total Correlation | Cronbach's Alpha if Item Deleted |
| Q14 | 7.65 | 2.654 | .780 | .775 |
| Q15 | 7.52 | 3.141 | .705 | .844 |
| Q16 | 7.53 | 2.959 | .749 | .804 |

**Part 4. Confirmatory Factor Analysis of the Model—Autonomous Learning Motivation**

**
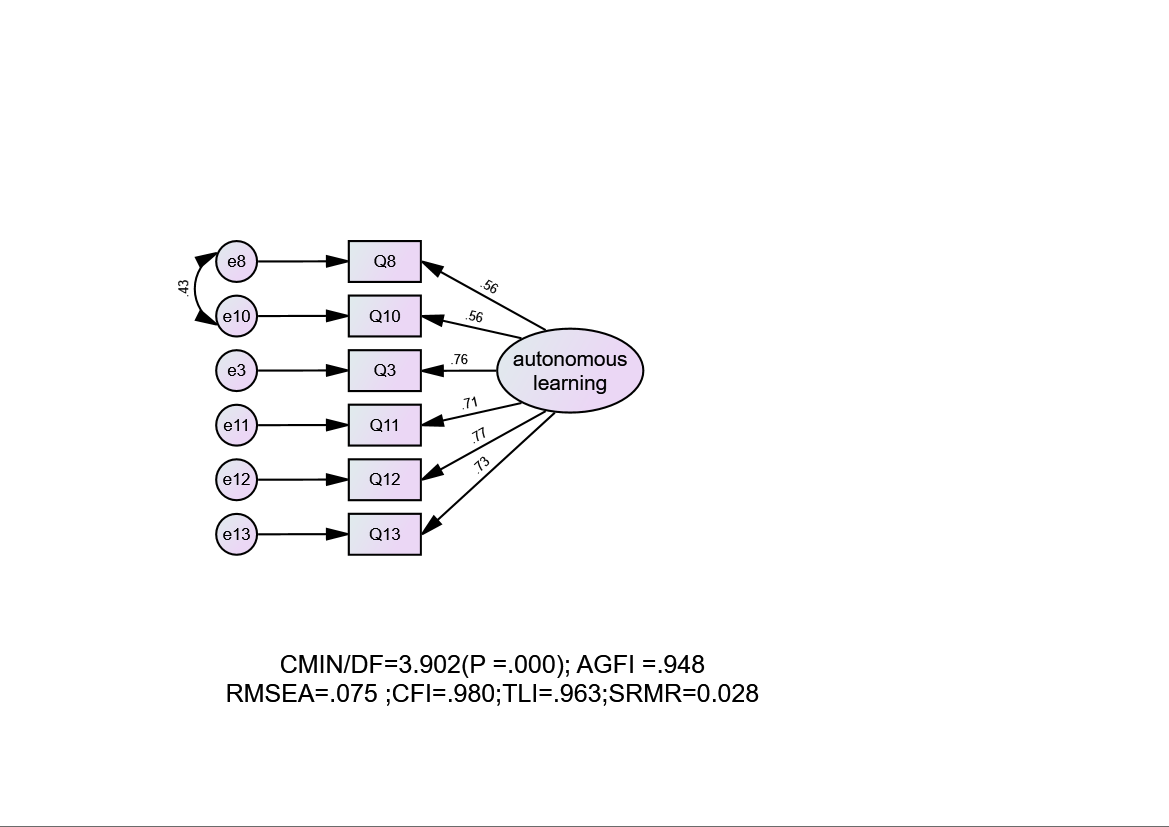
**

| **Estimates (Group number 1 - Default model)** | | | | | | | | | |  | | |  | | |  | | |  | |  | |  |  |
| --- | --- | --- | --- | --- | --- | --- | --- | --- | --- | --- | --- | --- | --- | --- | --- | --- | --- | --- | --- | --- | --- | --- | --- | --- |
|  | |  | | |  | |  | | |  | | |  | | |  | | |  | |  | |  |  |
| **Scalar Estimates (Group number 1 - Default model)** | | | | | | | | | | | | |  | | |  | | |  | |  | |  |  |
|  | |  | | |  | |  | | |  | | |  | | |  | | |  | |  | |  |  |
| **Maximum Likelihood Estimates** | | | | | | |  | | |  | | |  | | |  | | |  | |  | |  |  |
|  | |  | | |  | |  | | |  | | |  | | |  | | |  | |  | |  |  |
|  | | |  | | |  | |  | | |  | | |  | | |  |  |  |  |  |  |  |  |
| **Regression Weights: (Group number 1 - Default model)** | | | | | | | | | | | | | | |  | | |  | |  | |  |  |  |
|  |  | | |  | | | | |  | | |  | | |  | | |  | |  | |  |  |  |
|  |  | | |  | | | | | **Estimate** | | | **S.E.** | | | **C.R.** | | | **P** | | **Label** | |  |  |  |
| Q13 | <--- | | | autonomous_learning | | | | | 1 | | |  | | |  | | |  | |  | |  |  |  |
| Q12 | <--- | | | autonomous_learning | | | | | 1.196 | | | 0.076 | | | 15.668 | | | *** | |  | |  |  |  |
| Q11 | <--- | | | autonomous_learning | | | | | 1.013 | | | 0.07 | | | 14.53 | | | *** | |  | |  |  |  |
| Q3 | <--- | | | autonomous_learning | | | | | 1.096 | | | 0.071 | | | 15.536 | | | *** | |  | |  |  |  |
| Q10 | <--- | | | autonomous_learning | | | | | 0.844 | | | 0.073 | | | 11.547 | | | *** | |  | |  |  |  |
| Q8 | <--- | | | autonomous_learning | | | | | 0.905 | | | 0.078 | | | 11.54 | | | *** | |  | |  |  |  |

**Standardized Regression Weights: (Group number 1 - Default model)**

|  |  |  | **Estimate** |
| --- | --- | --- | --- |
| Q13 | <--- | autonomous_learning | .729 |
| Q12 | <--- | autonomous_learning | .771 |
| Q11 | <--- | autonomous_learning | .707 |
| Q3 | <--- | autonomous_learning | .763 |
| Q10 | <--- | autonomous_learning | .559 |
| Q8 | <--- | autonomous_learning | .559 |

**Covariances: (Group number 1 - Default model)**

|  | | |  |  | **Estimate** | | **S.E.** | | | **C.R.** | | **P** | | | **Label** |  |  |  |
| --- | --- | --- | --- | --- | --- | --- | --- | --- | --- | --- | --- | --- | --- | --- | --- | --- | --- | --- |
| e10 | | | <--> | e8 | .284 | | .036 | | | 7.946 | | *** | | |  |  |  |  |
| **Correlations: (Group number 1 - Default model)** | | | | | | | | | | | | | | |  |  |  |  |
|  | | | |  | |  | | | | | |  | | |  |  |  |  |
|  | | | |  | |  | | | | | | **Estimate** | | |  |  |  |  |
| e10 | | | | <--> | | e8 | | | | | | 0.426 | | |  |  |  |  |
| **Variances: (Group number 1 - Default model)** | | | | | | | | | | | |  | | |  |  |  |  |
|  | | | | | | | |  | |  | |  | | |  |  |  |  |
|  | | | | | | | |  | |  | | **Estimate** | | | **S.E.** | **C.R.** | **P** | **Label** |
| **autonomous_learning** | | | | | | | |  | |  | | 0.398 | | | 0.045 | 8.899 | *** |  |
| **e13** | | | | | | | |  | |  | | 0.35 | | | 0.028 | 12.64 | *** |  |
| **e12** | | | | | | | |  | |  | | 0.389 | | | 0.033 | 11.67 | *** |  |
| **e11** | | | | | | | |  | |  | | 0.408 | | | 0.031 | 13.05 | *** |  |
| **e3** | | | | | | | |  | |  | | 0.344 | | | 0.029 | 11.88 | *** |  |
| **e10** | | | | | | | |  | |  | | 0.622 | | | 0.043 | 14.62 | *** |  |
| **e8** | | | | | | | |  | |  | | 0.717 | | | 0.049 | 14.62 | *** |  |

**Modification Indices (Group number 1 - Default model)**

**Covariances: (Group number 1 - Default model)**

|  |  |  | **M.I.** | **Par Change** |  |
| --- | --- | --- | --- | --- | --- |
| e3 | <--> | e10 | 16.911 | 0.087 |  |
| e12 | <--> | e3 | 5.093 | -0.045 |  |
|  |  |  |  |  |  |
| **Variances: (Group number 1 - Default model)** | | | | |  |
|  |  |  |  |  |  |
|  |  |  | **M.I.** | **Par Change** |  |
|  |  |  |  |  |  |
| **Regression Weights: (Group number 1 - Default model)** | | | | | |
|  |  |  |  |  |  |
|  |  |  | **M.I.** | **Par Change** |  |
| Q10 | <--- | Q3 | 5.634 | 0.084 |  |
| Q3 | <--- | Q10 | 9.2 | 0.093 |  |

**Model Fit Summary**

**CMIN**

| **Model** | **NPAR** | **CMIN** | **DF** | **P** | **CMIN/DF** |
| --- | --- | --- | --- | --- | --- |
| **Default model** | 13 | 31.214 | 8 | .000 | 3.902 |
| **Saturated model** | 21 | .000 | 0 |  |  |
| **Independence model** | 6 | 1195.677 | 15 | .000 | 79.712 |

**RMR, GFI**

| **Model** | **RMR** | **GFI** | **AGFI** | **PGFI** |
| --- | --- | --- | --- | --- |
| **Default model** | .023 | .980 | .948 | .373 |
| **Saturated model** | .000 | 1.000 |  |  |
| **Independence model** | .362 | .456 | .238 | .326 |

**Baseline Comparisons**

| **Model** | **NFI Delta1** | **RFI rho1** | **IFI Delta2** | **TLI rho2** | **CFI** |
| --- | --- | --- | --- | --- | --- |
| **Default model** | .974 | .951 | .980 | .963 | .980 |
| **Saturated model** | 1.000 |  | 1.000 |  | 1.000 |
| **Independence model** | .000 | .000 | .000 | .000 | .000 |

**Parsimony-Adjusted Measures**

| **Model** | **PRATIO** | **PNFI** | **PCFI** |
| --- | --- | --- | --- |
| **Default model** | .533 | .519 | .523 |
| **Saturated model** | .000 | .000 | .000 |
| **Independence model** | 1.000 | .000 | .000 |

**NCP**

| **Model** | **NCP** | **LO 90** | **HI 90** |
| --- | --- | --- | --- |
| **Default model** | 23.214 | 9.685 | 44.299 |
| **Saturated model** | .000 | .000 | .000 |
| **Independence model** | 1180.677 | 1070.928 | 1297.803 |

**FMIN**

| **Model** | **FMIN** | **F0** | **LO 90** | **HI 90** |
| --- | --- | --- | --- | --- |
| **Default model** | .061 | .046 | .019 | .087 |
| **Saturated model** | .000 | .000 | .000 | .000 |
| **Independence model** | 2.344 | 2.315 | 2.100 | 2.545 |

**RMSEA**

| **Model** | **RMSEA** | **LO 90** | **HI 90** | **PCLOSE** |
| --- | --- | --- | --- | --- |
| **Default model** | .075 | .049 | .104 | .058 |
| **Independence model** | .393 | .374 | .412 | .000 |

**AIC**

| **Model** | **AIC** | **BCC** | **BIC** | **CAIC** |
| --- | --- | --- | --- | --- |
| **Default model** | 57.214 | 57.576 | 112.287 | 125.287 |
| **Saturated model** | 42.000 | 42.584 | 130.964 | 151.964 |
| **Independence model** | 1207.677 | 1207.844 | 1233.096 | 1239.096 |

**ECVI**

| **Model** | **ECVI** | **LO 90** | **HI 90** | **MECVI** |
| --- | --- | --- | --- | --- |
| **Default model** | .112 | .086 | .154 | .113 |
| **Saturated model** | .082 | .082 | .082 | .083 |
| **Independence model** | 2.368 | 2.153 | 2.598 | 2.368 |

**HOELTER**

| **Model** | **HOELTER .05** | **HOELTER .01** |
| --- | --- | --- |
| **Default model** | 254 | 329 |
| **Independence model** | 11 | 14 |

**Part 5. Confirmatory Factor Analysis of the Model—Instrumental Motivation**


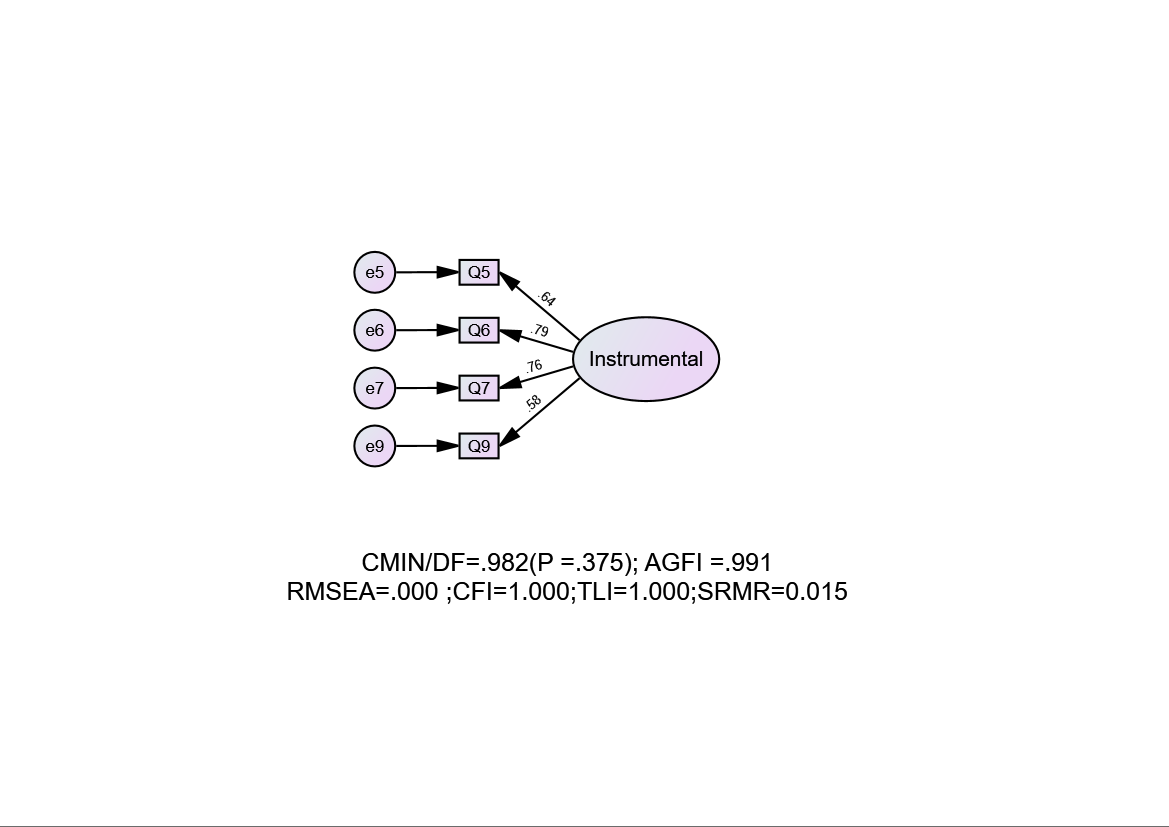


**Estimates (Group number 1 - Default model)**

**Scalar Estimates (Group number 1 - Default model)**

**Maximum Likelihood Estimates**

**Regression Weights: (Group number 1 - Default model)**

|  |  |  | **Estimate** | **S.E.** | **C.R.** | **P** | **Label** |
| --- | --- | --- | --- | --- | --- | --- | --- |
| Q9 | <--- | Instrumental | .896 | .084 | 10.672 | *** |  |
| Q7 | <--- | Instrumental | 1.201 | .093 | 12.851 | *** |  |
| Q6 | <--- | Instrumental | 1.287 | .099 | 12.963 | *** |  |
| Q5 | <--- | Instrumental | 1.000 |  |  |  |  |

**Standardized Regression Weights: (Group number 1 - Default model)**

|  |  |  | **Estimate** |
| --- | --- | --- | --- |
| Q9 | <--- | Instrumental | .581 |
| Q7 | <--- | Instrumental | .764 |
| Q6 | <--- | Instrumental | .788 |
| Q5 | <--- | Instrumental | .643 |

**Variances: (Group number 1 - Default model)**

|  |  |  | **Estimate** | **S.E.** | **C.R.** | **P** | **Label** |
| --- | --- | --- | --- | --- | --- | --- | --- |
| **Instrumental** |  |  | .386 | .053 | 7.314 | *** |  |
| **e9** |  |  | .607 | .043 | 13.991 | *** |  |
| **e7** |  |  | .396 | .039 | 10.157 | *** |  |
| **e6** |  |  | .390 | .042 | 9.279 | *** |  |
| **e5** |  |  | .546 | .041 | 13.191 | *** |  |

**Model Fit Summary**

**CMIN**

| **Model** | **NPAR** | **CMIN** | **DF** | **P** | **CMIN/DF** |
| --- | --- | --- | --- | --- | --- |
| **Default model** | 8 | 1.964 | 2 | .375 | .982 |
| **Saturated model** | 10 | .000 | 0 |  |  |
| **Independence model** | 4 | 580.368 | 6 | .000 | 96.728 |

**RMR, GFI**

| **Model** | **RMR** | **GFI** | **AGFI** | **PGFI** |
| --- | --- | --- | --- | --- |
| **Default model** | .010 | .998 | .991 | .200 |
| **Saturated model** | .000 | 1.000 |  |  |
| **Independence model** | .362 | .586 | .310 | .352 |

**Baseline Comparisons**

| **Model** | **NFI Delta1** | **RFI rho1** | **IFI Delta2** | **TLI rho2** | **CFI** |
| --- | --- | --- | --- | --- | --- |
| **Default model** | .997 | .990 | 1.000 | 1.000 | 1.000 |
| **Saturated model** | 1.000 |  | 1.000 |  | 1.000 |
| **Independence model** | .000 | .000 | .000 | .000 | .000 |

**Parsimony-Adjusted Measures**

| **Model** | **PRATIO** | **PNFI** | **PCFI** |
| --- | --- | --- | --- |
| **Default model** | .333 | .332 | .333 |
| **Saturated model** | .000 | .000 | .000 |
| **Independence model** | 1.000 | .000 | .000 |

**NCP**

| **Model** | **NCP** | **LO 90** | **HI 90** |
| --- | --- | --- | --- |
| **Default model** | .000 | .000 | 7.752 |
| **Saturated model** | .000 | .000 | .000 |
| **Independence model** | 574.368 | 498.980 | 657.156 |

**FMIN**

| **Model** | **FMIN** | **F0** | **LO 90** | **HI 90** |
| --- | --- | --- | --- | --- |
| **Default model** | .004 | .000 | .000 | .015 |
| **Saturated model** | .000 | .000 | .000 | .000 |
| **Independence model** | 1.138 | 1.126 | .978 | 1.289 |

**RMSEA**

| **Model** | **RMSEA** | **LO 90** | **HI 90** | **PCLOSE** |
| --- | --- | --- | --- | --- |
| **Default model** | .000 | .000 | .087 | .714 |
| **Independence model** | .433 | .404 | .463 | .000 |

**AIC**

| **Model** | **AIC** | **BCC** | **BIC** | **CAIC** |
| --- | --- | --- | --- | --- |
| **Default model** | 17.964 | 18.122 | 51.855 | 59.855 |
| **Saturated model** | 20.000 | 20.198 | 62.364 | 72.364 |
| **Independence model** | 588.368 | 588.447 | 605.313 | 609.313 |

**ECVI**

| **Model** | **ECVI** | **LO 90** | **HI 90** | **MECVI** |
| --- | --- | --- | --- | --- |
| **Default model** | .035 | .035 | .050 | .036 |
| **Saturated model** | .039 | .039 | .039 | .040 |
| **Independence model** | 1.154 | 1.006 | 1.316 | 1.154 |

**HOELTER**

| **Model** | **HOELTER .05** | **HOELTER .01** |
| --- | --- | --- |
| **Default model** | 1557 | 2393 |
| **Independence model** | 12 | 15 |

**Part 6. Confirmatory Factor Analysis of English Learning Motivation Model**

**
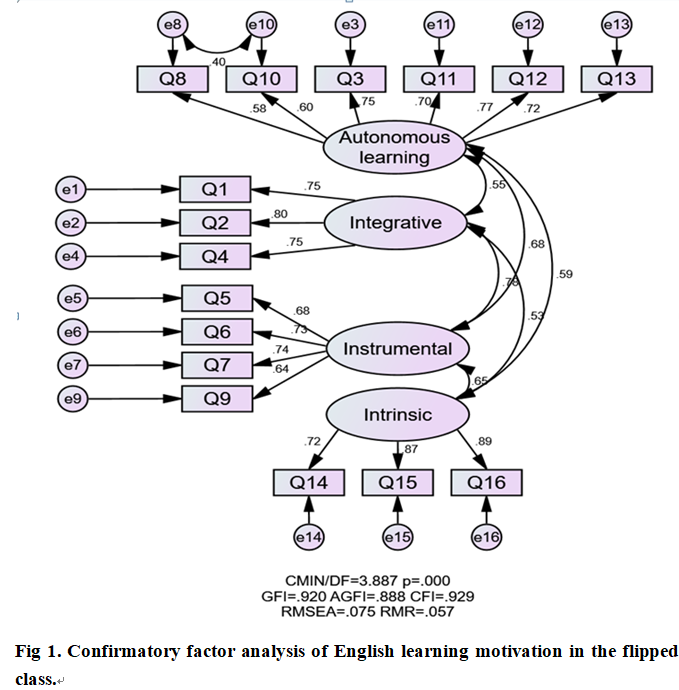
**

**Estimates (Group number 1 - Default model)**

**Scalar Estimates (Group number 1 - Default model)**

**Maximum Likelihood Estimates**

**Regression Weights: (Group number 1 - Default model)**

|  |  |  | Estimate | S.E. | C.R. | P | Label |
| --- | --- | --- | --- | --- | --- | --- | --- |
| Q8 | <--- | Autonomous_learning | 1.000 |  |  |  |  |
| Q10 | <--- | Autonomous_learning | .952 | .069 | 13.792 | *** | par_1 |
| Q3 | <--- | Autonomous_learning | 1.147 | .092 | 12.456 | *** | par_2 |
| Q11 | <--- | Autonomous_learning | 1.063 | .089 | 11.909 | *** | par_3 |
| Q12 | <--- | Autonomous_learning | 1.266 | .100 | 12.609 | *** | par_4 |
| Q13 | <--- | Autonomous_learning | 1.043 | .086 | 12.099 | *** | par_5 |
| Q4 | <--- | Integrative | 1.000 |  |  |  |  |
| Q2 | <--- | Integrative | 1.103 | .068 | 16.334 | *** | par_6 |
| Q1 | <--- | Integrative | 1.030 | .066 | 15.599 | *** | par_7 |
| Q7 | <--- | Instrumental | 1.000 |  |  |  |  |
| Q6 | <--- | Instrumental | 1.020 | .066 | 15.351 | *** | par_8 |
| Q5 | <--- | Instrumental | .901 | .063 | 14.281 | *** | par_9 |
| Q9 | <--- | Instrumental | .840 | .063 | 13.426 | *** | par_10 |
| Q16 | <--- | Intrinsic | 1.000 |  |  |  |  |
| Q15 | <--- | Intrinsic | .995 | .042 | 23.755 | *** | par_12 |
| Q14 | <--- | Intrinsic | .874 | .047 | 18.602 | *** | par_13 |

**Standardized Regression Weights: (Group number 1 - Default model)**

|  |  |  | Estimate |
| --- | --- | --- | --- |
| Q8 | <--- | Autonomous_learning | .583 |
| Q10 | <--- | Autonomous_learning | .596 |
| Q3 | <--- | Autonomous_learning | .754 |
| Q11 | <--- | Autonomous_learning | .700 |
| Q12 | <--- | Autonomous_learning | .770 |
| Q13 | <--- | Autonomous_learning | .718 |
| Q4 | <--- | Integrative | .746 |
| Q2 | <--- | Integrative | .802 |
| Q1 | <--- | Integrative | .754 |
| Q7 | <--- | Instrumental | .745 |
| Q6 | <--- | Instrumental | .731 |
| Q5 | <--- | Instrumental | .679 |
| Q9 | <--- | Instrumental | .638 |
| Q16 | <--- | Intrinsic | .888 |
| Q15 | <--- | Intrinsic | .873 |
| Q14 | <--- | Intrinsic | .721 |

**Covariances: (Group number 1 - Default model)**

|  |  |  | **Estimate** | **S.E.** | **C.R.** | **P** | **Label** |
| --- | --- | --- | --- | --- | --- | --- | --- |
| Autonomous_learning | <--> | Integrative | .226 | .029 | 7.693 | *** | par_14 |
| Autonomous_learning | <--> | Instrumental | .292 | .034 | 8.494 | *** | par_15 |
| Autonomous_learning | <--> | Intrinsic | .286 | .034 | 8.463 | *** | par_16 |
| Integrative | <--> | Instrumental | .388 | .038 | 10.079 | *** | par_17 |
| Integrative | <--> | Intrinsic | .293 | .034 | 8.542 | *** | par_18 |
| Instrumental | <--> | Intrinsic | .381 | .039 | 9.797 | *** | par_19 |
| e8 | <--> | e10 | .251 | .034 | 7.429 | *** | par_11 |

**Correlations: (Group number 1 - Default model)**

|  |  |  | Estimate |
| --- | --- | --- | --- |
| Autonomous_learning | <--> | Integrative | .554 |
| Autonomous_learning | <--> | Instrumental | .675 |
| Autonomous_learning | <--> | Intrinsic | .592 |
| Integrative | <--> | Instrumental | .780 |
| Integrative | <--> | Intrinsic | .526 |
| Instrumental | <--> | Intrinsic | .645 |
| e8 | <--> | e10 | .395 |

**Variances: (Group number 1 - Default model)**

|  |  |  | Estimate | S.E. | C.R. | P | Label |
| --- | --- | --- | --- | --- | --- | --- | --- |
| Autonomous_learning |  |  | .354 | .053 | 6.738 | *** | par_20 |
| Integrative |  |  | .469 | .051 | 9.122 | *** | par_21 |
| Instrumental |  |  | .528 | .058 | 9.174 | *** | par_22 |
| Intrinsic |  |  | .660 | .054 | 12.116 | *** | par_23 |
| e8 |  |  | .688 | .047 | 14.615 | *** | par_24 |
| e10 |  |  | .584 | .040 | 14.531 | *** | par_25 |
| e3 |  |  | .355 | .028 | 12.607 | *** | par_26 |
| e11 |  |  | .416 | .031 | 13.516 | *** | par_27 |
| e12 |  |  | .390 | .032 | 12.245 | *** | par_28 |
| e13 |  |  | .362 | .027 | 13.251 | *** | par_29 |
| e4 |  |  | .373 | .031 | 12.106 | *** | par_30 |
| e2 |  |  | .316 | .030 | 10.398 | *** | par_31 |
| e1 |  |  | .377 | .032 | 11.895 | *** | par_32 |
| e7 |  |  | .425 | .034 | 12.418 | *** | par_33 |
| e6 |  |  | .479 | .038 | 12.704 | *** | par_34 |
| e5 |  |  | .503 | .037 | 13.562 | *** | par_35 |
| e9 |  |  | .544 | .039 | 14.046 | *** | par_36 |
| e16 |  |  | .177 | .022 | 8.208 | *** | par_37 |
| e15 |  |  | .204 | .022 | 9.091 | *** | par_38 |
| e14 |  |  | .466 | .034 | 13.902 | *** | par_39 |

**Modification Indices (Group number 1 - Default model)**

**Covariances: (Group number 1 - Default model)**

|  |  |  | M.I. | Par Change |
| --- | --- | --- | --- | --- |
| e14 | <--> | Intrinsic | 10.785 | -.071 |
| e14 | <--> | Integrative | 8.579 | .054 |
| e14 | <--> | Autonomous_learning | 4.617 | .035 |
| e15 | <--> | Autonomous_learning | 9.020 | -.037 |
| e9 | <--> | Instrumental | 8.476 | -.054 |
| e9 | <--> | Autonomous_learning | 39.825 | .110 |
| e9 | <--> | e16 | 5.003 | .041 |
| e5 | <--> | Integrative | 9.556 | .059 |
| e6 | <--> | Instrumental | 6.666 | .046 |
| e6 | <--> | Autonomous_learning | 5.961 | -.041 |
| e7 | <--> | e6 | 14.310 | .090 |
| e1 | <--> | Intrinsic | 4.983 | -.046 |
| e1 | <--> | Instrumental | 4.076 | .034 |
| e1 | <--> | e5 | 4.331 | .048 |
| e2 | <--> | e14 | 4.672 | .046 |
| e4 | <--> | Autonomous_learning | 4.397 | .032 |
| e4 | <--> | e1 | 4.217 | -.041 |
| e12 | <--> | Instrumental | 4.866 | -.038 |
| e12 | <--> | Integrative | 9.009 | .053 |
| e12 | <--> | e15 | 5.377 | -.039 |
| e12 | <--> | e16 | 5.260 | .038 |
| e12 | <--> | e5 | 4.327 | .049 |
| e12 | <--> | e6 | 10.239 | -.075 |
| e12 | <--> | e7 | 5.972 | -.054 |
| e12 | <--> | e4 | 8.965 | .063 |
| e12 | <--> | e13 | 4.404 | .041 |
| e3 | <--> | Integrative | 4.054 | -.034 |
| e3 | <--> | e5 | 13.340 | -.081 |
| e3 | <--> | e6 | 6.674 | .057 |
| e3 | <--> | e1 | 5.217 | -.046 |
| e10 | <--> | Intrinsic | 13.881 | .081 |
| e10 | <--> | Autonomous_learning | 9.714 | -.050 |
| e10 | <--> | e15 | 6.839 | .046 |
| e10 | <--> | e9 | 7.083 | .065 |
| e10 | <--> | e12 | 9.075 | -.066 |
| e10 | <--> | e11 | 7.413 | -.059 |
| e10 | <--> | e3 | 9.905 | .065 |
| e8 | <--> | Intrinsic | 7.248 | -.063 |
| e8 | <--> | Instrumental | 20.357 | .087 |
| e8 | <--> | e9 | 38.526 | .165 |

**Variances: (Group number 1 - Default model)**

|  |  |  | M.I. | Par Change |
| --- | --- | --- | --- | --- |

**Regression Weights: (Group number 1 - Default model)**

|  |  |  | M.I. | Par Change |
| --- | --- | --- | --- | --- |
| Q14 | <--- | Instrumental | 9.440 | .147 |
| Q14 | <--- | Integrative | 14.942 | .197 |
| Q14 | <--- | Autonomous_learning | 8.848 | .172 |
| Q14 | <--- | Q5 | 7.294 | .090 |
| Q14 | <--- | Q6 | 7.466 | .086 |
| Q14 | <--- | Q7 | 4.466 | .069 |
| Q14 | <--- | Q1 | 10.847 | .113 |
| Q14 | <--- | Q2 | 15.567 | .134 |
| Q14 | <--- | Q4 | 6.826 | .091 |
| Q14 | <--- | Q13 | 4.959 | .083 |
| Q14 | <--- | Q12 | 4.021 | .066 |
| Q14 | <--- | Q11 | 9.431 | .109 |
| Q14 | <--- | Q3 | 6.138 | .088 |
| Q14 | <--- | Q8 | 5.545 | .074 |
| Q15 | <--- | Autonomous_learning | 5.676 | -.105 |
| Q15 | <--- | Q12 | 9.196 | -.076 |
| Q15 | <--- | Q11 | 7.561 | -.074 |
| Q15 | <--- | Q3 | 4.816 | -.059 |
| Q9 | <--- | Autonomous_learning | 19.567 | .275 |
| Q9 | <--- | Q16 | 5.026 | .084 |
| Q9 | <--- | Q13 | 4.773 | .087 |
| Q9 | <--- | Q12 | 14.831 | .135 |
| Q9 | <--- | Q11 | 4.762 | .083 |
| Q9 | <--- | Q3 | 14.964 | .147 |
| Q9 | <--- | Q10 | 46.948 | .248 |
| Q9 | <--- | Q8 | 76.089 | .294 |
| Q5 | <--- | Q1 | 5.238 | .082 |
| Q5 | <--- | Q3 | 7.069 | -.098 |
| Q6 | <--- | Q7 | 5.449 | .080 |
| Q6 | <--- | Q12 | 9.277 | -.105 |
| Q7 | <--- | Q6 | 5.794 | .076 |
| Q7 | <--- | Q12 | 5.138 | -.074 |
| Q2 | <--- | Q8 | 5.142 | -.065 |
| Q4 | <--- | Intrinsic | 4.711 | .085 |
| Q4 | <--- | Autonomous_learning | 5.204 | .124 |
| Q4 | <--- | Q15 | 5.928 | .079 |
| Q4 | <--- | Q12 | 11.194 | .103 |
| Q4 | <--- | Q10 | 6.282 | .079 |
| Q12 | <--- | Q6 | 4.394 | -.064 |
| Q12 | <--- | Q4 | 7.543 | .092 |
| Q12 | <--- | Q10 | 5.990 | -.079 |
| Q11 | <--- | Q10 | 5.930 | -.078 |
| Q3 | <--- | Q5 | 10.116 | -.096 |
| Q3 | <--- | Q1 | 6.755 | -.081 |
| Q3 | <--- | Q10 | 4.325 | .064 |
| Q10 | <--- | Intrinsic | 11.827 | .141 |
| Q10 | <--- | Instrumental | 4.383 | .099 |
| Q10 | <--- | Q15 | 15.116 | .133 |
| Q10 | <--- | Q16 | 10.707 | .113 |
| Q10 | <--- | Q9 | 10.098 | .105 |
| Q10 | <--- | Q7 | 5.241 | .074 |
| Q10 | <--- | Q4 | 4.871 | .076 |
| Q8 | <--- | Q9 | 30.657 | .198 |

**Model Fit Summary**

**CMIN**

| Model | NPAR | CMIN | DF | P | CMIN/DF |
| --- | --- | --- | --- | --- | --- |
| Default model | 39 | 377.042 | 97 | .000 | 3.887 |
| Saturated model | 136 | .000 | 0 |  |  |
| Independence model | 16 | 4091.288 | 120 | .000 | 34.094 |

**RMR, GFI**

| Model | RMR | GFI | AGFI | PGFI |
| --- | --- | --- | --- | --- |
| Default model | .057 | .920 | .888 | .656 |
| Saturated model | .000 | 1.000 |  |  |
| Independence model | .342 | .289 | .194 | .255 |

**Baseline Comparisons**

| Model | NFI Delta1 | RFI rho1 | IFI Delta2 | TLI rho2 | CFI |
| --- | --- | --- | --- | --- | --- |
| Default model | .908 | .886 | .930 | .913 | .929 |
| Saturated model | 1.000 |  | 1.000 |  | 1.000 |
| Independence model | .000 | .000 | .000 | .000 | .000 |

**Parsimony-Adjusted Measures**

| Model | PRATIO | PNFI | PCFI |
| --- | --- | --- | --- |
| Default model | .808 | .734 | .751 |
| Saturated model | .000 | .000 | .000 |
| Independence model | 1.000 | .000 | .000 |

**NCP**

| Model | NCP | LO 90 | HI 90 |
| --- | --- | --- | --- |
| Default model | 280.042 | 224.155 | 343.500 |
| Saturated model | .000 | .000 | .000 |
| Independence model | 3971.288 | 3765.968 | 4183.876 |

**FMIN**

| Model | FMIN | F0 | LO 90 | HI 90 |
| --- | --- | --- | --- | --- |
| Default model | .739 | .549 | .440 | .674 |
| Saturated model | .000 | .000 | .000 | .000 |
| Independence model | 8.022 | 7.787 | 7.384 | 8.204 |

**RMSEA**

| Model | RMSEA | LO 90 | HI 90 | PCLOSE |
| --- | --- | --- | --- | --- |
| Default model | .075 | .067 | .083 | .000 |
| Independence model | .255 | .248 | .261 | .000 |

**AIC**

| Model | AIC | BCC | BIC | CAIC |
| --- | --- | --- | --- | --- |
| Default model | 455.042 | 457.732 | 620.261 | 659.261 |
| Saturated model | 272.000 | 281.379 | 848.146 | 984.146 |
| Independence model | 4123.288 | 4124.391 | 4191.069 | 4207.069 |

**ECVI**

| Model | ECVI | LO 90 | HI 90 | MECVI |
| --- | --- | --- | --- | --- |
| Default model | .892 | .783 | 1.017 | .898 |
| Saturated model | .533 | .533 | .533 | .552 |
| Independence model | 8.085 | 7.682 | 8.502 | 8.087 |

**HOELTER**

| Model | HOELTER .05 | HOELTER .01 |
| --- | --- | --- |
| Default model | 164 | 179 |
| Independence model | 19 | 20 |

**Part 7. data sets for research question1**

**1, Differences in pre-test scores of English learning motivation between the experimental class and the control class (1=experimental; 2=control)**

| **Group Statistics** | | | | | |
| --- | --- | --- | --- | --- | --- |
| group | | N | Mean | Std. Deviation | Std. Error Mean |
| Integrative_pretest | 1 | 35 | 2.86 | 0.71 | .12014 |
|  | 2 | 35 | 2.92 | 0.73 | .12381 |
| Instrumental_pretest | 1 | 35 | 2.63 | 0.99 | .16759 |
|  | 2 | 35 | 2.63 | 0.70 | .11836 |
| autonomouslearning_pretest | 1 | 35 | 2.83 | 1.12 | .18929 |
|  | 2 | 35 | 2.72 | 0.84 | .14280 |
| Intrinsic_pretest | 1 | 35 | 2.75 | 0.88 | .14866 |
|  | 2 | 35 | 2.93 | 0.99 | .16725 |
| Totalmotivation_pretest | 1 | 35 | 2.77 | 0.68 | .11468 |
|  | 2 | 35 | 2.80 | 0.58 | .09745 |

| **Independent Samples Test** | | | | | | | | | | | | | | | | | | | |
| --- | --- | --- | --- | --- | --- | --- | --- | --- | --- | --- | --- | --- | --- | --- | --- | --- | --- | --- | --- |
|  | | Levene's Test for Equality of Variances | | | t-test for Equality of Means | | | | | | | | | | | | | | |
|  |  | F | Sig. | | t | | df | | | Sig. (2-tailed) | | Mean Difference | | Std. Error Difference | | 95% Confidence Interval of the Difference | | | |
|  |  |  |  |  |  |  |  |  |  |  |  |  |  |  |  | Lower | | Upper | |
| Integrative_pretest | Equal variances assumed | | | .004 | | .949 | | -.386 | 68 | | .700 | | -.06667 | | .17251 | | -.41091 | | .27758 |
|  | Equal variances not assumed | | |  | |  | | -.386 | 67.938 | | .700 | | -.06667 | | .17251 | | -.41092 | | .27759 |
| Instrumental_pretest | Equal variances assumed | | | 4.999 | | .029 | | 0.000 | 68 | | 1.000 | | 0.00000 | | .20517 | | -.40941 | | .40941 |
|  | Equal variances not assumed | | |  | |  | | 0.000 | 61.162 | | 1.000 | | 0.00000 | | .20517 | | -.41024 | | .41024 |
| autonomouslearning_pretest | Equal variances assumed | | | 5.242 | | .025 | | .462 | 68 | | .646 | | .10952 | | .23711 | | -.36362 | | .58267 |
|  | Equal variances not assumed | | |  | |  | | .462 | 63.231 | | .646 | | .10952 | | .23711 | | -.36427 | | .58332 |
| Intrinsic_pretest | Equal variances assumed | | | .492 | | .486 | | -.809 | 68 | | .422 | | -.18095 | | .22377 | | -.62748 | | .26558 |
|  | Equal variances not assumed | | |  | |  | | -.809 | 67.077 | | .422 | | -.18095 | | .22377 | | -.62759 | | .26569 |
| Totalmotivation_pretest | Equal variances assumed | | | .730 | | .396 | | -.229 | 68 | | .819 | | -.03452 | | .15049 | | -.33482 | | .26578 |
|  | Equal variances not assumed | | |  | |  | | -.229 | 66.274 | | .819 | | -.03452 | | .15049 | | -.33497 | | .26592 |

| **Independent Samples Effect Sizes** | | | | | |
| --- | --- | --- | --- | --- | --- |
|  | | Standardizer | Point Estimate | 95% Confidence Interval | |
|  |  |  |  | Lower | Upper |
| Integrative_pretest | Cohen's d | 0.72168 | -0.092 | -0.561 | 0.377 |
|  | Hedges' correction | 0.72976 | -0.091 | -0.555 | 0.373 |
|  | Glass's delta | 0.73247 | -0.091 | -0.559 | 0.379 |
| Instrumental_pretest | Cohen's d | 0.85829 | 0.000 | -0.469 | 0.469 |
|  | Hedges' correction | 0.86790 | 0.000 | -0.463 | 0.463 |
|  | Glass's delta | 0.70024 | 0.000 | -0.469 | 0.469 |
| autonomouslearning_pretest | Cohen's d | 0.99190 | 0.110 | -0.359 | 0.579 |
|  | Hedges' correction | 1.00301 | 0.109 | -0.355 | 0.572 |
|  | Glass's delta | 0.84480 | 0.130 | -0.341 | 0.598 |
| Intrinsic_pretest | Cohen's d | 0.93610 | -0.193 | -0.662 | 0.277 |
|  | Hedges' correction | 0.94659 | -0.191 | -0.655 | 0.274 |
|  | Glass's delta | 0.98949 | -0.183 | -0.652 | 0.289 |
| Totalmotivation_pretest | Cohen's d | 0.62955 | -0.055 | -0.523 | 0.414 |
|  | Hedges' correction | 0.63660 | -0.054 | -0.517 | 0.409 |
|  | Glass's delta | 0.57652 | -0.060 | -0.528 | 0.409 |
|  | | | | | |

**2, Differences in post-test scores of English learning motivation between the experimental class and the control class**

| **Group Statistics** | | | | | |
| --- | --- | --- | --- | --- | --- |
| group | | N | Mean | Std. Deviation | Std. Error Mean |
| Integrative_posttest | 1 | 35 | 3.53 | 0.90 | .15291 |
|  | 2 | 35 | 2.97 | 0.87 | .14678 |
| Instrumental_posttest | 1 | 35 | 3.34 | 0.91 | .15411 |
|  | 2 | 35 | 2.72 | 0.89 | .15090 |
| autonomouslearning_posttest | 1 | 35 | 3.84 | 0.78 | .13230 |
|  | 2 | 35 | 2.80 | 0.79 | .13373 |
| Intrinsic_posttest | 1 | 35 | 3.58 | 0.94 | .15956 |
|  | 2 | 35 | 2.95 | 0.88 | .14790 |
| Totalmotivation_posttest | 1 | 35 | 3.58 | 0.69 | .11693 |
|  | 2 | 35 | 2.86 | 0.64 | .10873 |

| **Independent Samples Test** | | | | | | | | | | |
| --- | --- | --- | --- | --- | --- | --- | --- | --- | --- | --- |
|  | | Levene's Test for Equality of Variances | | t-test for Equality of Means | | | | | | |
|  |  | F | Sig. | t | df | Sig. (2-tailed) | Mean Difference | Std. Error Difference | 95% Confidence Interval of the Difference | |
|  |  |  |  |  |  |  |  |  | Lower | Upper |
| Integrative_posttest | Equal variances assumed | .077 | .783 | 2.651 | 68 | .010 | .56190 | .21195 | .13896 | .98485 |
|  | Equal variances not assumed |  |  | 2.651 | 67.887 | .010 | .56190 | .21195 | .13894 | .98487 |
| Instrumental_posttest | Equal variances assumed | .012 | .913 | 2.881 | 68 | .005 | .62143 | .21568 | .19104 | 1.05181 |
|  | Equal variances not assumed |  |  | 2.881 | 67.970 | .005 | .62143 | .21568 | .19104 | 1.05182 |
| autonomouslearning_posttest | Equal variances assumed | .022 | .882 | 5.569 | 68 | .000 | 1.04762 | .18812 | .67223 | 1.42301 |
|  | Equal variances not assumed |  |  | 5.569 | 67.992 | .000 | 1.04762 | .18812 | .67223 | 1.42301 |
| Intrinsic_posttest | Equal variances assumed | .886 | .350 | 2.889 | 68 | .005 | .62857 | .21757 | .19442 | 1.06272 |
|  | Equal variances not assumed |  |  | 2.889 | 67.612 | .005 | .62857 | .21757 | .19437 | 1.06277 |
| Totalmotivation_posttest | Equal variances assumed | .356 | .553 | 4.477 | 68 | .000 | .71488 | .15967 | .39627 | 1.03350 |
|  | Equal variances not assumed |  |  | 4.477 | 67.644 | .000 | .71488 | .15967 | .39623 | 1.03353 |

| **Independent Samples Effect Sizes** | | | | | |
| --- | --- | --- | --- | --- | --- |
|  | | Standardizer | Point Estimate | 95% Confidence Interval | |
|  |  |  |  | Lower | Upper |
| Integrative_posttest | Cohen's d | 0.88667 | 0.634 | 0.151 | 1.112 |
|  | Hedges' correction | 0.89660 | 0.627 | 0.149 | 1.100 |
|  | Glass's delta | 0.86837 | 0.647 | 0.150 | 1.136 |
| Instrumental_posttest | Cohen's d | 0.90226 | 0.689 | 0.204 | 1.169 |
|  | Hedges' correction | 0.91237 | 0.681 | 0.202 | 1.156 |
|  | Glass's delta | 0.89272 | 0.696 | 0.195 | 1.188 |
| autonomouslearning_posttest | Cohen's d | 0.78696 | 1.331 | 0.808 | 1.846 |
|  | Hedges' correction | 0.79577 | 1.316 | 0.799 | 1.826 |
|  | Glass's delta | 0.79117 | 1.324 | 0.754 | 1.881 |
| Intrinsic_posttest | Cohen's d | 0.91016 | 0.691 | 0.206 | 1.171 |
|  | Hedges' correction | 0.92035 | 0.683 | 0.203 | 1.158 |
|  | Glass's delta | 0.87501 | 0.718 | 0.215 | 1.212 |
| Totalmotivation_posttest | Cohen's d | 0.66795 | 1.070 | 0.565 | 1.569 |
|  | Hedges' correction | 0.67543 | 1.058 | 0.559 | 1.551 |
|  | Glass's delta | 0.64327 | 1.111 | 0.568 | 1.642 |

**3, Differences in English learning motivation between the pre-test and the post-test for the control class**

| **Paired Samples Statistics** | | | | | |
| --- | --- | --- | --- | --- | --- |
|  | | Mean | N | Std. Deviation | Std. Error Mean |
| Pair 1 | integrative_pretest | 2.92 | 35 | 0.73 | .12381 |
|  | integrative_posttest | 2.97 | 35 | 0.87 | .14678 |
| Pair 2 | Instrumental_pretest | 2.63 | 35 | 0.70 | .11836 |
|  | Instrumental_posttest | 2.72 | 35 | 0.89 | .15090 |
| Pair 3 | autonomouslearning_pretest | 2.72 | 35 | 0.84 | .14280 |
|  | autonomouslearning_posttest | 2.80 | 35 | 0.79 | .13373 |
| Pair 4 | Intrinsic_pretest | 2.93 | 35 | 0.99 | .16725 |
|  | Intrinsic_posttest | 2.95 | 35 | 0.88 | .14790 |
| Pair 5 | Totalmotivation_pretest | 2.80 | 35 | 0.58 | .09745 |
|  | Totalmotivation_posttest | 2.86 | 35 | 0.64 | .10873 |

| **Paired Samples Test** | | | | | | | | | |
| --- | --- | --- | --- | --- | --- | --- | --- | --- | --- |
|  | | Paired Differences | | | | | t | df | Sig. (2-tailed) |
|  |  | Mean | Std. Deviation | Std. Error Mean | 95% Confidence Interval of the Difference | |  |  |  |
|  |  |  |  |  | Lower | Upper |  |  |  |
| Pair 1 | integrative_pretest - integrative_posttest | -.04762 | .97062 | .16407 | -.38104 | .28580 | -.290 | 34 | .773 |
| Pair 2 | Instrumental_pretest - Instrumental_posttest | -.09286 | .85982 | .14534 | -.38821 | .20250 | -.639 | 34 | .527 |
| Pair 3 | autonomouslearning_pretest - autonomouslearning_posttest | -.07619 | .69233 | .11702 | -.31401 | .16163 | -.651 | 34 | .519 |
| Pair 4 | Intrinsic_pretest - Intrinsic_posttest | -.01905 | 1.12878 | .19080 | -.40680 | .36870 | -.100 | 34 | .921 |
| Pair 5 | Totalmotivation_pretest - Totalmotivation_posttest | -.05893 | .37170 | .06283 | -.18661 | .06875 | -.938 | 34 | .355 |

| **Paired Samples Effect Sizes** | | | | | | |
| --- | --- | --- | --- | --- | --- | --- |
|  | | | Standardizer | Point Estimate | 95% Confidence Interval | |
|  |  |  |  |  | Lower | Upper |
| Pair 1 | integrative_pretest - integrative_posttest | Cohen's d | 0.97062 | -0.049 | -0.380 | 0.283 |
|  |  | Hedges' correction | 0.98150 | -0.049 | -0.376 | 0.280 |
| Pair 2 | Instrumental_pretest - Instrumental_posttest | Cohen's d | 0.85982 | -0.108 | -0.439 | 0.225 |
|  |  | Hedges' correction | 0.86945 | -0.107 | -0.435 | 0.223 |
| Pair 3 | autonomouslearning_pretest - autonomouslearning_posttest | Cohen's d | 0.69233 | -0.110 | -0.442 | 0.223 |
|  |  | Hedges' correction | 0.70008 | -0.109 | -0.437 | 0.221 |
| Pair 4 | Intrinsic_pretest - Intrinsic_posttest | Cohen's d | 1.12878 | -0.017 | -0.348 | 0.315 |
|  |  | Hedges' correction | 1.14142 | -0.017 | -0.344 | 0.311 |
| Pair 5 | Totalmotivation_pretest - Totalmotivation_posttest | Cohen's d | 0.37170 | -0.159 | -0.491 | 0.176 |
|  |  | Hedges' correction | 0.37586 | -0.157 | -0.485 | 0.174 |

**4, Differences in English learning motivation between the pre-test and the post-test for the experimental class**

| **Paired Samples Statistics** | | | | | |
| --- | --- | --- | --- | --- | --- |
|  | | Mean | N | Std. Deviation | Std. Error Mean |
| Pair 1 | Integrative_pretest | 2.86 | 35 | 0.71 | .12014 |
|  | Integrative_posttest | 3.53 | 35 | 0.90 | .15291 |
| Pair 2 | Instrumental_pretest | 2.63 | 35 | 0.99 | .16759 |
|  | Instrumental_posttest | 3.34 | 35 | 0.91 | .15411 |
| Pair 3 | autonomouslearning_pretest | 2.83 | 35 | 1.12 | .18929 |
|  | autonomouslearning_posttest | 3.84 | 35 | 0.78 | .13230 |
| Pair 4 | Intrinsic_pretest | 2.75 | 35 | 0.88 | .14866 |
|  | Intrinsic_posttest | 3.58 | 35 | 0.94 | .15956 |
| Pair 5 | Totalmotivation_pretest | 2.77 | 35 | 0.68 | .11468 |
|  | Totalmotivation_posttest | 3.58 | 35 | 0.69 | .11693 |

| **Paired Samples Test** | | | | | | | | | |
| --- | --- | --- | --- | --- | --- | --- | --- | --- | --- |
|  | | Paired Differences | | | | | t | df | Sig. (2-tailed) |
|  |  | Mean | Std. Deviation | Std. Error Mean | 95% Confidence Interval of the Difference | |  |  |  |
|  |  |  |  |  | Lower | Upper |  |  |  |
| Pair 1 | Integrative_pretest - Integrative_posttest | -.67619 | .89108 | .15062 | -.98229 | -.37009 | -4.489 | 34 | .000 |
| Pair 2 | Instrumental_pretest - Instrumental_posttest | -.71429 | .99103 | .16751 | -1.05472 | -.37385 | -4.264 | 34 | .000 |
| Pair 3 | autonomouslearning_pretest - autonomouslearning_posttest | -1.01429 | 1.08116 | .18275 | -1.38568 | -.64289 | -5.550 | 34 | .000 |
| Pair 4 | Intrinsic_pretest - Intrinsic_posttest | -.82857 | 1.06449 | .17993 | -1.19424 | -.46291 | -4.605 | 34 | .000 |
| Pair 5 | Totalmotivation_pretest - Totalmotivation_posttest | -.80833 | .64555 | .10912 | -1.03009 | -.58658 | -7.408 | 34 | .000 |

| **Paired Samples Effect Sizes** | | | | | | |
| --- | --- | --- | --- | --- | --- | --- |
|  | | | Standardizer | Point Estimate | 95% Confidence Interval | |
|  |  |  |  |  | Lower | Upper |
| Pair 1 | Integrative_pretest - Integrative_posttest | Cohen's d | 0.8911 | -0.759 | -1.131 | -0.378 |
|  |  | Hedges' correction | 0.9011 | -0.750 | -1.119 | -0.373 |
| Pair 2 | Instrumental_pretest - Instrumental_posttest | Cohen's d | 0.9910 | -0.721 | -1.089 | -0.344 |
|  |  | Hedges' correction | 1.0021 | -0.713 | -1.077 | -0.340 |
| Pair 3 | autonomouslearning_pretest - autonomouslearning_posttest | Cohen's d | 1.0812 | -0.938 | -1.332 | -0.534 |
|  |  | Hedges' correction | 1.0933 | -0.928 | -1.317 | -0.529 |
| Pair 4 | Intrinsic_pretest - Intrinsic_posttest | Cohen's d | 1.0645 | -0.778 | -1.153 | -0.395 |
|  |  | Hedges' correction | 1.0764 | -0.770 | -1.140 | -0.390 |
| Pair 5 | Totalmotivation_pretest - Totalmotivation_posttest | Cohen's d | 0.6456 | -1.252 | -1.691 | -0.802 |
|  |  | Hedges' correction | 0.6528 | -1.238 | -1.673 | -0.793 |

**Part 8. data sets for research question2**

**1, Differences in pre-test scores of English proficiency between the experimental and the control class**

| **Group Statistics** | | | | | | | | | | |  |  |  |  |  |  |
| --- | --- | --- | --- | --- | --- | --- | --- | --- | --- | --- | --- | --- | --- | --- | --- | --- |
| group | | | | N | Mean | | Std. Deviation | | | Std. Error Mean |  |  |  |  |  |  |
| Englishproficiency_pretest | | 1 | | 35 | 61.20 | | 7.843 | | | 1.326 |  |  |  |  |  |  |
|  |  | 2 | | 35 | 62.23 | | 9.852 | | | 1.665 |  |  |  |  |  |  |
| **Independent Samples Test** | | | | | | | | | | | | | | | | |
|  | | | Levene's Test for Equality of Variances | | | | | t-test for Equality of Means | | | | | | | | |
|  |  |  | F | | | Sig. | | t | df | | | Sig. (2-tailed) | Mean Difference | Std. Error Difference | 95% Confidence Interval of the Difference | |
|  |  |  |  |  |  |  |  |  |  |  |  |  |  |  | Lower | Upper |
| Englishproficiency_pretest | Equal variances assumed | | 2.312 | | | .133 | | -.483 | 68 | | | .630 | -1.029 | 2.129 | -5.276 | 3.219 |
|  | Equal variances not assumed | |  | | |  | | -.483 | 64.747 | | | .631 | -1.029 | 2.129 | -5.280 | 3.223 |

| **Independent Samples Effect Sizes** | | | | | |
| --- | --- | --- | --- | --- | --- |
|  | | Standardizer | Point Estimate | 95% Confidence Interval | |
|  |  |  |  | Lower | Upper |
| Englishproficiency_pretest | Cohen's d | 8.905 | -0.116 | -0.584 | 0.354 |
|  | Hedges' correction | 9.004 | -0.114 | -0.578 | 0.350 |
|  | Glass's delta | 9.852 | -0.104 | -0.573 | 0.366 |

**2, Differences in post-test scores of English proficiency between the experimental and the control class**

| **Group Statistics** | | | | | |
| --- | --- | --- | --- | --- | --- |
| group | | N | Mean | Std. Deviation | Std. Error Mean |
| Englishproficiency_posttest | 1 | 35 | 68.06 | 7.996 | 1.352 |
|  | 2 | 35 | 61.71 | 7.913 | 1.338 |

| **Independent Samples Test** | | | | | | | | | | |
| --- | --- | --- | --- | --- | --- | --- | --- | --- | --- | --- |
|  | | Levene's Test for Equality of Variances | | t-test for Equality of Means | | | | | | |
|  |  | F | Sig. | t | df | Sig. (2-tailed) | Mean Difference | Std. Error Difference | 95% Confidence Interval of the Difference | |
|  |  |  |  |  |  |  |  |  | Lower | Upper |
| Englishproficiency_posttest | Equal variances assumed | .000 | .997 | 3.336 | 68 | .001 | 6.343 | 1.902 | 2.548 | 10.137 |
|  | Equal variances not assumed |  |  | 3.336 | 67.993 | .001 | 6.343 | 1.902 | 2.548 | 10.137 |

| **Independent Samples Effect Sizes** | | | | | |
| --- | --- | --- | --- | --- | --- |
|  | | Standardizer | Point Estimate | 95% Confidence Interval | |
|  |  |  |  | Lower | Upper |
| Englishproficiency_posttest | Cohen's d | 7.955 | 0.797 | 0.307 | 1.282 |
|  | Hedges' correction | 8.044 | 0.789 | 0.304 | 1.268 |
|  | Glass's delta | 7.913 | 0.802 | 0.291 | 1.302 |

**3, Differences in the scores of English proficiency between the pre-test and the post-test for the control class**

| **Paired Samples Statistics** | | | | | |
| --- | --- | --- | --- | --- | --- |
|  | | Mean | N | Std. Deviation | Std. Error Mean |
| Pair 1 | Englishproficiency_pretest | 62.23 | 35 | 9.852 | 1.665 |
|  | Englishproficiency_posttest | 61.71 | 35 | 7.913 | 1.338 |

| **Paired Samples Test** | | | | | | | | | |
| --- | --- | --- | --- | --- | --- | --- | --- | --- | --- |
|  | | Paired Differences | | | | | t | df | Sig. (2-tailed) |
|  |  | Mean | Std. Deviation | Std. Error Mean | 95% Confidence Interval of the Difference | |  |  |  |
|  |  |  |  |  | Lower | Upper |  |  |  |
| Pair 1 | Englishproficiency_pretest - Englishproficiency_posttest | .514 | 9.189 | 1.553 | -2.642 | 3.671 | .331 | 34 | .743 |

| **Paired Samples Effect Sizes** | | | | | | |
| --- | --- | --- | --- | --- | --- | --- |
|  | | | Standardizer | Point Estimate | 95% Confidence Interval | |
|  |  |  |  |  | Lower | Upper |
| Pair 1 | Englishproficiency_pretest - Englishproficiency_posttest | Cohen's d | 9.189 | 0.056 | -0.276 | 0.387 |
|  |  | Hedges' correction | 9.292 | 0.055 | -0.273 | 0.383 |

**4, Differences in the scores of English proficiency between the pre-test and the post-test for the experimental class**

| **Paired Samples Statistics** | | | | | |
| --- | --- | --- | --- | --- | --- |
|  | | Mean | N | Std. Deviation | Std. Error Mean |
| Pair 1 | Englishproficiency_pretest | 61.20 | 35 | 7.843 | 1.326 |
|  | Englishproficiency_posttest | 68.06 | 35 | 7.996 | 1.352 |

| **Paired Samples Test** | | | | | | | | | |
| --- | --- | --- | --- | --- | --- | --- | --- | --- | --- |
|  | | Paired Differences | | | | | t | df | Sig. (2-tailed) |
|  |  | Mean | Std. Deviation | Std. Error Mean | 95% Confidence Interval of the Difference | |  |  |  |
|  |  |  |  |  | Lower | Upper |  |  |  |
| Pair 1 | Englishproficiency_pretest - Englishproficiency_posttest | -6.857 | 9.696 | 1.639 | -10.188 | -3.527 | -4.184 | 34 | .000 |

| **Paired Samples Effect Sizes** | | | | | | |
| --- | --- | --- | --- | --- | --- | --- |
|  | | | Standardizer | Point Estimate | 95% Confidence Interval | |
|  |  |  |  |  | Lower | Upper |
| Pair 1 | Englishproficiency_pretest - Englishproficiency_posttest | Cohen's d | 9.696 | -0.707 | -1.074 | -0.332 |
|  |  | Hedges' correction | 9.804 | -0.699 | -1.062 | -0.328 |

**Part 9. data sets for research question3**

**Predictability of motivational factors in English proficiency (RQ3)**

| **Descriptive Statistics** | | | |
| --- | --- | --- | --- |
|  | Mean | Std. Deviation | N |
| Englishproficiency_posttest | 64.89 | 8.519 | 70 |
| Integrative_posttest | 3.2524 | .92459 | 70 |
| Instrumental_posttest | 3.0321 | .94880 | 70 |
| autonomouslearning_posttest | 3.3190 | .94270 | 70 |
| Intrinsic_posttest | 3.2667 | .95739 | 70 |

| **Correlations** | | | | | | |
| --- | --- | --- | --- | --- | --- | --- |
|  | | Englishproficiency_posttest | Integrative_posttest | Instrumental_posttest | autonomouslearning_posttest | Intrinsic_posttest |
| Pearson Correlation | Englishproficiency_posttest | 1.000 | .349 | .394 | .457 | .547 |
|  | Integrative_posttest | .349 | 1.000 | .585 | .399 | .554 |
|  | Instrumental_posttest | .394 | .585 | 1.000 | .607 | .559 |
|  | autonomouslearning_posttest | .457 | .399 | .607 | 1.000 | .412 |
|  | Intrinsic_posttest | .547 | .554 | .559 | .412 | 1.000 |
| Sig. (1-tailed) | Englishproficiency_posttest |  | .002 | .000 | .000 | .000 |
|  | Integrative_posttest | .002 |  | .000 | .000 | .000 |
|  | Instrumental_posttest | .000 | .000 |  | .000 | .000 |
|  | autonomouslearning_posttest | .000 | .000 | .000 |  | .000 |
|  | Intrinsic_posttest | .000 | .000 | .000 | .000 |  |
| N | Englishproficiency_posttest | 70 | 70 | 70 | 70 | 70 |
|  | Integrative_posttest | 70 | 70 | 70 | 70 | 70 |
|  | Instrumental_posttest | 70 | 70 | 70 | 70 | 70 |
|  | autonomouslearning_posttest | 70 | 70 | 70 | 70 | 70 |
|  | Intrinsic_posttest | 70 | 70 | 70 | 70 | 70 |

| **Variables Entered/Removed^a^** | | | |
| --- | --- | --- | --- |
| Model | Variables Entered | Variables Removed | Method |
| 1 | Intrinsic_posttest, autonomouslearning_posttest, Integrative_posttest, Instrumental_posttest^b^ |  | Enter |
| a. Dependent Variable: Englishproficiency_posttest | | | |
| b. All requested variables entered. | | | |

| **Model Summary^b^** | | | | | | | | | |
| --- | --- | --- | --- | --- | --- | --- | --- | --- | --- |
| Model | R | R Square | Adjusted R Square | Std. Error of the Estimate | Change Statistics | | | | |
|  |  |  |  |  | R Square Change | F Change | df1 | df2 | Sig. F Change |
| 1 | .604^a^ | .365 | .325 | 6.996 | .365 | 9.323 | 4 | 65 | .000 |
| a. Predictors: (Constant), Intrinsic_posttest, autonomouslearning_posttest, Integrative_posttest, Instrumental_posttest | | | | | | | | | |
| b. Dependent Variable: Englishproficiency_posttest | | | | | | | | | |

| **ANOVA^a^** | | | | | | |
| --- | --- | --- | --- | --- | --- | --- |
| Model | | Sum of Squares | df | Mean Square | F | Sig. |
| 1 | Regression | 1825.346 | 4 | 456.336 | 9.323 | .000^b^ |
|  | Residual | 3181.740 | 65 | 48.950 |  |  |
|  | Total | 5007.086 | 69 |  |  |  |
| a. Dependent Variable: Englishproficiency_posttest | | | | | | |
| b. Predictors: (Constant), Intrinsic_posttest, autonomouslearning_posttest, Integrative_posttest, Instrumental_posttest | | | | | | |

| **Coefficients^a^** | | | | | | | | |
| --- | --- | --- | --- | --- | --- | --- | --- | --- |
| Model | | Unstandardized Coefficients | | Standardized Coefficients | t | Sig. | Collinearity Statistics | |
|  |  | B | Std. Error | Beta |  |  | Tolerance | VIF |
| 1 | (Constant) | 43.970 | 3.784 |  | 11.621 | .000 |  |  |
|  | Integrative_posttest | .075 | 1.194 | .008 | .063 | .950 | .582 | 1.718 |
|  | Instrumental_posttest | -.317 | 1.313 | -.035 | -.242 | .810 | .457 | 2.189 |
|  | autonomouslearning_posttest | 2.654 | 1.132 | .294 | 2.346 | .022 | .623 | 1.604 |
|  | Intrinsic_posttest | 3.926 | 1.132 | .441 | 3.467 | .001 | .604 | 1.657 |
| a. Dependent Variable: Englishproficiency_posttest | | | | | | | | |

| **Collinearity Diagnostics^a^** | | | | | | | | |
| --- | --- | --- | --- | --- | --- | --- | --- | --- |
| Model | | Eigenvalue | Condition Index | Variance Proportions | | | | |
|  |  |  |  | (Constant) | Integrative_posttest | Instrumental_posttest | autonomouslearning_posttest | Intrinsic_posttest |
| 1 | 1 | 4.846 | 1.000 | .00 | .00 | .00 | .00 | .00 |
|  | 2 | .049 | 9.917 | .00 | .17 | .03 | .48 | .24 |
|  | 3 | .046 | 10.312 | .68 | .01 | .30 | .00 | .01 |
|  | 4 | .034 | 11.897 | .01 | .54 | .02 | .03 | .72 |
|  | 5 | .025 | 13.919 | .31 | .28 | .64 | .50 | .02 |
| a. Dependent Variable: Englishproficiency_posttest | | | | | | | | |

| **Residuals Statistics^a^** | | | | | |
| --- | --- | --- | --- | --- | --- |
|  | Minimum | Maximum | Mean | Std. Deviation | N |
| Predicted Value | 54.51 | 75.25 | 64.89 | 5.143 | 70 |
| Residual | -21.627 | 12.133 | .000 | 6.791 | 70 |
| Std. Predicted Value | -2.018 | 2.014 | .000 | 1.000 | 70 |
| Std. Residual | -3.091 | 1.734 | .000 | .971 | 70 |
| a. Dependent Variable: Englishproficiency_posttest | | | | | |


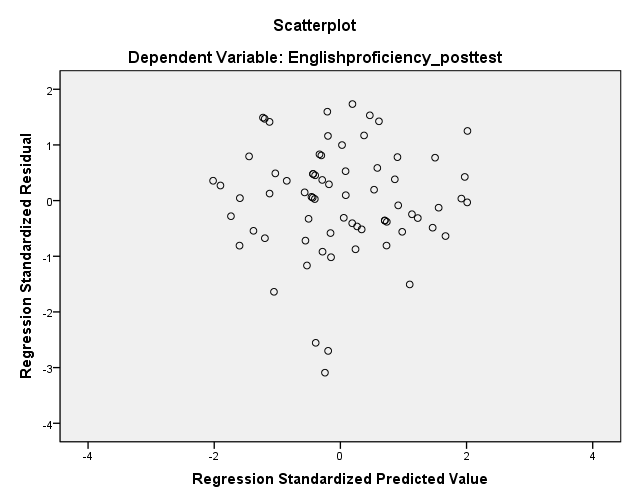

Supplement: S1 File — (ZIP) [file pone.0322094.s001.zip › S1/S1.docx]
