# Supplementary figures and images for: The impact of the flipped classroom on the motivation and academic performance of Chinese college English learners
Source: PLoS One. 2025 May 2;20(5):e0322094. doi: 10.1371/journal.pone.0322094 (PMC12047774; doi:10.1371/journal.pone.0322094)

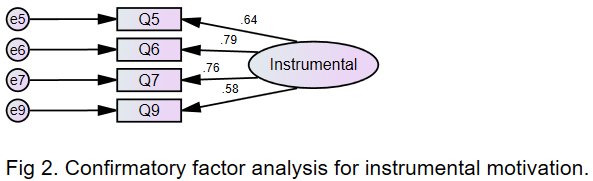

Supplement: S1 File — (ZIP) [file pone.0322094.s001.zip › S1/Fig 2.tif]

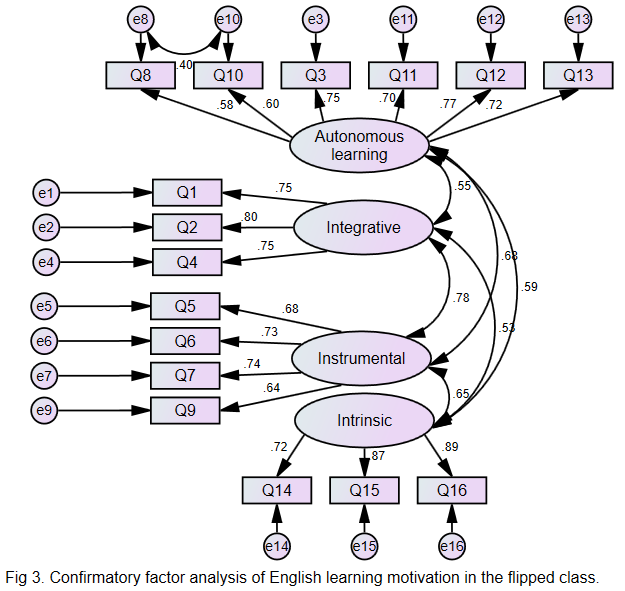

Supplement: S1 File — (ZIP) [file pone.0322094.s001.zip › S1/Fig 3.tif]

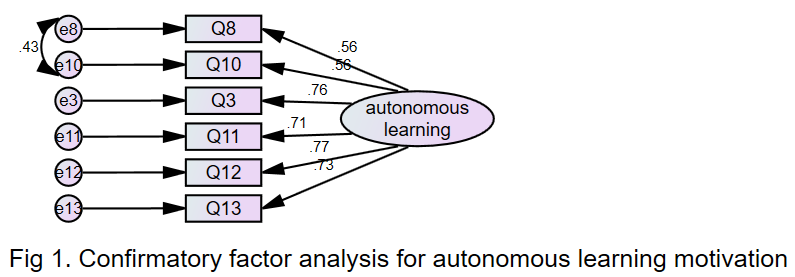

Supplement: S1 File — (ZIP) [file pone.0322094.s001.zip › S1/Fig1.tif]
