# Supplementary material for: The impact of the flipped classroom on the motivation and academic performance of Chinese college English learners
Source: PLoS One. 2025 May 2;20(5):e0322094. doi: 10.1371/journal.pone.0322094 (PMC12047774; doi:10.1371/journal.pone.0322094)
Supplement: S1 File — (ZIP) [file pone.0322094.s001.zip › S1/data sets for research question1.doc]

**1, Differences in pre-test scores of English learning motivation between the experimental class and the control class (1=experimental; 2=control)**

| **Group Statistics** | | | | | |
| --- | --- | --- | --- | --- | --- |
| group | | N | Mean | Std. Deviation | Std. Error Mean |
| Integrative_pretest | 1 | 35 | 2.86 | 0.71 | .12014 |
|  | 2 | 35 | 2.92 | 0.73 | .12381 |
| Instrumental_pretest | 1 | 35 | 2.63 | 0.99 | .16759 |
|  | 2 | 35 | 2.63 | 0.70 | .11836 |
| autonomouslearning_pretest | 1 | 35 | 2.83 | 1.12 | .18929 |
|  | 2 | 35 | 2.72 | 0.84 | .14280 |
| Intrinsic_pretest | 1 | 35 | 2.75 | 0.88 | .14866 |
|  | 2 | 35 | 2.93 | 0.99 | .16725 |
| Totalmotivation_pretest | 1 | 35 | 2.77 | 0.68 | .11468 |
|  | 2 | 35 | 2.80 | 0.58 | .09745 |

| **Independent Samples Test** | | | | | | | | | | |
| --- | --- | --- | --- | --- | --- | --- | --- | --- | --- | --- |
|  | | Levene's Test for Equality of Variances | | t-test for Equality of Means | | | | | | |
|  |  | F | Sig. | t | df | Sig. (2-tailed) | Mean Difference | Std. Error Difference | 95% Confidence Interval of the Difference | |
|  |  |  |  |  |  |  |  |  | Lower | Upper |
| Integrative_pretest | Equal variances assumed | .004 | .949 | -.386 | 68 | .700 | -.06667 | .17251 | -.41091 | .27758 |
|  | Equal variances not assumed |  |  | -.386 | 67.938 | .700 | -.06667 | .17251 | -.41092 | .27759 |
| Instrumental_pretest | Equal variances assumed | 4.999 | .029 | 0.000 | 68 | 1.000 | 0.00000 | .20517 | -.40941 | .40941 |
|  | Equal variances not assumed |  |  | 0.000 | 61.162 | 1.000 | 0.00000 | .20517 | -.41024 | .41024 |
| autonomouslearning_pretest | Equal variances assumed | 5.242 | .025 | .462 | 68 | .646 | .10952 | .23711 | -.36362 | .58267 |
|  | Equal variances not assumed |  |  | .462 | 63.231 | .646 | .10952 | .23711 | -.36427 | .58332 |
| Intrinsic_pretest | Equal variances assumed | .492 | .486 | -.809 | 68 | .422 | -.18095 | .22377 | -.62748 | .26558 |
|  | Equal variances not assumed |  |  | -.809 | 67.077 | .422 | -.18095 | .22377 | -.62759 | .26569 |
| Totalmotivation_pretest | Equal variances assumed | .730 | .396 | -.229 | 68 | .819 | -.03452 | .15049 | -.33482 | .26578 |
|  | Equal variances not assumed |  |  | -.229 | 66.274 | .819 | -.03452 | .15049 | -.33497 | .26592 |

| **Independent Samples Effect Sizes** | | | | | |
| --- | --- | --- | --- | --- | --- |
|  | | Standardizer | Point Estimate | 95% Confidence Interval | |
|  |  |  |  | Lower | Upper |
| Integrative_pretest | Cohen's d | 0.72168 | -0.092 | -0.561 | 0.377 |
|  | Hedges' correction | 0.72976 | -0.091 | -0.555 | 0.373 |
|  | Glass's delta | 0.73247 | -0.091 | -0.559 | 0.379 |
| Instrumental_pretest | Cohen's d | 0.85829 | 0.000 | -0.469 | 0.469 |
|  | Hedges' correction | 0.86790 | 0.000 | -0.463 | 0.463 |
|  | Glass's delta | 0.70024 | 0.000 | -0.469 | 0.469 |
| autonomouslearning_pretest | Cohen's d | 0.99190 | 0.110 | -0.359 | 0.579 |
|  | Hedges' correction | 1.00301 | 0.109 | -0.355 | 0.572 |
|  | Glass's delta | 0.84480 | 0.130 | -0.341 | 0.598 |
| Intrinsic_pretest | Cohen's d | 0.93610 | -0.193 | -0.662 | 0.277 |
|  | Hedges' correction | 0.94659 | -0.191 | -0.655 | 0.274 |
|  | Glass's delta | 0.98949 | -0.183 | -0.652 | 0.289 |
| Totalmotivation_pretest | Cohen's d | 0.62955 | -0.055 | -0.523 | 0.414 |
|  | Hedges' correction | 0.63660 | -0.054 | -0.517 | 0.409 |
|  | Glass's delta | 0.57652 | -0.060 | -0.528 | 0.409 |
|  | | | | | |

**2, Differences in post-test scores of English learning motivation between the experimental class and the control class**

| **Group Statistics** | | | | | |
| --- | --- | --- | --- | --- | --- |
| group | | N | Mean | Std. Deviation | Std. Error Mean |
| Integrative_posttest | 1 | 35 | 3.53 | 0.90 | .15291 |
|  | 2 | 35 | 2.97 | 0.87 | .14678 |
| Instrumental_posttest | 1 | 35 | 3.34 | 0.91 | .15411 |
|  | 2 | 35 | 2.72 | 0.89 | .15090 |
| autonomouslearning_posttest | 1 | 35 | 3.84 | 0.78 | .13230 |
|  | 2 | 35 | 2.80 | 0.79 | .13373 |
| Intrinsic_posttest | 1 | 35 | 3.58 | 0.94 | .15956 |
|  | 2 | 35 | 2.95 | 0.88 | .14790 |
| Totalmotivation_posttest | 1 | 35 | 3.58 | 0.69 | .11693 |
|  | 2 | 35 | 2.86 | 0.64 | .10873 |

| **Independent Samples Test** | | | | | | | | | | |
| --- | --- | --- | --- | --- | --- | --- | --- | --- | --- | --- |
|  | | Levene's Test for Equality of Variances | | t-test for Equality of Means | | | | | | |
|  |  | F | Sig. | t | df | Sig. (2-tailed) | Mean Difference | Std. Error Difference | 95% Confidence Interval of the Difference | |
|  |  |  |  |  |  |  |  |  | Lower | Upper |
| Integrative_posttest | Equal variances assumed | .077 | .783 | 2.651 | 68 | .010 | .56190 | .21195 | .13896 | .98485 |
|  | Equal variances not assumed |  |  | 2.651 | 67.887 | .010 | .56190 | .21195 | .13894 | .98487 |
| Instrumental_posttest | Equal variances assumed | .012 | .913 | 2.881 | 68 | .005 | .62143 | .21568 | .19104 | 1.05181 |
|  | Equal variances not assumed |  |  | 2.881 | 67.970 | .005 | .62143 | .21568 | .19104 | 1.05182 |
| autonomouslearning_posttest | Equal variances assumed | .022 | .882 | 5.569 | 68 | .000 | 1.04762 | .18812 | .67223 | 1.42301 |
|  | Equal variances not assumed |  |  | 5.569 | 67.992 | .000 | 1.04762 | .18812 | .67223 | 1.42301 |
| Intrinsic_posttest | Equal variances assumed | .886 | .350 | 2.889 | 68 | .005 | .62857 | .21757 | .19442 | 1.06272 |
|  | Equal variances not assumed |  |  | 2.889 | 67.612 | .005 | .62857 | .21757 | .19437 | 1.06277 |
| Totalmotivation_posttest | Equal variances assumed | .356 | .553 | 4.477 | 68 | .000 | .71488 | .15967 | .39627 | 1.03350 |
|  | Equal variances not assumed |  |  | 4.477 | 67.644 | .000 | .71488 | .15967 | .39623 | 1.03353 |

| **Independent Samples Effect Sizes** | | | | | |
| --- | --- | --- | --- | --- | --- |
|  | | Standardizer | Point Estimate | 95% Confidence Interval | |
|  |  |  |  | Lower | Upper |
| Integrative_posttest | Cohen's d | 0.88667 | 0.634 | 0.151 | 1.112 |
|  | Hedges' correction | 0.89660 | 0.627 | 0.149 | 1.100 |
|  | Glass's delta | 0.86837 | 0.647 | 0.150 | 1.136 |
| Instrumental_posttest | Cohen's d | 0.90226 | 0.689 | 0.204 | 1.169 |
|  | Hedges' correction | 0.91237 | 0.681 | 0.202 | 1.156 |
|  | Glass's delta | 0.89272 | 0.696 | 0.195 | 1.188 |
| autonomouslearning_posttest | Cohen's d | 0.78696 | 1.331 | 0.808 | 1.846 |
|  | Hedges' correction | 0.79577 | 1.316 | 0.799 | 1.826 |
|  | Glass's delta | 0.79117 | 1.324 | 0.754 | 1.881 |
| Intrinsic_posttest | Cohen's d | 0.91016 | 0.691 | 0.206 | 1.171 |
|  | Hedges' correction | 0.92035 | 0.683 | 0.203 | 1.158 |
|  | Glass's delta | 0.87501 | 0.718 | 0.215 | 1.212 |
| Totalmotivation_posttest | Cohen's d | 0.66795 | 1.070 | 0.565 | 1.569 |
|  | Hedges' correction | 0.67543 | 1.058 | 0.559 | 1.551 |
|  | Glass's delta | 0.64327 | 1.111 | 0.568 | 1.642 |

**3, Differences in English learning motivation between the pre-test and the post-test for the control class**

| **Paired Samples Statistics** | | | | | |
| --- | --- | --- | --- | --- | --- |
|  | | Mean | N | Std. Deviation | Std. Error Mean |
| Pair 1 | integrative_pretest | 2.92 | 35 | 0.73 | .12381 |
|  | integrative_posttest | 2.97 | 35 | 0.87 | .14678 |
| Pair 2 | Instrumental_pretest | 2.63 | 35 | 0.70 | .11836 |
|  | Instrumental_posttest | 2.72 | 35 | 0.89 | .15090 |
| Pair 3 | autonomouslearning_pretest | 2.72 | 35 | 0.84 | .14280 |
|  | autonomouslearning_posttest | 2.80 | 35 | 0.79 | .13373 |
| Pair 4 | Intrinsic_pretest | 2.93 | 35 | 0.99 | .16725 |
|  | Intrinsic_posttest | 2.95 | 35 | 0.88 | .14790 |
| Pair 5 | Totalmotivation_pretest | 2.80 | 35 | 0.58 | .09745 |
|  | Totalmotivation_posttest | 2.86 | 35 | 0.64 | .10873 |

| **Paired Samples Test** | | | | | | | | | |
| --- | --- | --- | --- | --- | --- | --- | --- | --- | --- |
|  | | Paired Differences | | | | | t | df | Sig. (2-tailed) |
|  |  | Mean | Std. Deviation | Std. Error Mean | 95% Confidence Interval of the Difference | |  |  |  |
|  |  |  |  |  | Lower | Upper |  |  |  |
| Pair 1 | integrative_pretest - integrative_posttest | -.04762 | .97062 | .16407 | -.38104 | .28580 | -.290 | 34 | .773 |
| Pair 2 | Instrumental_pretest - Instrumental_posttest | -.09286 | .85982 | .14534 | -.38821 | .20250 | -.639 | 34 | .527 |
| Pair 3 | autonomouslearning_pretest - autonomouslearning_posttest | -.07619 | .69233 | .11702 | -.31401 | .16163 | -.651 | 34 | .519 |
| Pair 4 | Intrinsic_pretest - Intrinsic_posttest | -.01905 | 1.12878 | .19080 | -.40680 | .36870 | -.100 | 34 | .921 |
| Pair 5 | Totalmotivation_pretest - Totalmotivation_posttest | -.05893 | .37170 | .06283 | -.18661 | .06875 | -.938 | 34 | .355 |

| **Paired Samples Effect Sizes** | | | | | | |
| --- | --- | --- | --- | --- | --- | --- |
|  | | | Standardizer | Point Estimate | 95% Confidence Interval | |
|  |  |  |  |  | Lower | Upper |
| Pair 1 | integrative_pretest - integrative_posttest | Cohen's d | 0.97062 | -0.049 | -0.380 | 0.283 |
|  |  | Hedges' correction | 0.98150 | -0.049 | -0.376 | 0.280 |
| Pair 2 | Instrumental_pretest - Instrumental_posttest | Cohen's d | 0.85982 | -0.108 | -0.439 | 0.225 |
|  |  | Hedges' correction | 0.86945 | -0.107 | -0.435 | 0.223 |
| Pair 3 | autonomouslearning_pretest - autonomouslearning_posttest | Cohen's d | 0.69233 | -0.110 | -0.442 | 0.223 |
|  |  | Hedges' correction | 0.70008 | -0.109 | -0.437 | 0.221 |
| Pair 4 | Intrinsic_pretest - Intrinsic_posttest | Cohen's d | 1.12878 | -0.017 | -0.348 | 0.315 |
|  |  | Hedges' correction | 1.14142 | -0.017 | -0.344 | 0.311 |
| Pair 5 | Totalmotivation_pretest - Totalmotivation_posttest | Cohen's d | 0.37170 | -0.159 | -0.491 | 0.176 |
|  |  | Hedges' correction | 0.37586 | -0.157 | -0.485 | 0.174 |

**4, Differences in English learning** motivation between the pre-test and the post-test for the experimental class

| **Paired Samples Statistics** | | | | | |
| --- | --- | --- | --- | --- | --- |
|  | | Mean | N | Std. Deviation | Std. Error Mean |
| Pair 1 | Integrative_pretest | 2.86 | 35 | 0.71 | .12014 |
|  | Integrative_posttest | 3.53 | 35 | 0.90 | .15291 |
| Pair 2 | Instrumental_pretest | 2.63 | 35 | 0.99 | .16759 |
|  | Instrumental_posttest | 3.34 | 35 | 0.91 | .15411 |
| Pair 3 | autonomouslearning_pretest | 2.83 | 35 | 1.12 | .18929 |
|  | autonomouslearning_posttest | 3.84 | 35 | 0.78 | .13230 |
| Pair 4 | Intrinsic_pretest | 2.75 | 35 | 0.88 | .14866 |
|  | Intrinsic_posttest | 3.58 | 35 | 0.94 | .15956 |
| Pair 5 | Totalmotivation_pretest | 2.77 | 35 | 0.68 | .11468 |
|  | Totalmotivation_posttest | 3.58 | 35 | 0.69 | .11693 |

| **Paired Samples Test** | | | | | | | | | |
| --- | --- | --- | --- | --- | --- | --- | --- | --- | --- |
|  | | Paired Differences | | | | | t | df | Sig. (2-tailed) |
|  |  | Mean | Std. Deviation | Std. Error Mean | 95% Confidence Interval of the Difference | |  |  |  |
|  |  |  |  |  | Lower | Upper |  |  |  |
| Pair 1 | Integrative_pretest - Integrative_posttest | -.67619 | .89108 | .15062 | -.98229 | -.37009 | -4.489 | 34 | .000 |
| Pair 2 | Instrumental_pretest - Instrumental_posttest | -.71429 | .99103 | .16751 | -1.05472 | -.37385 | -4.264 | 34 | .000 |
| Pair 3 | autonomouslearning_pretest - autonomouslearning_posttest | -1.01429 | 1.08116 | .18275 | -1.38568 | -.64289 | -5.550 | 34 | .000 |
| Pair 4 | Intrinsic_pretest - Intrinsic_posttest | -.82857 | 1.06449 | .17993 | -1.19424 | -.46291 | -4.605 | 34 | .000 |
| Pair 5 | Totalmotivation_pretest - Totalmotivation_posttest | -.80833 | .64555 | .10912 | -1.03009 | -.58658 | -7.408 | 34 | .000 |

| **Paired Samples Effect Sizes** | | | | | | |
| --- | --- | --- | --- | --- | --- | --- |
|  | | | Standardizer | Point Estimate | 95% Confidence Interval | |
|  |  |  |  |  | Lower | Upper |
| Pair 1 | Integrative_pretest - Integrative_posttest | Cohen's d | 0.8911 | -0.759 | -1.131 | -0.378 |
|  |  | Hedges' correction | 0.9011 | -0.750 | -1.119 | -0.373 |
| Pair 2 | Instrumental_pretest - Instrumental_posttest | Cohen's d | 0.9910 | -0.721 | -1.089 | -0.344 |
|  |  | Hedges' correction | 1.0021 | -0.713 | -1.077 | -0.340 |
| Pair 3 | autonomouslearning_pretest - autonomouslearning_posttest | Cohen's d | 1.0812 | -0.938 | -1.332 | -0.534 |
|  |  | Hedges' correction | 1.0933 | -0.928 | -1.317 | -0.529 |
| Pair 4 | Intrinsic_pretest - Intrinsic_posttest | Cohen's d | 1.0645 | -0.778 | -1.153 | -0.395 |
|  |  | Hedges' correction | 1.0764 | -0.770 | -1.140 | -0.390 |
| Pair 5 | Totalmotivation_pretest - Totalmotivation_posttest | Cohen's d | 0.6456 | -1.252 | -1.691 | -0.802 |
|  |  | Hedges' correction | 0.6528 | -1.238 | -1.673 | -0.793 |
