# Supplementary material for: The impact of the flipped classroom on the motivation and academic performance of Chinese college English learners
Source: PLoS One. 2025 May 2;20(5):e0322094. doi: 10.1371/journal.pone.0322094 (PMC12047774; doi:10.1371/journal.pone.0322094)
Supplement: S1 File — (ZIP) [file pone.0322094.s001.zip › S1/data sets for research question2.doc]

**1, Differences in pre-test scores of English proficiency between the experimental and the control class**

| **Group Statistics** | | | | | | | | | | |  | | | | | |
| --- | --- | --- | --- | --- | --- | --- | --- | --- | --- | --- | --- | --- | --- | --- | --- | --- |
| group | | | | N | Mean | | Std. Deviation | | | Std. Error Mean |  | | | | | |
| Englishproficiency_pretest | | 1 | | 35 | 61.20 | | 7.843 | | | 1.326 |  | | | | | |
|  |  | 2 | | 35 | 62.23 | | 9.852 | | | 1.665 |  | | | | | |
| **Independent Samples Test** | | | | | | | | | | | | | | | | |
|  | | | Levene's Test for Equality of Variances | | | | | t-test for Equality of Means | | | | | | | | |
|  |  |  | F | | | Sig. | | t | df | | | Sig. (2-tailed) | Mean Difference | Std. Error Difference | 95% Confidence Interval of the Difference | |
|  |  |  |  |  |  |  |  |  |  |  |  |  |  |  | Lower | Upper |
| Englishproficiency_pretest | Equal variances assumed | | 2.312 | | | .133 | | -.483 | 68 | | | .630 | -1.029 | 2.129 | -5.276 | 3.219 |
|  | Equal variances not assumed | |  | | |  | | -.483 | 64.747 | | | .631 | -1.029 | 2.129 | -5.280 | 3.223 |

| **Independent Samples Effect Sizes** | | | | | |
| --- | --- | --- | --- | --- | --- |
|  | | Standardizer | Point Estimate | 95% Confidence Interval | |
|  |  |  |  | Lower | Upper |
| Englishproficiency_pretest | Cohen's d | 8.905 | -0.116 | -0.584 | 0.354 |
|  | Hedges' correction | 9.004 | -0.114 | -0.578 | 0.350 |
|  | Glass's delta | 9.852 | -0.104 | -0.573 | 0.366 |

**2, Differences in post-test scores of English proficiency between the experimental and the control class**

| **Group Statistics** | | | | | |
| --- | --- | --- | --- | --- | --- |
| group | | N | Mean | Std. Deviation | Std. Error Mean |
| Englishproficiency_posttest | 1 | 35 | 68.06 | 7.996 | 1.352 |
|  | 2 | 35 | 61.71 | 7.913 | 1.338 |

| **Independent Samples Test** | | | | | | | | | | |
| --- | --- | --- | --- | --- | --- | --- | --- | --- | --- | --- |
|  | | Levene's Test for Equality of Variances | | t-test for Equality of Means | | | | | | |
|  |  | F | Sig. | t | df | Sig. (2-tailed) | Mean Difference | Std. Error Difference | 95% Confidence Interval of the Difference | |
|  |  |  |  |  |  |  |  |  | Lower | Upper |
| Englishproficiency_posttest | Equal variances assumed | .000 | .997 | 3.336 | 68 | .001 | 6.343 | 1.902 | 2.548 | 10.137 |
|  | Equal variances not assumed |  |  | 3.336 | 67.993 | .001 | 6.343 | 1.902 | 2.548 | 10.137 |

| **Independent Samples Effect Sizes** | | | | | |
| --- | --- | --- | --- | --- | --- |
|  | | Standardizer | Point Estimate | 95% Confidence Interval | |
|  |  |  |  | Lower | Upper |
| Englishproficiency_posttest | Cohen's d | 7.955 | 0.797 | 0.307 | 1.282 |
|  | Hedges' correction | 8.044 | 0.789 | 0.304 | 1.268 |
|  | Glass's delta | 7.913 | 0.802 | 0.291 | 1.302 |

**3, Differences in the scores of English proficiency between the pre-test and the post-test for the control class**

| **Paired Samples Statistics** | | | | | |
| --- | --- | --- | --- | --- | --- |
|  | | Mean | N | Std. Deviation | Std. Error Mean |
| Pair 1 | Englishproficiency_pretest | 62.23 | 35 | 9.852 | 1.665 |
|  | Englishproficiency_posttest | 61.71 | 35 | 7.913 | 1.338 |

| **Paired Samples Test** | | | | | | | | | |
| --- | --- | --- | --- | --- | --- | --- | --- | --- | --- |
|  | | Paired Differences | | | | | t | df | Sig. (2-tailed) |
|  |  | Mean | Std. Deviation | Std. Error Mean | 95% Confidence Interval of the Difference | |  |  |  |
|  |  |  |  |  | Lower | Upper |  |  |  |
| Pair 1 | Englishproficiency_pretest - Englishproficiency_posttest | .514 | 9.189 | 1.553 | -2.642 | 3.671 | .331 | 34 | .743 |

| **Paired Samples Effect Sizes** | | | | | | |
| --- | --- | --- | --- | --- | --- | --- |
|  | | | Standardizer | Point Estimate | 95% Confidence Interval | |
|  |  |  |  |  | Lower | Upper |
| Pair 1 | Englishproficiency_pretest - Englishproficiency_posttest | Cohen's d | 9.189 | 0.056 | -0.276 | 0.387 |
|  |  | Hedges' correction | 9.292 | 0.055 | -0.273 | 0.383 |

**4, Differences in** the scores of English proficiency between the pre-test and the post-test for the experimental class

| **Paired Samples Statistics** | | | | | |
| --- | --- | --- | --- | --- | --- |
|  | | Mean | N | Std. Deviation | Std. Error Mean |
| Pair 1 | Englishproficiency_pretest | 61.20 | 35 | 7.843 | 1.326 |
|  | Englishproficiency_posttest | 68.06 | 35 | 7.996 | 1.352 |

| **Paired Samples Test** | | | | | | | | | |
| --- | --- | --- | --- | --- | --- | --- | --- | --- | --- |
|  | | Paired Differences | | | | | t | df | Sig. (2-tailed) |
|  |  | Mean | Std. Deviation | Std. Error Mean | 95% Confidence Interval of the Difference | |  |  |  |
|  |  |  |  |  | Lower | Upper |  |  |  |
| Pair 1 | Englishproficiency_pretest - Englishproficiency_posttest | -6.857 | 9.696 | 1.639 | -10.188 | -3.527 | -4.184 | 34 | .000 |

| **Paired Samples Effect Sizes** | | | | | | |
| --- | --- | --- | --- | --- | --- | --- |
|  | | | Standardizer | Point Estimate | 95% Confidence Interval | |
|  |  |  |  |  | Lower | Upper |
| Pair 1 | Englishproficiency_pretest - Englishproficiency_posttest | Cohen's d | 9.696 | -0.707 | -1.074 | -0.332 |
|  |  | Hedges' correction | 9.804 | -0.699 | -1.062 | -0.328 |
