# Supplementary material for: The impact of the flipped classroom on the motivation and academic performance of Chinese college English learners
Source: PLoS One. 2025 May 2;20(5):e0322094. doi: 10.1371/journal.pone.0322094 (PMC12047774; doi:10.1371/journal.pone.0322094)
Supplement: S1 File — (ZIP) [file pone.0322094.s001.zip › S1/datasets for research question3.doc]

**Predictability of motivational factors in English proficiency (RQ3)**

| **Descriptive Statistics** | | | |
| --- | --- | --- | --- |
|  | Mean | Std. Deviation | N |
| Englishproficiency_posttest | 64.89 | 8.519 | 70 |
| Integrative_posttest | 3.2524 | .92459 | 70 |
| Instrumental_posttest | 3.0321 | .94880 | 70 |
| autonomouslearning_posttest | 3.3190 | .94270 | 70 |
| Intrinsic_posttest | 3.2667 | .95739 | 70 |

| **Correlations** | | | | | | |
| --- | --- | --- | --- | --- | --- | --- |
|  | | Englishproficiency_posttest | Integrative_posttest | Instrumental_posttest | autonomouslearning_posttest | Intrinsic_posttest |
| Pearson Correlation | Englishproficiency_posttest | 1.000 | .349 | .394 | .457 | .547 |
|  | Integrative_posttest | .349 | 1.000 | .585 | .399 | .554 |
|  | Instrumental_posttest | .394 | .585 | 1.000 | .607 | .559 |
|  | autonomouslearning_posttest | .457 | .399 | .607 | 1.000 | .412 |
|  | Intrinsic_posttest | .547 | .554 | .559 | .412 | 1.000 |
| Sig. (1-tailed) | Englishproficiency_posttest |  | .002 | .000 | .000 | .000 |
|  | Integrative_posttest | .002 |  | .000 | .000 | .000 |
|  | Instrumental_posttest | .000 | .000 |  | .000 | .000 |
|  | autonomouslearning_posttest | .000 | .000 | .000 |  | .000 |
|  | Intrinsic_posttest | .000 | .000 | .000 | .000 |  |
| N | Englishproficiency_posttest | 70 | 70 | 70 | 70 | 70 |
|  | Integrative_posttest | 70 | 70 | 70 | 70 | 70 |
|  | Instrumental_posttest | 70 | 70 | 70 | 70 | 70 |
|  | autonomouslearning_posttest | 70 | 70 | 70 | 70 | 70 |
|  | Intrinsic_posttest | 70 | 70 | 70 | 70 | 70 |

| **Variables Entered/Removed^a^** | | | |
| --- | --- | --- | --- |
| Model | Variables Entered | Variables Removed | Method |
| 1 | Intrinsic_posttest, autonomouslearning_posttest, Integrative_posttest, Instrumental_posttest^b^ |  | Enter |
| a. Dependent Variable: Englishproficiency_posttest | | | |
| b. All requested variables entered. | | | |

| **Model Summary^b^** | | | | | | | | | |
| --- | --- | --- | --- | --- | --- | --- | --- | --- | --- |
| Model | R | R Square | Adjusted R Square | Std. Error of the Estimate | Change Statistics | | | | |
|  |  |  |  |  | R Square Change | F Change | df1 | df2 | Sig. F Change |
| 1 | .604^a^ | .365 | .325 | 6.996 | .365 | 9.323 | 4 | 65 | .000 |
| a. Predictors: (Constant), Intrinsic_posttest, autonomouslearning_posttest, Integrative_posttest, Instrumental_posttest | | | | | | | | | |
| b. Dependent Variable: Englishproficiency_posttest | | | | | | | | | |

| **ANOVA^a^** | | | | | | |
| --- | --- | --- | --- | --- | --- | --- |
| Model | | Sum of Squares | df | Mean Square | F | Sig. |
| 1 | Regression | 1825.346 | 4 | 456.336 | 9.323 | .000^b^ |
|  | Residual | 3181.740 | 65 | 48.950 |  |  |
|  | Total | 5007.086 | 69 |  |  |  |
| a. Dependent Variable: Englishproficiency_posttest | | | | | | |
| b. Predictors: (Constant), Intrinsic_posttest, autonomouslearning_posttest, Integrative_posttest, Instrumental_posttest | | | | | | |

| **Coefficients^a^** | | | | | | | | |
| --- | --- | --- | --- | --- | --- | --- | --- | --- |
| Model | | Unstandardized Coefficients | | Standardized Coefficients | t | Sig. | Collinearity Statistics | |
|  |  | B | Std. Error | Beta |  |  | Tolerance | VIF |
| 1 | (Constant) | 43.970 | 3.784 |  | 11.621 | .000 |  |  |
|  | Integrative_posttest | .075 | 1.194 | .008 | .063 | .950 | .582 | 1.718 |
|  | Instrumental_posttest | -.317 | 1.313 | -.035 | -.242 | .810 | .457 | 2.189 |
|  | autonomouslearning_posttest | 2.654 | 1.132 | .294 | 2.346 | .022 | .623 | 1.604 |
|  | Intrinsic_posttest | 3.926 | 1.132 | .441 | 3.467 | .001 | .604 | 1.657 |
| a. Dependent Variable: Englishproficiency_posttest | | | | | | | | |

| **Collinearity Diagnostics^a^** | | | | | | | | |
| --- | --- | --- | --- | --- | --- | --- | --- | --- |
| Model | | Eigenvalue | Condition Index | Variance Proportions | | | | |
|  |  |  |  | (Constant) | Integrative_posttest | Instrumental_posttest | autonomouslearning_posttest | Intrinsic_posttest |
| 1 | 1 | 4.846 | 1.000 | .00 | .00 | .00 | .00 | .00 |
|  | 2 | .049 | 9.917 | .00 | .17 | .03 | .48 | .24 |
|  | 3 | .046 | 10.312 | .68 | .01 | .30 | .00 | .01 |
|  | 4 | .034 | 11.897 | .01 | .54 | .02 | .03 | .72 |
|  | 5 | .025 | 13.919 | .31 | .28 | .64 | .50 | .02 |
| a. Dependent Variable: Englishproficiency_posttest | | | | | | | | |

| **Residuals Statistics^a^** | | | | | |
| --- | --- | --- | --- | --- | --- |
|  | Minimum | Maximum | Mean | Std. Deviation | N |
| Predicted Value | 54.51 | 75.25 | 64.89 | 5.143 | 70 |
| Residual | -21.627 | 12.133 | .000 | 6.791 | 70 |
| Std. Predicted Value | -2.018 | 2.014 | .000 | 1.000 | 70 |
| Std. Residual | -3.091 | 1.734 | .000 | .971 | 70 |
| a. Dependent Variable: Englishproficiency_posttest | | | | | |
